# Applying Multiple Analyses to Quantify Changes in the Global, Regional, and National Burden of Tuberculosis From 1990 to 2021 and the Forecast Until 2035

**DOI:** 10.1155/cjid/8899003

**Published:** 2025-11-20

**Authors:** Qi Zeng, Depeng Jiang

**Affiliations:** Department of Respiratory Medicine, Second Affiliated Hospital of Chongqing Medical University, Chongqing 400010, China

**Keywords:** age–period–cohort, Bayesian age–period–cohort, decomposition analysis, frontier analysis, health inequality, tuberculosis

## Abstract

**Background:**

Comprehensive assessments of changes in the burden of tuberculosis (TB) can inform health strategies.

**Methods:**

Disability-adjusted life years (DALYs) data were obtained from the Global Burden of Disease database. We used estimated annual percentage changes (EAPCs) to evaluate trends; decomposition analysis to examine the effects of population growth, aging, and epidemiological changes; age–period–cohort model to measure the age, period, and cohort effect; health inequality analysis to assess absolute and relative inequalities; frontier analysis to explore the lowest potential achievable burden based on sociodemographic index (SDI); and Bayesian age–period–cohort model to project future trends.

**Results:**

Central Asia (EAPC, 7.82, 95% confidence interval [CI], 6.24–9.43) and Eastern Europe (EAPC, 9.42, 95% CI, 7.33–11.55) had notable increases in TB-related DALYs from 1990 to 1999. Globally, the contributions of population aging, growth, and epidemiological change of TB burden from 1990 to 2021 were −17.29%, −77.75%, and 195.04%, respectively. Children aged 0–4 years have an extremely high risk of TB-related DALYs. Period and cohort effects of TB continuously declined. The slope index of inequality increased from −5183.27 (95% CI, −5636.08 to −4730.46) in 1990 to −1729.88 (95% CI, −1927.35 to −1532.41) in 2021, suggesting a reduction in absolute socioeconomic inequality. Nevertheless, the concentration index showed a minimal decrease from −0.516 (95% CI, −0.571 to −0.461) in 1990 to −0.532 (95% CI, −0.603 to −0.461) in 2021, indicating an increase in relative socioeconomic inequality. Most regions have substantial potential to reduce their TB burden, with the Central African Republic having the highest potential. For low-SDI countries, Niger may serve as an exemplar, and poverty elimination could have profound effects. The burden of TB is projected to decline, but it is unlikely to meet the goals of the End TB Strategy.

**Conclusion:**

TB continues to pose a critical health challenge that necessitates the implementation of effective control strategies.

## 1. Introduction

Tuberculosis (TB) remains a long-standing and pervasive infectious disease that poses a significant threat to global health [1]. In 2021, an estimated 10.6 million people contracted TB, and there were approximately 1.6 million TB-related deaths [2]. Over the past three decades, substantial investments and numerous interventions have been implemented to eliminate TB [2]. These efforts have contributed to notable reductions in TB incidence and death [2]. However, the first milestone set by the World Health Organization (WHO) for TB control was not achieved globally, highlighting the urgent need for sustained, targeted, and evidence-based interventions [3].

The Global Burden of Disease (GBD) study is a comprehensive initiative aimed at quantifying health loss globally and over time [2]. Among its key metrics, disability-adjusted life years (DALYs) is a widely recognized composite indicator of disease burden. Prior research within the GBD framework focusing on TB has mainly presented general disease trends and demographic characteristics, lacking detailed analyses to investigate the underlying causes of changes [4–8]. Such analyses are crucial for shaping public health policies [9]. Despite substantial global population growth, aging, and epidemiological shifts, with decreased communicable disease mortality in the past three decades, the specific impacts of these demographic and epidemiological factors on TB burden have not been thoroughly explored [10]. While age–period–cohort (APC) analysis has been used in previous studies to examine TB morbidity and mortality from a cross-sectional and longitudinal perspective in different regions, no study has employed the APC model to assess global TB-related DALYs [11]. It has been noted that TB burden tends to decrease as the sociodemographic index (SDI) levels increase, yet there is a lack of quantitative evaluations regarding the evolving socioeconomic disparities over time [12]. Although the SDI level significantly influences TB burden, countries with similar SDI levels may still exhibit notably different TB outcomes [12, 13]. The question persists as to which countries have not achieved or have successfully achieved the most effective prevention and control of TB given their available resources and development status.

In this study, we extracted TB-related DALYs from the GBD database to (i) describe the global, regional, and national burden of TB over the past three decades; (ii) assess the effects of population aging, growth, and epidemiological transition; (iii) examine the influence of age, period, and cohort effects; (iv) evaluate socioeconomic inequalities in TB burden across countries and territories; (v) estimate the potentially achievable burden of TB based on SDI; and (vi) forecast future trends of TB.

## 2. Materials and Methods

### 2.1. Data Source and Definition

Data were obtained from the GBD 2021, a systematic comparative analysis disaggregated by age, sex, and geographic location at specific time points [2]. According to the GBD 2021, TB is an infectious disease caused by *Mycobacterium tuberculosis*, encompassing pulmonary and extrapulmonary forms [2]. The GBD study used the International Classification of Diseases, 10th revision, as a reference, with TB codes ranging from A15-A19.9, B90-B90.9, K67.3, K93.0, M49.0, N74.1, P37.0, and U84.3. The Cause of Death Ensemble modeling strategy, a Bayesian hierarchical model distinguished by its superior out-of-sample predictive validity, was employed to estimate TB-related mortality. Age- and sex-specific TB incidence, prevalence, and mortality were modeled using DisMod-MR 2.1, a Bayesian meta-regression tool. Detailed methodologies related to the GBD study have been published elsewhere [2]. The burden of TB was measured using DALYs, which combines years of life lost (YLLs) and years lived with disability (YLDs). YLLs were calculated by multiplying the number of deaths by the standard life expectancy at the age of death. YLDs were determined by multiplying the prevalence of TB sequelae by disability weights. Crude DALYs rate, also known as all ages rate, was used to remove the influence of population size. It was calculated by the following algorithm: crude DALYs rate = DALYs number/population. To account for changes in population growth and aging, the age-standardized rate (ASR) of DALYs was calculated:

(1) $$\begin{array}{c} \text{ASR of DALYs}\propto \overset{n}{\underset{\text{agei}}{\sum }}\frac{{\text{DALYs⁣number}}_{\text{agei}}}{{\text{population}}_{\text{agei}}}*{{\text{standard}}_{\text{population}}}_{\text{agei}}, \end{array}$$

where agei represents the *i*^th^ age range with a span of 5 years old. The SDI, ranging from 0 to 1, was also used to represent a composite of per capita income, mean years of schooling, and fertility rate among individuals under 25 years. Higher SDI values indicate more developed societies. The 204 countries or territories were classified into five categories based on their SDI values: low (SDI: 0–0.466), low-middle (SDI: 0.466–0.619), middle (SDI: 0.619–0.712), high-middle (SDI: 0.712–0.810), and high (SDI: 0.810–1). In addition, they were grouped into 21 GBD regions based on their socioeconomic and geographic characteristics.

### 2.2. Estimated Annual Percentage Change (EAPC)

EAPC has been widely used to assess temporal trends [14, 15]. In this study, EAPC and its 95% confidence interval (CI) were derived from a linear regression model, expressed as *y* = *α* + *βx* + *ε*, where *x* represents the calendar year and *y* is the natural logarithm of DALYs. The formula for calculating the EAPC was EAPC = 100 × (exp (*β*) − 1). An EAPC with a 95% CI above zero indicates an increasing trend, whereas an EAPC with a 95% CI below zero indicates a decreasing trend. Other values indicate that the DALYs remained stable over time. The 32-year period was divided into three intervals (1990–1999, 2000–2009, and 2010–2021), to calculate the EAPCs for each interval, as well as the overall EAPCs at the global, regional, and national levels. In addition, a world map was created to visualize the EAPCs at a national level.

### 2.3. APC Analysis

The APC model is widely applied in epidemiology to disentangle the contributions of age, period, and cohort effects in chronic disease trends. This model assumes a log-linear Poisson distribution over a Lexis observed rate diagram. Due to the perfect linear dependency among age, period, and cohort—where [Cohort] = [Period] − [Age]—it is statistically impossible to estimate their independent effects within a regression model, a challenge known as the identification problem. To address this, the intrinsic estimator (IE) method was employed to separate their relative contributions. The APC model can be expressed as

(2) $$\begin{array}{c} \rho =\log\left(M\right)=\mu +\alpha a+\pi p+\gamma c+\varepsilon , \end{array}$$

where *ρ* represents the logarithmic transformation of the expected rate, and *μ* and *ε* denote the intercept and random error, respectively. The terms *α*, *π*, and *γ* correspond to the effects of age, period, and cohort [16].

For our analysis, we utilized the APC web tool to generate model parameters [17]. The ASR of TB DALYs were categorized into successive 5-year age groups (0–4, 5–9,…, 95–99) and consecutive 5-year periods (1992–1996, 1997–2001,…, 2017–2021), with corresponding sequential birth cohorts. The GBD 2021 population data was also aggregated into 5-year age groups and periods. Given that the GBD study produces annual estimates with 5-year age groups, we set 1992 as the starting year for the APC analysis and computed the average population for each 5 years. In total, 20 five-year age groups and periods were used as data inputs for the APC model. The Wald chi-squared test was applied to assess the statistical significance of the APC parameters.

### 2.4. Decomposition Analysis

Decomposition analysis was used to divide the changes in DALYs into three components: age structure, population growth, and epidemiological changes [18, 19]. The DALYs were calculated using the following formula:

(3) $$\begin{array}{c} {\text{DALYs}}_{a,p,e,y}=\overset{20}{\underset{i=1}{\sum }}a_{i,y}*p_{y}*e_{i,y}. \end{array}$$

DALYs_*a*,*p*,*e*,*y*_ represents the number of DALYs cumulated by age structure, population growth, and DALYs rate (which represents epidemiological changes) in year *y*; *a*_*i*,*y*_ is the proportion of the population for the age group *i* at year *y* (20 age groups, from under 5 years–95 years and older); *p*_*y*_ is the population size at year *y*; and *e*_*i*,*y*_ is represented by the ASR of DALYs by age group *i* in year *y*.

We hypothesized that the effect of a one-factor change based on the other two factors would remain unchanged. Therefore, the impact of population aging can be calculated as follows.

(4) $$\begin{array}{c} {\text{Effect}}_{a_{2021}}=\left[\frac{a_{2021}*p_{1990}*e_{1990}+a_{2021}*p_{2021}*r_{2021}}{3}+\frac{a_{2021}*p_{1990}*r_{2021}+a_{2021}*p_{2021}*r_{1990}}{6}\right]-\left[\frac{a_{1990}*p_{1990}*r_{1990}+a_{1990}*p_{2021}*r_{2021}}{3}+\frac{a_{1990}*p_{1990}*r_{2021}+a_{1990}*p_{2021}*r_{1990}}{6}\right]. \end{array}$$

### 2.5. Health Inequality Analysis

The slope index of inequality (SII) and the concentration index (CI) are two complex measures recommended by the WHO used to assess absolute and relative inequality, respectively [13, 20]. To calculate the SII, the ASR of TB DALYs was regressed against the midpoint of the weighted population across 204 countries or territories ranked by SDI from lowest to highest using a linear regression model. The slope of this regression represents the SII, and negative values suggest that the ASR of TB DALYs is higher in disadvantaged populations. CI is also known as the Gini Index. The CI is twice the area between the concentration curve and the hypothetical line of equality (45° diagonal). In this curve, the *x*-axis represents the weighted population ordered by the SDI, whereas the *y*-axis represents the cumulative proportion of the ASR of TB DALYs for each country or territory. A concentration curve that lies above the line of equality indicates that the burden is concentrated in the less advantaged regions. CI with an absolute value of 0.4–0.5 represents a high level of relative inequality, with an absolute value > 0.5 indicating great disparity.

### 2.6. Frontier Analysis

Data envelopment analysis (DEA), a nonparametric frontier analysis, was used to investigate the lowest achievable ASR of TB DALYs relative to the level of development of a country or territory, as measured by the SDI [18, 21]. Unlike stochastic frontier analysis, which is widely used in healthcare scenarios, DEA does not require a prespecified functional form for the relationship between variables. However, DEA assumes no measurement errors and is sensitive to outliers. To address this limitation, the free disposal hull method was used, which is a more flexible variant of the DEA that relaxes the assumption of convexity in the frontier and allows for the inclusion of measurement errors. Outliers, defined as superefficient points, were excluded from the calculation. To further minimize the modeling errors, 100 bootstrapped samples of the data were generated, incorporating year, location, and other relevant covariates. Each bootstrapped sample comprises of randomly selected locations with replacements. The mean frontier value for the ASR of DALYs was calculated from these 100 bootstrapped samples. Locally estimated scatterplot smoothing regression was applied to create a smooth frontier boundary (polynomial degree, 1; span, 0.1). The difference between the actual ASR of DALYs and the frontier values—termed the “effective difference”—represents the potential health gains a country or territory could achieve, given the current level of social development. A significant gap below the frontier value for a given SDI indicates that there is substantial room for improvement in reducing the ASR of TB DALYs considering the development context.

### 2.7. Bayesian APC (BAPC) Analysis

The BAPC model is widely used to project future disease burdens because it requires no parametric assumptions, exhibits higher coverage with a relatively lower error rate, and the probabilistic forecasts generated by the BAPC model are well calibrated [22, 23]. In this study, the ASRs of TB DALYs were classified into 5-year age-group intervals (0–4, 5–9,…, 95–99 years). Population data from the GBD 2021 study, along with standard population projections extending to 2035, were aggregated into the 5-year age group intervals. The R codes used to conduct these analyses are provided in the supporting information.

## 3. Results

### 3.1. Global, Regional, and National Trends of TB-Related DALYs From 1990 to 2021

Between 1990 and 2021, the global TB-related DALYs have decreased in number and in the ASR, with EAPCs of −1.94 (95% CI, −2.09–−1.78) for number and −3.5 (95% CI, −3.67–-3.33) for ASR. High–middle- and low-SDI regions had a stable or mildly upward trend in the number of DALYs from 1990 to 1999, with EAPCs of 0.23 (95% CI, −0.15–0.6) and 0.3 (95% CI, 0.12–0.48), respectively. In terms of ASR, Central Asia, Eastern Europe, and Southern sub-Saharan Africa had upward trends from 1990 to 1999, with EAPCs of 7.82 (95% CI, 6.24–9.43), 9.42 (95% CI, 7.33–11.55), and 0.43 (95% CI, −0.63– 1.5), respectively. The upward trend in Southern Sub-Saharan Africa continued from 2000 to 2009, with an EAPC of 1.5 (95% CI, 0.01–3.02). All regions had decreasing trends from 2010 to 2021, with Central Asia and Eastern Europe having the most pronounced declines, with EAPCs of −7.81 (95% CI, −8.16–−7.47) and −10.68 (95% CI, −11.32– −10.03), respectively (Supporting Table S1).

**Figure 1:**
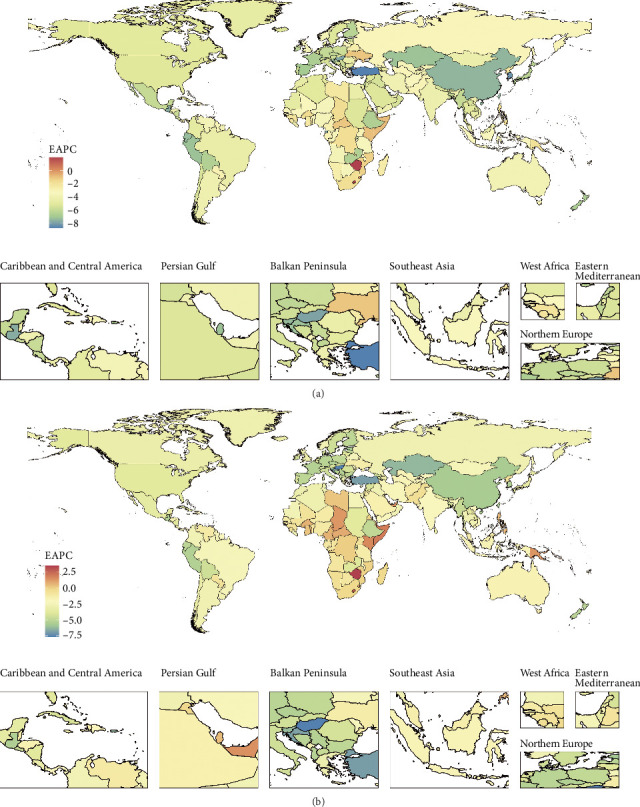
Estimated annual percentage changes (EAPCs) of tuberculosis in the numbers (a) and age-standardized rates (b) of disability-adjusted life years (DALYs) during 1990–2021 at the national level.

At the national level, 23 countries had increasing trends in the numbers of DALYs from 1990 to 2021, with Zimbabwe (EAPC, 3.41; 95% CI, 2.73–4.1), Lesotho (EAPC, 2.66; 95% CI, 2.03–3.29), and Somalia (EAPC, 1.91; 95% CI, 1.68–2.14) having particularly significant increases (Figure 1(a)). Overall, 202 countries or territories had decreasing trends in the ASR of DALYs from 1990 to 2021, with Turkey having the greatest decline (EAPC, −8.72; 95% CI, −9.22–−8.21), followed by the Maldives (EAPC, −8.53; 95% CI, −8.99–−8.07) and the Republic of Korea (EAPC, −8.28; 95% CI, −8.52–−8.04) (Figure 1(b)).

### 3.2. Decomposition Analysis

Epidemiological change (195.04%) played a significant role in the reduction of the burden of TB from 1990 to 2021. During this period, the contribution of population aging was most pronounced in middle-SDI quintiles (−37.67%), followed by low–middle (−26.53%)- and high (−25.09%)-SDI quintiles. In contrast, population growth was a significant contributor to changes in DALYs in low (−245.04%)-, low–middle (−103.88%)-, and middle (−58.44%)-SDI quintiles (Table 1). From 1990 to 1999, epidemiological change notably contributed to an increase in TB-related DALYs in Central Asia (93.34%) and Eastern Europe (99.69%). From 2000 to 2009, the contribution of population aging was most significant in middle-SDI regions (−42.45%), while population growth was most pronounced in low-SDI regions (−152.32%). At the regional level, population aging was pronounced in Australasia, high-income Asia Pacific, and Eastern Europe, while population growth was significant in sub-Saharan Africa and Oceania. Between 2010 and 2021, population aging was a significant contributor to high-SDI regions (−38.67%), followed by middle (−31.23%)- and low–middle (−29.37%)-SDI regions. Population growth was most pronounced in low-SDI regions (−121.41%) during this period. At the regional level, population aging was significant in Australasia, Central Latin America, high-income Asia Pacific, high-income North America, Oceania, and Southern Latin America, while population growth was pronounced in Australasia, Caribbean, high-income North America, Oceania, Latin America, and sub-Saharan Africa (Supporting Table S2). Supporting Table S3 presents the decomposition analysis at the national level.

### 3.3. APC Analysis

**Figure 2:**
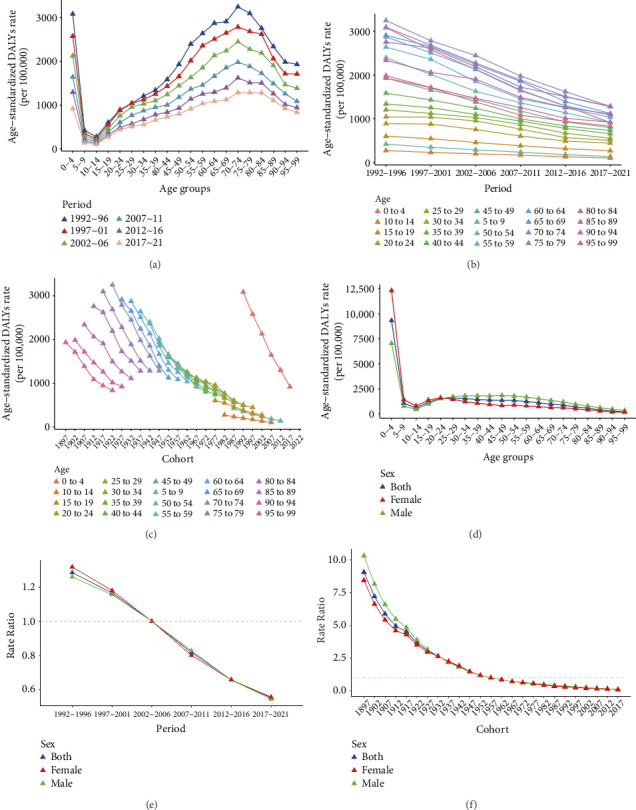
Age-period-cohort (APC) analysis. Age-specific (a), period-specific (b), and cohort-specific (c) trends of age-standardized rate (ASR) of tuberculosis (TB) disability-adjusted life years (DALYs) from 1992 to 2021. And age (d), period (e), and cohort (f) effect.

The age-specific ASR of DALYs exhibited a bimodal distribution, with peaks observed in the 0–4 and 70–74 age groups. The rates declined sharply from the 0–4 age group to the 5–9 age group, followed by a gradual increase from the 10–14 age group to the 70–74 age group, after which they declined again (Figure 2(a)). The period-specific ASR of DALYs demonstrated a decline across all age groups from 1992 to 2021 (Figure 2(b)). The cohort-specific ASR of DALYs also showed a downward trend across all birth cohorts, with a particularly sharp decline among early cohorts born between 1897 and 1967. Notably, the ASR in the 0–4 age group was high and exhibited the most pronounced decline over time (Figure 2(c)).

The longitudinal age curve showed that the 0–4 age groups have the highest ASR of DALYs, with 7049 (95% CI, 6756–7355) per 100,000 in males and 12315 (95% CI, 11786–12868) per 100,000 in females. It then exhibited a sharp decline in the 5–9 age group and slightly increased from the 10–14 age group to the 20–24 age group. After that, it gradually declined to the oldest age group. The ASR of DALYs was higher in females before the 15–19 age group and higher in males after that (Figure 2(d)). The period effect demonstrated a steady decline from 1992 to 2021, with the highest risk observed in the 1992–1996 period (RR = 1.28, 95% CI, 1.26–1.31) and the lowest risk in the 2017–2021 period (RR = 0.55, 95% CI, 0.54–0.56). The period effect followed a similar trend across both genders (Figure 2(e)). The cohort effect revealed a decreasing risk of TB with more recent birth cohorts, though the declining trend flattened after the 1957–1961 cohort. The cohort effect was more pronounced in men among pre-1927 cohorts (Figure 2(g)). The APC model demonstrated a statistically significant difference, as confirmed by the Wald chi-square test (Supporting Table S4).

### 3.4. Measurement of Health Inequality

**Figure 3:**
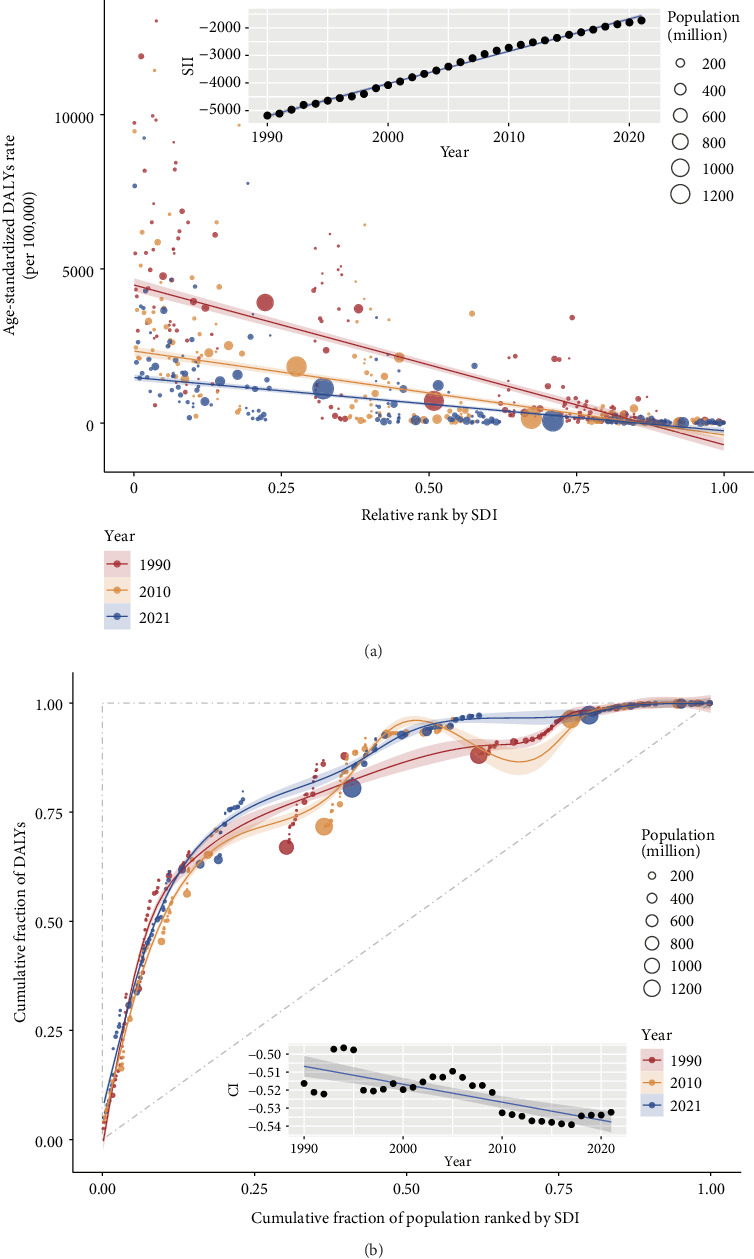
Health inequality analysis. (a) Absolute socioeconomic inequality in the age-standardized rate (ASR) of tuberculosis (TB) disability-adjusted life years (DALYs) across countries or territories in 1990, 2000, and 2021. (b) Relative socio-economic inequality in the ASR of TB DALYs across countries or territories in 1990, 2000, and 2021. The trend line (blue) of the black dots illustrates the trend in the slope index of inequality (SII) or concentration index (CI) from 1990 to 2021. The red, orange, and blue dots signify different countries or territories with sizes corresponding to varying population sizes.

The SII was −5183.27 (95% CI, −5636.08 to −4730.46) in 1990, −2719.60 (95% CI, −3033.62 to −2405.59) in 2010, and −1729.88 (95% CI, −1927.35 to −1532.41) in 2021, representing a negative association between the ASR of TB-related DALYs and SDIs. The SIIs increased from 1990 to 2021, indicating that absolute socioeconomic inequality narrowed over the 32 years (Figure 3(a)). The CI was −0.516 (95% CI, −0.571 to −0.461) in 1990, −0.533 (95% CI, -0.600 to −0.468) in 2010, and -0.532 (95% CI, −0.603 to −0.461) in 2021, indicating high socioeconomic inequality. The CIs had a minor decrease from 1990 to 2021, indicating an increase in relative socioeconomic inequality (Figure 3(b)). The SIIs and CIs are presented in Supporting Table S5.

### 3.5. Frontier Analysis

**Figure 4:**
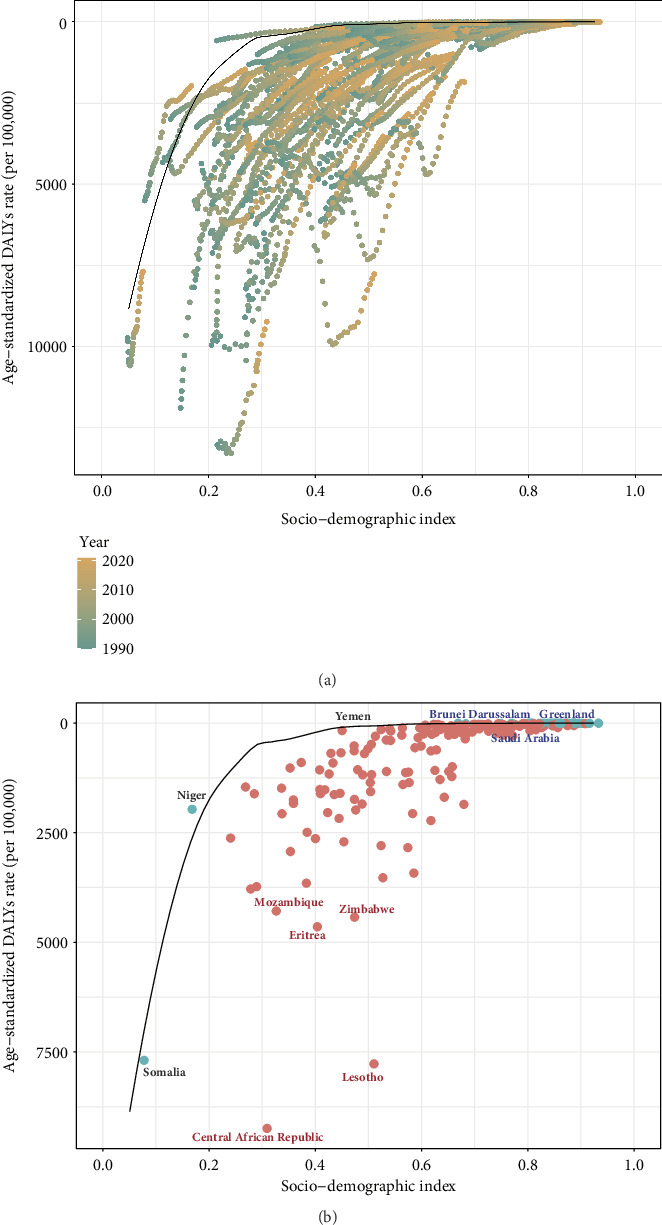
Frontier analysis. (a) The frontier line, delineated in black, illustrates the potentially achievable age-standardized rate (ASR) of tuberculosis (TB) disability-adjusted life years (DALYs) based on the sociodemographic index (SDI). The dots represent the actual ASR of DALYs from 1990 to 2021, with a color gradient ranging from green to orange indicating the progression of years from 1990 to 2021. (b) The five countries with the largest effective differences, three countries with small effective differences and low SDI (< 0.466), and three countries with relatively large effective differences and high SDI (> 0.810) are labeled. The red dots indicate countries or territories with an effective difference exceeding 10, whereas the green dots represent countries or territories with an effective difference of less than 10.

The potentially achievable ASR of TB-related DALYs, represented by the frontier line, showed a pronounced downward trend as the SDI increased for values below 0.4. In contrast, for SDI values exceeding 0.5, the ASR gradually declined (Figure 4(a)). By 2021, 37 countries with an effective difference of less than 10 were classified as overperformers. In general, smaller effective differences were associated with higher SDI levels. However, countries such as Somalia and Niger demonstrated minimal effective differences, despite having lower SDI values. Among nations with SDIs below 0.466, Yemen (87.88) also had a relatively low effective difference. The Central African Republic (8,841.3), Lesotho (7710.48), Eritrea (4468.12), Zimbabwe (4350.97), and Mozambique (3,880) were the countries with the most pronounced effective differences. Notably, among the countries or territories with SDI values greater than 0.810, Saudi Arabia (149.30), Brunei Darussalam (133.42), and Greenland (101.12) had the highest effective differences (Figure 4(b)).

### 3.6. Global DALYs of Drug-Resistant TB From 1990 to 2021

**Figure 5:**
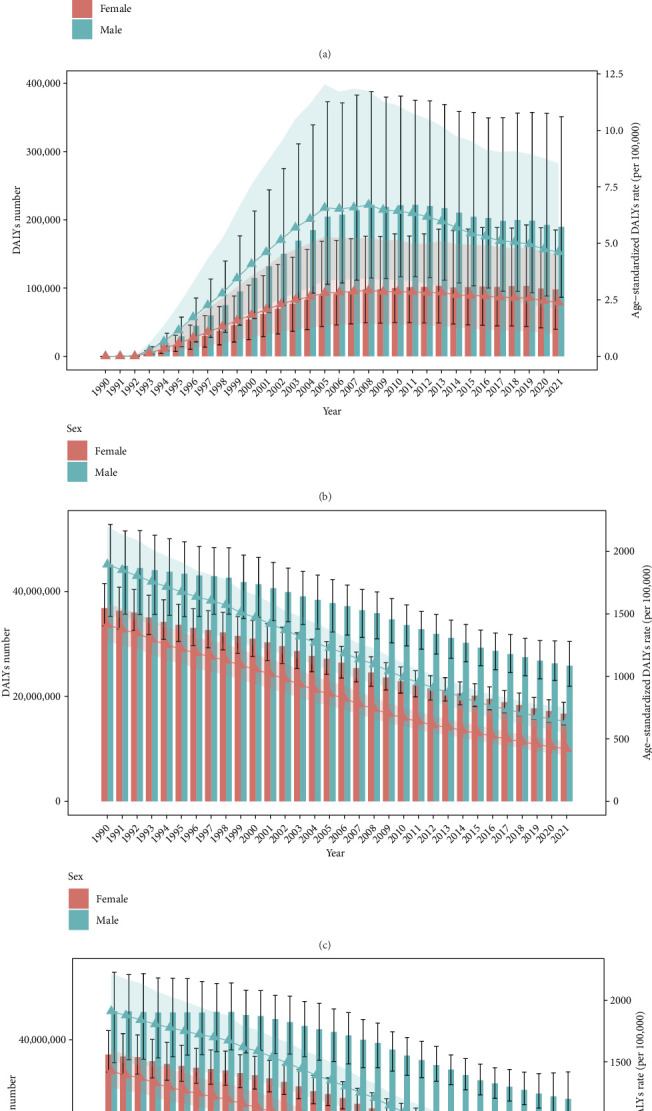
Global disability-adjusted life years (DALYs) of multidrug-resistant tuberculosis (TB) without extensive drug resistance (a), extensively drug-resistant TB (b), drug-susceptible TB (c), and TB (d).

Globally, for multidrug-resistant tuberculosis without extensive drug resistance (MDR-TB), the number of DALYs in males increased from 393,258 (95% CI: 147,576–974,827) in 1990 to 2,504,513 (95% CI: 1,044,212–4,884,136) in 2021. In females, the number of DALYs increased from 283,633 (95% CI: 103,088–671,480) in 1990 to 1,620,948 (95% CI: 663,397–3,135,883) in 2021. The ASR of DALYs rose from 16.83 (95% CI: 6.31–41.87) per 100,000 in males and 11.08 (95% CI: 3.99–26.24) per 100,000 in females in 1990 to 61.64 (95% CI: 25.73–120.57) per 100,000 in males and 40.52 (95% CI: 16.92–78.63) per 100,000 in females in 2021. The ASR trend for MDR-TB peaked in 2003, followed by a gradual decline (Figure 5(a)). For extensively drug-resistant tuberculosis (XDR-TB), the number of DALYs in males reached 189,827 (95% CI: 86,537–351,058) in 2021, while in females, it rose to 97,909 (95% CI: 39,631–185,046). The ASR of DALYs in 2021 was 4.61 (95% CI: 2.10–8.53) per 100,000 in males and 2.34 (95% CI: 0.97–4.50) per 100,000 in females. The ASR trend for XDR-TB increased until 2005, fluctuated slightly until 2008, and subsequently declined (Figure 5(b)). In contrast, DALYs attributable to drug-susceptible TB consistently declined from 1990 to 2021. In 2021, drug-susceptible TB accounted for 90.56% and 90.68% of the total TB-related DALYs in males and females, respectively (Figures 5(c) and 5(d)).

### 3.7. Projections of TB-Related DALYs Until 2035

**Figure 6:**
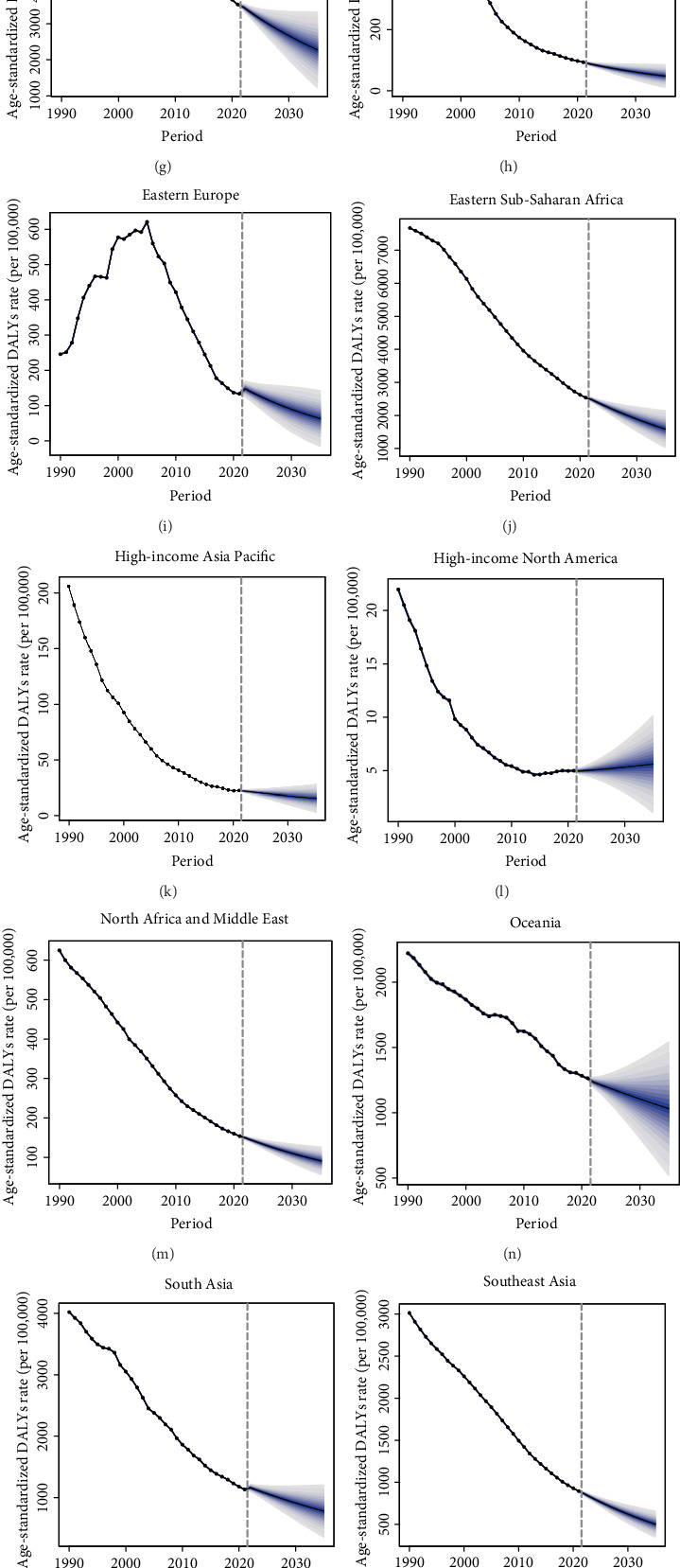
Projections of future burden of tuberculosis until 2035.

The ASR of the TB-related DALYs will decline in most regions by 2035, except for High-income North America, which will show a slight upward trend (Figure 6). The ASR of TB-related incidence is expected to decrease to 96.04 per 100,000 by 2025, 89.54 per 100,000 by 2030, and 83.22 per 100,000 by 2035. Simultaneously, the number of TB-related deaths will decline to 1.15 million by 2025, 1.1 million by 2030, and 1.05 million by 2035. However, it is projected that Central Latin America, High-income North America, High-income Asia Pacific, Oceania, and Southern sub-Saharan Africa will experience an increase in TB-related mortalities (Supporting Table S6).

## 4. Discussion

In this study, multiple analyses were applied to quantitatively evaluate the global, regional, and national burden of TB over the past 32 years, focusing on the effects of population aging, growth, and epidemiological change, the influence of age, period, and cohort effect, health inequality, and the lowest achievable burden of TB based on current SDI status. The findings of this study may contribute to the epidemiology of TB and provide insights into resource allocation and policy formulation.

Since the early 1990s, substantial initiatives to reduce the burden of TB have been implemented worldwide, including controlling risk factors, increasing Bacillus Calmette-Guerin (BCG) immunization coverage, advancing diagnosis and treatment technology, avoiding catastrophic costs, and establishing local TB programs and communities [1, 2]. Despite these advancements, the burden of TB increased in Central Asia and Eastern Europe between 1990 and 1999, likely attributed to the societal upheaval following the dissolution of the Soviet Union in the 1990s, leading to a weakening of TB control efforts [14]. Moreover, individuals with TB in former Soviet states experienced a high risk of developing drug-resistant forms [24]. Southern sub-Saharan Africa also witnessed an increase in TB burden from 1990 to 1999. Nevertheless, downward trends have occurred in these regions during the last decade, with Eastern Europe showing the most significant decrease. This decline underscores the effectiveness of TB management strategies, such as preventive therapy, which can reduce the risk of multidrug-resistant TB development by up to 90% [25].

Most regions had a continuous downward trend in the burden of TB from 1990 to 2021. However, the declining trend, as measured by EAPC, is less pronounced in absolute numbers than in ASRs, primarily due to the effects of population aging and growth. These demographic factors also contribute to the regional and temporal variations in the burden of TB. Population growth has been more influential than aging in most regions, particularly Oceania and sub-Saharan Africa. Alongside climate change and antimicrobial resistance, population growth is recognized as a major driver of the emergence of novel pathogens and the resurgence of infections that were previously under control [26]. Increased population density can exacerbate poor ventilation and overcrowded living conditions, which facilitate the transmission of TB and strain healthcare systems, hindering effective TB control. In Latin America and the Caribbean, TB imposes a heavier burden in countries with faster urban population growth [27]. Nevertheless, global annual live births have declined at a rate of 0.6% since 2016, with only 94 countries maintaining total fertility rates above the replacement level of 2.1 in 2021 [28]. This includes 44 of the 46 countries in sub-Saharan Africa, which collectively accounted for 29.2% of global live births and the proportion is predicted to increase [29]. This trend would result in a demographically divided world, where much of the planet faces challenges associated with low fertility, many low-income countries still grapple with issues stemming from high fertility. Restricting population growth in lower-income regions might contribute to mitigating the burden of TB, as well as alleviating problems such as food shortage and global warming [28]. However, sustained reduction of fertility could lead to a shrinking labor force, potentially undermining economic growth.

Population aging also plays a significant role in the burden of TB, particularly in high-SDI regions and, more recently, in middle-SDI regions. In developed countries with a low TB burden, higher TB incidence and mortality have been observed among the vulnerable elderly population [30]. In China, the prevalence of TB from 2015 to 2019, along with the number of newly diagnosed TB cases among individuals aged over 60 years, has increased, likely in association with the country's aging population [31]. Age-specific trends indicate that the TB burden increases from the 10–14 age group to the 70–74 age group, probably due to cumulative exposure to *Mycobacterium tuberculosis*, air pollution, and other age-related determinants [32]. Additionally, older individuals are more susceptible to delayed diagnosis, progression to active TB, and adverse treatment outcomes due to weakened immune systems and atypical clinical presentations [33]. Given the rapid global aging trend and increasing life expectancy, targeted strategies and interventions are essential for TB elimination in older adults [10]. Key measures include preventive treatment for high-risk groups, active case finding, close monitoring, timely and effective treatment, and integration of comprehensive healthcare resources [30]. However, aging itself is not a primary risk factor for TB, and the age-related effect on TB burden among older individuals is not as pronounced, as indicated by APC analysis [34]. In age-specific TB trends, the burden declines after the 70–74 age group, primarily due to the effects of remote infection and the selective survival of healthier elderly individuals [35].

According to single-factor descriptive analysis, age-specific TB-related DALYs exhibit a bimodal distribution, with peaks in the 0–4 and 70–74 age groups. However, after controlling for period and cohort effects, the TB-related DALYs remains disproportionately high among children aged 0–4 years. Early-life respiratory infections can have profound and lasting effects on lung function trajectories. Even following successful TB treatment, children may experience persistent respiratory impairments, growth deficits, and a diminished quality of life [36]. In children aged 0–4 years, TB infection is commonly associated with reduced tidal volume and peak tidal expiratory flow [36]. Notably, the risk of TB-related DALYs declines sharply from the 0–4 age group to the 5–9 age group. In the European Union, the highest proportion of pediatric TB cases has been reported among children aged 1–4 years [37]. A mathematical modeling study estimated that 239,000 children younger than 15 years died from TB in 2015, with 80% of these deaths occurring in children younger than 5 years [38]. Young children (< 5 years) are at significantly higher risk for severe and disseminated TB, conditions associated with high mortality rates [39]. In contrast, pulmonary TB in children aged 5–9 typically manifests as intrathoracic lymph node disease, which is generally less severe due to limited parenchymal involvement [39]. Moreover, children under 5 years of age exhibit impaired innate immune cell function and a Th2-skewed immune response, rendering them more susceptible to TB [39]. Additionally, due to the paucibacillary nature of pulmonary TB in children under 10 years, the rate of missed diagnoses is high [40]. Since microbiological confirmation is more likely in severe TB cases, the 0–4 age group may have a higher rate of confirmed TB diagnoses than the 5–9 age group [41]. Some previous studies suggest that individuals under 15 years of age generally have a lower overall TB risk due to the protective effect of the BCG vaccine [11, 16]. Our findings highlight the substantial risk of TB-related DALYs among children aged 0–4 years, underscoring the urgent need for effective interventions to improve long-term health outcomes in this vulnerable population.

Gender disparities in TB-related DALYs are evident and vary across different age groups. According to longitudinal age curves, females exhibit a higher risk of TB-related DALYs before the 15–19 age group, particularly among young children. Several studies also reported that adolescent females are at higher risk of TB progression and experience heavier mortality [42, 43]. Girls are more prone to malnutrition, a critical risk factor for TB, and are less likely to receive adequate treatment following TB infection, particularly in regions with lower SDI levels [44, 45]. Although males generally exhibit Th1-skewed immune responses, which are considered immunosuppressive, they tend to mount exaggerated responses to Th1-inducing pathogens such as *Mycobacterium tuberculosis* [39]. Human immunodeficiency virus (HIV) infection is a significant risk factor for TB, and in 2019, females accounted for 65.8% of new HIV cases among individuals aged 10–24 years [46]. Social and biological factors such as early sexual debut, adolescent pregnancy, and sexual violence increase the vulnerability of female adolescents to HIV infection, consequently raising their susceptibility to TB [47]. In contrast, males exhibit a higher risk of TB after the 15–19 age group, particularly among middle-aged men. Compared to women, men are more frequently exposed to established TB risk factors such as smoking, alcohol consumption, and occupational hazards [2, 48]. Furthermore, men tend to present with more severe clinical manifestations, including higher rates of hemoptysis, heavier pulmonary damage, and poorer microbiological and treatment outcomes [48]. Delayed healthcare-seeking behavior and lower adherence to anti-TB therapy are also more common among men, contributing to worse disease outcomes [49]. In 2019, among HIV-negative individuals, TB incident cases and deaths were 1.10 and 0.34 million higher in men than in women, while among HIV-positive individuals, TB incident cases and deaths were 81100 and 6250 higher in women than in men [47]. Generally, the burden of TB is more concentrated in males; however, more attention should be paid to females before adulthood, especially for HIV-positive individuals.

The period effect exhibited a consistent decline from 1990 to 2021, mainly attributable to the implementation of effective TB control strategies, as well as global advancements in economic development and medical resources [1, 2, 4]. Regarding the cohort effect, more recent birth cohorts demonstrated a lower risk of TB. Earlier birth cohorts were likely exposed to more significant social unrest, economic downturns, and childhood malnutrition, factors that could have influenced TB susceptibility later in life and even in subsequent generations [50]. However, the rate of decline in risks of TB-related DALYs decelerated during the most recent period (2017–2021) compared to earlier periods, and similarly, the decline was slower in later birth cohorts than in their predecessors. With the improvement of health monitoring systems and diagnostic techniques, many unreported and undiagnosed TB cases have been detected in recent years [2, 47]. However, these findings also suggest that current TB elimination strategies may be insufficient to sustain the previous pace of progress.

Despite significant progress in expanding health services for TB, the disease continues to impose a heavy toll on the poorest and most marginalized populations, remaining one of the leading causes of death in low-income countries. In these areas, poverty, inadequate healthcare systems, and co-infection with HIV are considerable barriers to TB control [12]. Quantifying socioeconomic inequalities in TB and tracking their temporal changes are essential for developing equity-oriented, evidenced-based policies to achieve universal health coverage, which seeks to ensure that all individuals with TB can access affordable, high-quality healthcare services [13]. The CIs, which reflect relative socioeconomic inequalities in TB, showed a minimal decrease from −0.516 in 1990 to −0.532 in 2021, indicating high disparities and representing a progress of 1.6% toward inequality. A similar trend was observed in China, where CIs for TB incidence, prevalence, and mortality all decreased, suggesting heavier inequalities of TB across provinces [51]. The relative socioeconomic inequalities in TB remain striking, surpassing those of HIV, which has CIs of approximately −0.4 [52]. Furthermore, the inequalities of HIV have reduced from 2000 to 2019, primarily due to the continuing support of international organizations [52]. Tackling inequalities is essential for advancing Sustainable Development Goals for both HIV and TB [12]. While both diseases are more prevalent in less-developed regions, the persistent inequalities in TB suggest that current efforts to address disparities in TB are insufficient, and TB may require more focused attention than HIV to effectively reduce inequalities. Nevertheless, the SIIs for TB have continued to increase from 1990 to 2021, indicating a reduction in absolute socioeconomic inequalities. When reflecting on health inequality, it does not distinguish the significance of absolute versus relative metrics of inequality, as absolute inequality represents the magnitude of the difference, and relative inequality reflects the proportional difference [20, 53, 54]. Arithmetically, this combination could occur when countries with low and high baseline burdens have similar decreasing rates [55]. The reduction of absolute inequalities is primarily attributable to initiatives enhancing healthcare for underserved populations [12]. For instance, total spending on TB in low- and middle-income countries (LMICS) has increased at an annual rate of 3.9% from 2000 to 2017 [56]. However, investment in TB remains far from achieving global targets, and out-of-pocket expenditures remain high in underserved areas [56]. It is estimated that if universal health coverage were achieved, the percentage of families facing catastrophic costs due to TB could be reduced by at least 50% in half of affected countries [12, 57]. In addition to financial investment, social protection programs—such as health education to improve treatment adherence and psychological support to reduce stigma—are also critical components in reducing inequalities of TB [58].

Although TB disproportionately affects lower-SDI regions, frontier analysis has demonstrated that the potential to reduce the burden of TB exists across all levels of the SDI spectrum. Notably, several high-SDI countries or territories, such as Saudi Arabia, Brunei Darussalam, and Greenland, have relatively substantial potential to reduce their burden of TB, suggesting that other factors may counterbalance advances in health associated with sociodemographic prosperity. For instance, the abundant oil resources in Saudi Arabia and Brunei Darussalam have driven economic growth; however, occupational hazards, such as exposure to respirable dust, pose significant public health risks and contribute to TB transmission. In these settings, implementing effective measures, such as wet spray misting and engineering controls, is crucial to protect workers from TB infection [59]. In Saudi Arabia, the overall prevalence of TB infection remains 17%, with the mass gathering during the Hajj season potentially facilitating TB transmission [60]. Despite the existence of an established TB control program, Brunei Darussalam has experienced stagnant TB incidence since 2004 [61]. Although TB treatment is free of charge in Greenland, it still has a high burden of TB, which may be associated with delays in diagnosis [62]. Common risk factors among TB-affected populations in high-income areas include immigration, unemployment, incarceration, alcohol abuse, and intravenous drug use [63]. Therefore, enhancing social conditions and optimizing treatment strategies for vulnerable cohorts are imperative. Additionally, mass screening programs with extensive coverage and rapid intervention in high-burden communities may effectively interrupt TB transmission [64].

Certain low-SDI countries, such as Niger, demonstrated exceptional performance in TB control despite constrained resources, reflecting the notion, in many GBD studies, that development is not destiny [18, 19]. Since 2008, Niger has implemented the Shorter Treatment Regimen, nationwide, for rifampicin-resistant TB, a strategy that has proven to be feasible and successful [65]. In 2020, Niger launched a pilot program to establish the first 65 one-stop units that provide dual care to address TB-HIV co-infection, with support from WHO [66]. Niger may serve as an exemplar for other countries with similar development statuses to align policies and leverage available resources. For instance, the Central African Republic possessed the highest potential to reduce the burden of TB among 204 countries or territories, despite having a higher SDI value than Niger. Additionally, the frontier line suggests that the potentially achievable burden of TB decreased remarkably when the SDI fell below approximately 0.4. Similarly, a previous modeling study projected an 84.3% reduction in global TB incidence through the elimination of extreme poverty and expansion of social protection coverage [67]. Therefore, aggressive policies aimed at poverty eradication in low-SDI regions may have a profound impact on reducing the burden of TB. Nevertheless, as the frontier line indicates, the elimination of TB becomes attainable when SDI exceeds approximately 0.5, suggesting that countries with moderate development status should implement targeted measures to combat TB.

The emergence of novel *Mycobacterium tuberculosis* strains, including MDR-TB and XDR-TB, poses a critical global health challenge, particularly affecting regions with lower SDI levels [68]. The DALYs attributable to MDR-TB and XDR-TB showed an overall upward trend from 1990 to 2021, with a pronounced rise between 1990 and 2005, followed by a gradual decline in recent years. The upward trend may be closely linked to strengthened antimicrobial resistance surveillance systems and improved access to rapid diagnostic technologies [69]. Despite diagnosis, a significant proportion of patients with drug-resistant TB remain untreated due to the high cost and limited availability of effective medications [69]. Moreover, treatment success rates remain relatively low, contributing to a heavy disease burden [69]. Furthermore, under conditions of prolonged drug exposure, *Mycobacterium tuberculosis* strains may evolve to exhibit greater transmissibility, increased virulence, and enhanced drug resistance [70]. In recent years, continued advancements in surveillance systems, diagnostic tools, and therapeutic regimens have contributed to improved prevention and control of drug-resistant TB [69]. Notably, the burden of MDR-TB and XDR-TB is disproportionately higher among males. Beyond the factors discussed previously in this paper, alcohol abuse has been identified as a significant risk factor, particularly among middle-aged men, and is associated with increased disease severity [68]. Men are generally more likely to engage in excessive alcohol consumption than women, which may contribute to the heavier burden in this population [68]. Drug-resistant TB can result in long-lasting physical, psychological, and economic sequelae for affected individuals [69]. Therefore, it is imperative to ensure that accurate diagnosis and effective treatment for all forms of drug-resistant TB are accessible to those in need [71]. Additionally, efforts must be made to minimize the emergence of resistance to newly introduced therapies [69]. Although the overall decreases in TB burden are primarily driven by reductions in drug-susceptible TB, the lack of intensified measures to combat drug-resistant TB may halt further declines in the global TB burden [72].

The WHO has set ambitious targets to reduce the incidence rate of TB by 90% and TB-related mortality by 95% by 2035, compared with 2015 levels [3]. Our projections indicate that, worldwide, the milestones and targets are unlikely to be met, with the possibility of an increase in TB-related mortality in certain regions. The COVID-19 pandemic has significantly strained healthcare systems, leading to delays in TB diagnosis and impeding effective TB treatment [73]. This has reversed the progress made by international efforts to reduce the burden of TB prior to 2019, despite potential contributions to TB transmission reduction from public health measures like mask-wearing for COVID-19 [2]. Particularly concerning is the worldwide decrease in reported TB cases, linked to disruptions in TB services during lockdowns, heightening the risk of TB transmission within households and escalating mortality rates [2, 73]. It is projected that the COVID-19 pandemic could elevate TB mortality by 5%–10% over the next five years [74]. Additionally, the diversion of resources and funding towards the COVID-19 pandemic may undermine TB control programs [73]. Despite significant annual investments toward TB elimination, current funding levels remain insufficient to meet global targets. In 2022, an estimated US$5.8 billion was allocated to alleviate the TB burden, representing only 44% of the global target of US$13 billion [1]. Furthermore, LMICs received US$1 billion less in TB funding compared to 2018 [1]. It is crucial to sustain focused efforts and allocate adequate resources to mitigate the impact of COVID-19 and achieve meaningful progress in the fight against TB [75].

This study had several limitations. First, analysis of the burden of TB primarily relies on the accuracy of GBD estimates, which may be limited by data availability in some low-income countries lacking effective health supervision systems. In these cases, the results were generated by mathematical models using available coefficients. Second, macro-level assessments may not adequately capture micro-level trends, for instance, significant subnational variations can be overlooked in countries with extensive territories. Third, although the BAPC model offers several advantages over other models, such as the ability to provide uncertainty estimates and manage heterogeneity, future research might consider incorporating additional predictive models to validate and extend our projections.

## 5. Conclusions

In conclusion, global TB-related DALYs have significantly decreased since the 1990s, accompanied by a reduction in absolute socioeconomic disparities. Nevertheless, substantial relative inequalities persist. To accelerate progress, region-specific, age- and sex-sensitive interventions are essential. In regions with lower SDI, interventions like controlling rapid population growth and implementing effective poverty alleviation measures may help alleviate the TB burden. Conversely, higher-SDI regions need to address the challenges posed by aging populations. Apart from the elderly, targeted interventions for children under five are essential for ensuring long-term health improvements. Additionally, special attention may be warranted for adolescent females and adult males. Despite ongoing interventions, most regions still have significant potential for reducing the TB burden relative to their current SDI levels. Furthermore, the escalating threat of drug-resistant TB should not be underestimated. While the global TB burden is anticipated to decline, it is unlikely to meet the goals of the End TB Strategy. Targeted actions and sufficient resources are critical to mitigate the impact of the COVID-19 pandemic and enhance TB management.

## Figures and Tables

**Table 1:** Change in disability-adjusted life years of tuberculosis decomposed by three population-level determinants from 1990 to 2021.

Location	Overall difference	Change due to population-level determinants (% contribution to the total change)		
		Population aging	Population growth	Epidemiological change
Global	−35702310.75	6172383.88 (−17.29%)	27757674.15 (−77.75%)	−69632368.78 (195.04%)
SDI region				
High SDI	−682221.86	171149.53 (−25.09%)	156591.92 (−22.95%)	−1009963.32 (148.04%)
High–middle SDI	−2648637.14	579238.7 (−21.87%)	613917.51 (−23.18%)	−3841793.35 (145.05%)
Middle SDI	−9833216.19	3703872.55 (−37.67%)	5746312.51 (−58.44%)	−19283401.25 (196.1%)
Low–middle SDI	−14891713.29	3951350.31 (−26.53%)	15470036.49 (−103.88%)	−34313100.08 (230.42%)
Low SDI	−7629486	−687043.72 (9.01%)	18694970.03 (−245.04%)	−25637412.31 (336.03%)

Abbreviation: SDI, sociodemographic index.

## Data Availability

The data that support the findings of this study are openly available in the GBD database at https://vizhub.healthdata.org/gbd-results/.

## Supporting Information

Supporting Table S1: The estimated annual percentage changes and their 95% confidence intervals of disability-adjusted life years of tuberculosis from 1990 to 2021 at the global and regional levels.

Supporting Table S2: Change in disability-adjusted life years of tuberculosis decomposed by three population-level determinants at the global and regional levels.

Supporting Table S3: Change in disability-adjusted life years of tuberculosis decomposed by three population-level determinants from 1990 to 2021 at the national level.

Supporting Table S4: Wald chi-square tests for estimable functions in the age-period-cohort model.

Supporting Table S5: The slope index of inequality, concentration index, and their 95% confidence intervals for the age-standardized rate of disability-adjusted life years of tuberculosis from 1990 to 2021.

Supporting Table S6: Projections of future burden of tuberculosis until 2035.

R codes used to conduct these analyses.
