# Supplementary material for: Applying Multiple Analyses to Quantify Changes in the Global, Regional, and National Burden of Tuberculosis From 1990 to 2021 and the Forecast Until 2035
Source: Can J Infect Dis Med Microbiol. 2025 Nov 20;2025:8899003. doi: 10.1155/cjid/8899003 (PMC12659984; doi:10.1155/cjid/8899003)
Supplement: Supporting Information — Additional supporting information can be found online in the Supporting Information section. [file 8899003.f1.zip › Supplementary_Materials.docx]

## Applying multiple analyses to quantify changes in the global, regional, and national burden of tuberculosis from 1990 to 2021 and the forecast until 2035

Supplementary Materials

1. Supplementary Table S1: The estimated annual percentage changes and their 95% confidence intervals of disability-adjusted life-years of tuberculosis from 1990 to 2021 at the global and regional levels.

2. Supplementary Table S2: Change in disability-adjusted life-years of tuberculosis decomposed by three population-level determinants at the global and regional levels.

3. Supplementary Table S3: Change in disability-adjusted life-years of tuberculosis decomposed by three population-level determinants from 1990 to 2021 at the national level.

4. Supplementary Table S4: Wald Chi-Square tests for estimable functions in the age-period-cohort model

5. Supplementary Table S5: The slope index of inequality, concentration index, and their 95% confidence intervals for the age-standardized rate of disability-adjusted life-years of tuberculosis from 1990 to 2021.

6. Supplementary Table S6: Projections of future burden of tuberculosis until 2035.

7. R codes used to conduct these analyses.

1. Supplementary Table S1: The estimated annual percentage changes and their 95% confidence intervals of disability-adjusted life-years (DALYs) of tuberculosis from 1990 to 2021 at the global and regional levels.

|  | **Number of DALYs** | | | | **ASR of DALYs** | | | | | | |
| --- | --- | --- | --- | --- | --- | --- | --- | --- | --- | --- | --- |
|  | 1990-2021 | 1990-1999 | 2000-2009 | 2010-2021 |  | 1990-2021 | | 1990-1999 | 2000-2009 | 2010-2021 | |
| **Global** | -1.94  (-2.09 to -1.78) | -0.56  (-0.62 to -0.49) | -2.11  (-2.27 to -1.96) | -2.49  (-2.54 to -2.44) | -3.5  (-3.67 to -3.33) | | | -1.93  (-2.01 to -1.85) | -3.8  (-3.98 to -3.63) | -3.96  (-4.05 to -3.88) | |
| **SDI region** | | | | | | | | | | | |
| High SDI | -4.12  (-4.3 to -3.95) | -4.1  (-4.21 to -3.99) | -4.8  (-4.97 to -4.63) | -2.46  (-2.83 to -2.1) | -5.69  (-5.87 to -5.52) | | | -5.71  (-5.83 to -5.6) | -6.31  (-6.48 to -6.14) | -4.05  (-4.41 to -3.68) | |
| High-middle SDI | -3.74  (-4.28 to -3.2) | 0.23  (-0.15 to 0.6) | -3.87  (-4.69 to -3.05) | -5.96  (-6.41 to -5.51) | -5.16  (-5.66 to -4.66) | | | -1.29  (-1.66 to -0.92) | -5.47  (-6.22 to -4.71) | -7.04  (-7.53 to -6.54) | |
| Middle SDI | -2.35  (-2.5 to -2.2) | -1.82  (-1.96 to -1.67) | -2.04  (-2.33 to -1.75) | -2.8  (-3.06 to -2.54) | -4.26  (-4.42 to -4.1) | | | -3.46  (-3.61 to -3.32) | -4.12  (-4.43 to -3.81) | -4.63  (-4.93 to -4.33) | |
| Low-middle SDI | -1.92  (-2.05 to -1.78) | -0.5  (-0.69 to -0.31) | -2.13  (-2.32 to -1.95) | -2.34  (-2.44 to -2.25) | -3.84  (-4 to -3.69) | | | -2.2  (-2.43 to -1.98) | -4.14  (-4.34 to -3.94) | -4.27  (-4.35 to -4.19) | |
| Low SDI | -1.39  (-1.56 to -1.21) | 0.3  (0.12 to 0.48) | -1.8  (-1.97 to -1.63) | -2.1  (-2.31 to -1.9) | -3.54  (-3.72 to -3.36) | | | -1.63  (-1.83 to -1.42) | -4.1  (-4.28 to -3.92) | -4.04  (-4.16 to -3.91) | |
| **GBD region** | | | | | | | | | | | |
| Andean Latin America | -5.04  (-5.47 to -4.61) | -5.84  (-6.74 to -4.93) | -5.77  (-6.22 to -5.31) | -1.64  (-2.31 to -0.97) | -6.99  (-7.37 to -6.6) | | | -7.67  (-8.58 to -6.75) | -7.54  (-7.97 to -7.12) | -3.99  (-4.65 to -3.32) | |
| Australasia | -2.41  (-2.72 to -2.1) | -3.06  (-4.05 to -2.07) | -1.79  (-2.54 to -1.03) | -0.08  (-1.04 to 0.89) | -4.5  (-4.77 to -4.23) | | | -5.06  (-6 to -4.11) | -3.86  (-4.56 to -3.16) | -2.57  (-3.46 to -1.68) | |
| Caribbean | -1.76  (-2.02 to -1.5) | -4.04  (-4.36 to -3.73) | -1.09  (-1.35 to -0.82) | -0.73  (-1.21 to -0.25) | -2.71  (-2.98 to -2.44) | | | -5.1  (-5.41 to -4.78) | -1.99  (-2.27 to -1.71) | -1.63  (-2.07 to -1.19) | |
| Central Asia | -3.17  (-4.1 to -2.23) | 8.05  (6.56 to 9.56) | -5.34  (-5.9 to -4.77) | -6.3  (-6.65 to -5.95) | -4.53  (-5.56 to -3.49) | | | 7.82  (6.24 to 9.43) | -6.97  (-7.65 to -6.28) | -7.81  (-8.16 to -7.47) | |
| Central Europe | -4.9  (-5.27 to -4.53) | -0.28  (-1.7 to 1.16) | -5.47  (-5.9 to -5.03) | -5.28  (-5.91 to -4.64) | -5.42  (-5.79 to -5.05) | | | -0.8  (-2.19 to 0.61) | -6.15  (-6.57 to -5.72) | -5.55  (-6.18 to -4.91) | |
| Central Latin America | -3.24  (-3.69 to -2.79) | -4.96  (-5.35 to -4.58) | -3.79  (-4.26 to -3.32) | -0.06  (-0.35 to 0.24) | -5.51  (-5.97 to -5.06) | | | -7.24  (-7.53 to -6.95) | -6.21  (-6.68 to -5.73) | -2.2  (-2.55 to -1.85) | |
| Central Sub-Saharan Africa | -0.17  (-0.54 to 0.21) | 1.8  (1.65 to 1.95) | -0.05  (-0.47 to 0.37) | -2.81  (-3.14 to -2.49) | -2.29  (-2.61 to -1.97) | | | -0.57  (-0.71 to -0.42) | -2.22  (-2.63 to -1.81) | -4.4  (-4.58 to -4.21) | |
| East Asia | -5.05  (-5.21 to -4.88) | -5.18  (-5.4 to -4.95) | -6.21  (-6.86 to -5.56) | -3.49  (-3.74 to -3.25) | -6.9  (-7.1 to -6.71) | | | -6.25  (-6.45 to -6.05) | -8.4  (-9.11 to -7.68) | -5.44  (-5.76 to -5.12) | |
| Eastern Europe | -2.54  (-4.03 to -1.03) | 9.24  (7.17 to 11.36) | -1.95  (-3.5 to -0.37) | -10.67  (-11.24 to -10.09) | -2.73  (-4.2 to -1.23) | | | 9.42  (7.33 to 11.55) | -2.33  (-3.8 to -0.83) | -10.68  (-11.32 to -10.03) | |
| Eastern Sub-Saharan Africa | -1.71  (-1.81 to -1.61) | -0.41  (-0.74 to -0.08) | -1.94  (-2.03 to -1.84) | -1.89  (-2 to -1.77) | -3.75  (-3.89 to -3.61) | | | -2  (-2.32 to -1.68) | -4.13  (-4.23 to -4.03) | -4  (-4.07 to -3.92) | |
| High-income Asia Pacific | -4.56  (-4.79 to -4.32) | -5.43  (-5.85 to -5.01) | -5.73  (-5.97 to -5.5) | -2.6  (-3.19 to -2.01) | -7.18  (-7.38 to -6.97) | | | -7.84  (-8.2 to -7.48) | -8.29  (-8.57 to -8.02) | -5.42  (-6.04 to -4.81) | |
| High-income North America | -3.73  (-4.29 to -3.16) | -5.97  (-6.55 to -5.38) | -4.99  (-5.29 to -4.68) | 1.11  (0.41 to 1.82) | -5.13  (-5.68 to -4.57) | | | -7.43  (-8 to -6.85) | -6.26  (-6.51 to -6.01) | -0.34  (-1.09 to 0.42) | |
| North Africa and Middle East | -2.36  (-2.49 to -2.23) | -1.06  (-1.22 to -0.89) | -2.55  (-2.87 to -2.23) | -2.48  (-2.55 to -2.4) | -4.68  (-4.83 to -4.54) | | | -3.13  (-3.3 to -2.95) | -5.12  (-5.39 to -4.85) | -4.48  (-4.6 to -4.35) | |
| Oceania | 1.02  (0.91 to 1.13) | 0.91  (0.63 to 1.21) | 1.62  (1.27 to 1.98) | 0.13  (-0.13 to 0.39) | -1.75  (-1.83 to -1.66) | | | -1.74  (-1.93 to -1.55) | -1.26  (-1.53 to -0.98) | -2.42  (-2.67 to -2.18) | |
| South Asia | -2.08  (-2.22 to -1.93) | -0.5  (-0.74 to -0.27) | -2.37  (-2.62 to -2.12) | -2.35  (-2.55 to -2.15) | -4.24  (-4.39 to -4.08) | | | -2.43  (-2.71 to -2.16) | -4.68  (-4.94 to -4.42) | -4.42  (-4.57 to -4.26) | |
| Southeast Asia | -2.06  (-2.2 to -1.91) | -0.98  (-1.09 to -0.87) | -2.15  (-2.31 to -1.99) | -2.5  (-2.64 to -2.36) | -3.93  (-4.09 to -3.76) | | -2.78  (-2.88 to -2.67) | | -3.91  (-4.08 to -3.73) | | -4.59  (-4.71 to -4.46) |
| Southern Latin America | -2.97  (-3.31 to -2.63) | -4.8  (-5.44 to -4.17) | -3.36  (-3.8 to -2.91) | -0.61  (-0.9 to -0.32) | -4.48  (-4.8 to -4.15) | | -6.12  (-6.76 to -5.48) | | -4.93  (-5.38 to -4.48) | | -2.14  (-2.43 to -1.85) |
| Southern Sub-Saharan Africa | 0.64  (-0.19 to 1.48) | 1.82  (0.74 to 2.9) | 3.08  (1.32 to 4.87) | -3.51  (-4.27 to -2.75) | -0.97  (-1.75 to -0.18) | | 0.43  (-0.63 to 1.5) | | 1.5  (0.01 to 3.02) | | -5.11  (-5.79 to -4.42) |
| Tropical Latin America | -2.34  (-2.51 to -2.18) | -1.25  (-1.6 to -0.9) | -3.2  (-3.57 to -2.84) | -1.13  (-1.22 to -1.03) | -4.45  (-4.63 to -4.27) | | -3.32  (-3.7 to -2.93) | | -5.42  (-5.75 to -5.09) | | -3.03  (-3.16 to -2.89) |
| Western Europe | -4.6  (-4.76 to -4.43) | -3.68  (-4.02 to -3.34) | -4.89  (-5.34 to -4.45) | -3.4  (-3.78 to -3.02) | -5.81  (-5.98 to -5.64) | | -4.99  (-5.27 to -4.71) | | -6.22  (-6.64 to -5.8) | | -4.5  (-4.86 to -4.14) |
| Western Sub-Saharan Africa | -0.73  (-0.99 to -0.47) | 1.53  (1.39 to 1.67) | -1.38  (-1.73 to -1.03) | -1.98  (-2.56 to -1.38) | -3.17  (-3.41 to -2.94) | | -0.93  (-1.12 to -0.75) | | -3.83  (-4.12 to -3.53) | | -4.18  (-4.61 to -3.75) |

Abbreviations: ASR, age-standardized rate; SDI, socio-demographic index

2.1 Supplementary Table S2-1: Change in disability-adjusted life-years of tuberculosis decomposed by three population-level determinants from 1990 to 1999 at the global and regional levels.

| **Location** | **Overall difference** | **Change due to population-level determinants**  **(% contribution to the total change)** | | |
| --- | --- | --- | --- | --- |
|  |  | **Population aging** | **Population growth** | **Epidemiological change** |
| **Global** | -4356430.14 | 399643.26 (-9.17%) | 9823631.16 (-225.5%) | -14579704.56 (334.67%) |
| **SDI region** | | | | |
| High SDI | -306653.74 | 51575.77 (-16.82%) | 53039.57 (-17.3%) | -411269.09 (134.12%) |
| High-middle SDI | 57377.23 | 157347.68 (274.23%) | 303713.45 (529.33%) | -403683.91 (-703.56%) |
| Middle SDI | -2878791.18 | 733979.84 (-25.5%) | 2100685.99 (-72.97%) | -5713457 (198.47%) |
| Low-middle SDI | -1884261.2 | 534448.04 (-28.36%) | 5519421.57 (-292.92%) | -7938130.82 (421.29%) |
| Low SDI | 658844.04 | -400729.66 (-60.82%) | 5622231.33 (853.35%) | -4562657.63 (-692.52%) |
| **GBD region** | | | | |
| Andean Latin America | -301676.6 | 12806.81 (-4.25%) | 104369.41 (-34.6%) | -418852.82 (138.84%) |
| Australasia | -865.66 | 271.65 (-31.38%) | 341.4 (-39.44%) | -1478.71 (170.82%) |
| Caribbean | -68119.44 | -1919.47 (2.82%) | 22096.72 (-32.44%) | -88296.7 (129.62%) |
| Central Asia | 325930.25 | -14964.95 (-4.59%) | 36667.85 (11.25%) | 304227.35 (93.34%) |
| Central Europe | -16468.27 | 13000.78 (-78.94%) | -4103.75 (24.92%) | -25365.31 (154.03%) |
| Central Latin America | -217954.2 | 30749.81 (-14.11%) | 86591.8 (-39.73%) | -335295.81 (153.84%) |
| Central Sub-Saharan Africa | 699337.55 | -74574.36 (-10.66%) | 1053718.02 (150.67%) | -279806.11 (-40.01%) |
| East Asia | -2833486.6 | 214923.12 (-7.59%) | 421867.56 (-14.89%) | -3470277.27 (122.47%) |
| Eastern Europe | 740962.75 | 24196.5 (3.27%) | -21887.58 (-2.95%) | 738653.83 (99.69%) |
| Eastern Sub-Saharan Africa | -414776.35 | -217146.14 (52.35%) | 2742640.45 (-661.23%) | -2940270.66 (708.88%) |
| High-income Asia Pacific | -150323.6 | 54495.08 (-36.25%) | 11517.84 (-7.66%) | -216336.53 (143.91%) |
| High-income North America | -27931.52 | 2158.2 (-7.73%) | 5338.99 (-19.11%) | -35428.71 (126.84%) |
| North Africa and Middle East | -185186.57 | -12215.44 (6.6%) | 327447.11 (-176.82%) | -500418.25 (270.22%) |
| Oceania | 9444.04 | 899.45 (9.52%) | 23917.85 (253.26%) | -15373.26 (-162.78%) |
| South Asia | -1922701.31 | 723403.9 (-37.62%) | 5909494.72 (-307.35%) | -8555599.93 (444.98%) |
| Southeast Asia | -992527.09 | 463451.73 (-46.69%) | 1532622.92 (-154.42%) | -2988601.75 (301.11%) |
| Southern Latin America | -41667.94 | 2289.07 (-5.49%) | 8644.88 (-20.75%) | -52601.89 (126.24%) |
| Southern Sub-Saharan Africa | 289252.05 | 27972.97 (9.67%) | 309133.98 (106.87%) | -47854.9 (-16.54%) |
| Tropical Latin America | -47863.06 | 23146.04 (-48.36%) | 61218 (-127.9%) | -132227.1 (276.26%) |
| Western Europe | -65841.79 | 13119.54 (-19.93%) | 5779.98 (-8.78%) | -84741.32 (128.7%) |
| Western Sub-Saharan Africa | 866033.24 | -147999.03 (-17.09%) | 1741564.36 (201.1%) | -727532.1 (-84.01%) |

Abbreviation: SDI, sociodemographic index

2.2 Supplementary Table S2-2: Change in disability-adjusted life-years of tuberculosis decomposed by three population-level determinants from 2000 to 2009 at the global and regional levels.

| **Location** | **Overall difference** | **Change due to population-level determinants**  **(% contribution to the total change)** | | |
| --- | --- | --- | --- | --- |
|  |  | **Population aging** | **Population growth** | **Epidemiological change** |
| **Global** | -13936729.23 | 2537360.85 (-18.21%) | 8419231.18 (-60.41%) | -24893321.26 (178.62%) |
| **SDI region** | | | | |
| High SDI | -225206 | 39111.34 (-17.37%) | 39573.33 (-17.57%) | -303890.84 (134.94%) |
| High-middle SDI | -1254737 | 259382.12 (-20.67%) | 205786.22 (-16.4%) | -1719904.94 (137.07%) |
| Middle SDI | -2863794 | 1215772.79 (-42.45%) | 1577288.06 (-55.08%) | -5656854.65 (197.53%) |
| Low-middle SDI | -5932950 | 1299405.33 (-21.9%) | 4465715.2 (-75.27%) | -11698070.69 (197.17%) |
| Low SDI | -3653739 | -144051.93 (3.94%) | 5565346.27 (-152.32%) | -9075033.49 (248.38%) |
| **GBD region** | | | | |
| Andean Latin America | -153315.86 | 15048.81 (-9.82%) | 42902.06 (-27.98%) | -211266.74 (137.8%) |
| Australasia | -397.47 | 185.74 (-46.73%) | 273.25 (-68.75%) | -856.46 (215.48%) |
| Caribbean | -17689.39 | -74.18 (0.42%) | 11630.07 (-65.75%) | -29245.28 (165.33%) |
| Central Asia | -289842.48 | 43071.43 (-14.86%) | 50205.57 (-17.32%) | -383119.48 (132.18%) |
| Central Europe | -77969.83 | 11776.77 (-15.1%) | -4199.82 (5.39%) | -85546.78 (109.72%) |
| Central Latin America | -95091.48 | 27080.18 (-28.48%) | 37001.87 (-38.91%) | -159173.53 (167.39%) |
| Central Sub-Saharan Africa | 30696.28 | -9309.76 (-30.33%) | 1271859.01 (4143.37%) | -1231852.98 (-4013.04%) |
| East Asia | -2049325.73 | 461123.35 (-22.5%) | 210754.66 (-10.28%) | -2721203.75 (132.79%) |
| Eastern Europe | -282517.6 | 105612.28 (-37.38%) | -53095.53 (18.79%) | -335034.35 (118.59%) |
| Eastern Sub-Saharan Africa | -1838976.79 | -60767.94 (3.3%) | 2433499.55 (-132.33%) | -4211708.4 (229.02%) |
| High-income Asia Pacific | -94054.28 | 38311.47 (-40.73%) | 4346.8 (-4.62%) | -136712.55 (145.35%) |
| High-income North America | -13565.67 | 1724.72 (-12.71%) | 2696.96 (-19.88%) | -17987.35 (132.59%) |
| North Africa and Middle East | -328908.88 | 57665.92 (-17.53%) | 261470.89 (-79.5%) | -648045.69 (197.03%) |
| Oceania | 13688.23 | 2710.7 (19.8%) | 27226.27 (198.9%) | -16248.75 (-118.71%) |
| South Asia | -6585890.29 | 1602337.22 (-24.33%) | 4857322.19 (-73.75%) | -13045549.7 (198.08%) |
| Southeast Asia | -1810982.5 | 619162.01 (-34.19%) | 1106503.75 (-61.1%) | -3536648.26 (195.29%) |
| Southern Latin America | -14673.96 | 2579.12 (-17.58%) | 4866.62 (-33.17%) | -22119.69 (150.74%) |
| Southern Sub-Saharan Africa | 620066.58 | 115627.57 (18.65%) | 230128.24 (37.11%) | 274310.78 (44.24%) |
| Tropical Latin America | -89015.85 | 28077.29 (-31.54%) | 36955.04 (-41.52%) | -154048.18 (173.06%) |
| Western Europe | -48095.9 | 9116.07 (-18.95%) | 5870.69 (-12.21%) | -63082.66 (131.16%) |
| Western Sub-Saharan Africa | -810866.36 | -58037.09 (7.16%) | 1898390.62 (-234.12%) | -2651219.9 (326.96%) |

Abbreviation: SDI, sociodemographic index

2.3 Supplementary Table S2-3: Change in disability-adjusted life-years of tuberculosis decomposed by three population-level determinants from 2010 to 2021 at the global and regional levels.

| **Location** | **Overall difference** | **Change due to population-level determinants**  **(% contribution to the total change)** | | |
| --- | --- | --- | --- | --- |
|  |  | **Population aging** | **Population growth** | **Epidemiological change** |
| **Global** | -14861550.44 | 2008446.38 (-13.51%) | 7000852.02 (-47.11%) | -23870848.84 (160.62%) |
| **SDI region** | | | | |
| High SDI | -92235.4 | 35670.29 (-38.67%) | 22363.94 (-24.25%) | -150269.57 (162.92%) |
| High-middle SDI | -1295579 | 110104.92 (-8.5%) | 124039.61 (-9.57%) | -1529723.89 (118.07%) |
| Middle SDI | -3432973 | 1072175.58 (-31.23%) | 1261188.41 (-36.74%) | -5766336.73 (167.97%) |
| Low-middle SDI | -5916202 | 1737741.07 (-29.37%) | 3614357.86 (-61.09%) | -11268300.84 (190.47%) |
| Low SDI | -4118450 | 131475.45 (-3.19%) | 5000113.53 (-121.41%) | -9250039.06 (224.6%) |
| **GBD region** | | | | |
| Andean Latin America | -46952.93 | 11822.18 (-25.18%) | 40276.95 (-85.78%) | -99052.05 (210.96%) |
| Australasia | -123.32 | 202.54 (-164.25%) | 350.18 (-283.97%) | -676.05 (548.22%) |
| Caribbean | -7765.38 | 1988.33 (-25.61%) | 9520.23 (-122.6%) | -19273.94 (248.2%) |
| Central Asia | -202951.56 | 13726.58 (-6.76%) | 51808.61 (-25.53%) | -268486.75 (132.29%) |
| Central Europe | -57655.3 | 5574.3 (-9.67%) | -2999.31 (5.2%) | -60230.28 (104.47%) |
| Central Latin America | -7285.35 | 29090.19 (-399.3%) | 23959.87 (-328.88%) | -60335.41 (828.17%) |
| Central Sub-Saharan Africa | -1136425.35 | -42492.07 (3.74%) | 1274891.92 (-112.18%) | -2368825.21 (208.45%) |
| East Asia | -864991.2 | 328895.18 (-38.02%) | 136982.92 (-15.84%) | -1330869.3 (153.86%) |
| Eastern Europe | -759289.33 | -18391.44 (2.42%) | -16186.34 (2.13%) | -724711.55 (95.45%) |
| Eastern Sub-Saharan Africa | -1633104.07 | 215268.14 (-13.18%) | 2154353.02 (-131.92%) | -4002725.23 (245.1%) |
| High-income Asia Pacific | -32103.64 | 29462.32 (-91.77%) | 441.29 (-1.37%) | -62007.26 (193.15%) |
| High-income North America | 1853.18 | 2317.58 (125.06%) | 1877.12 (101.29%) | -2341.51 (-126.35%) |
| North Africa and Middle East | -283204.29 | 49992.4 (-17.65%) | 192845.46 (-68.09%) | -526042.15 (185.75%) |
| Oceania | 4962.27 | 5966.01 (120.23%) | 36857.52 (742.76%) | -37861.26 (-762.98%) |
| South Asia | -5791399.11 | 2161575.38 (-37.32%) | 3423891.23 (-59.12%) | -11376865.72 (196.44%) |
| Southeast Asia | -1970963.62 | 827729.06 (-42%) | 890588.33 (-45.19%) | -3689281.01 (187.18%) |
| Southern Latin America | -2900.57 | 2944.94 (-101.53%) | 4269.67 (-147.2%) | -10115.18 (348.73%) |
| Southern Sub-Saharan Africa | -797666.93 | 128143.59 (-16.06%) | 276646.75 (-34.68%) | -1202457.27 (150.75%) |
| Tropical Latin America | -31807.29 | 24378.68 (-76.64%) | 27362.42 (-86.03%) | -83548.4 (262.67%) |
| Western Europe | -26690.77 | 7140.94 (-26.75%) | 2554.34 (-9.57%) | -36386.04 (136.32%) |
| Western Sub-Saharan Africa | -1215085.87 | -145076.89 (11.94%) | 1847813.14 (-152.07%) | -2917822.11 (240.13%) |

Abbreviation: SDI, sociodemographic index

3. Supplementary Table S3: Change in disability-adjusted life-years of tuberculosis decomposed by three population-level determinants from 1990 to 2021 at the national level.

| **Location** | **Overall difference** | **Change due to population-level determinants**  **(% contribution to the total change)** | | |
| --- | --- | --- | --- | --- |
|  |  | **Population aging** | **Population growth** | **Epidemiological change** |
| Afghanistan | -96856.6 | -73992.94 (76.39%) | 697857.84 (-720.51%) | -720721.51 (744.11%) |
| Albania | -3544.1 | 1161.71 (-32.78%) | -606.29 (17.11%) | -4099.52 (115.67%) |
| Algeria | -80229.4 | 18357.74 (-22.88%) | 60672.46 (-75.62%) | -159259.6 (198.51%) |
| American Samoa | -20.12 | 19.97 (-99.25%) | 1.54 (-7.64%) | -41.63 (206.9%) |
| Andorra | -0.86 | 1.28 (-149.56%) | 1.5 (-174.63%) | -3.64 (424.2%) |
| Angola | -189019 | -44291.44 (23.43%) | 1077481.95 (-570.04%) | -1222209.48 (646.61%) |
| Antigua and Barbuda | -23.2 | 4.76 (-20.52%) | 12.15 (-52.37%) | -40.11 (172.89%) |
| Argentina | -37200.1 | 4615.9 (-12.41%) | 15688.97 (-42.17%) | -57504.95 (154.58%) |
| Armenia | -4045.22 | 507.61 (-12.55%) | -488.69 (12.08%) | -4064.14 (100.47%) |
| Australia | -936.14 | 763.02 (-81.51%) | 1048.05 (-111.95%) | -2747.2 (293.46%) |
| Austria | -3421.31 | 650.78 (-19.02%) | 455.97 (-13.33%) | -4528.05 (132.35%) |
| Azerbaijan | -26565.8 | 3054.56 (-11.5%) | 14416.34 (-54.27%) | -44036.67 (165.76%) |
| Bahamas | -262.57 | 95.75 (-36.47%) | 177.11 (-67.45%) | -535.44 (203.92%) |
| Bahrain | -170.08 | 254.51 (-149.64%) | 915.63 (-538.34%) | -1340.22 (787.98%) |
| Bangladesh | -3278285 | 441287.23 (-13.46%) | 1310074.11 (-39.96%) | -5029646.39 (153.42%) |
| Barbados | -90.5 | 17.03 (-18.81%) | 17.71 (-19.57%) | -125.24 (138.38%) |
| Belarus | -8115.8 | 1871.21 (-23.06%) | -1504.09 (18.53%) | -8482.92 (104.52%) |
| Belgium | -2870.41 | 570.69 (-19.88%) | 407.75 (-14.21%) | -3848.85 (134.09%) |
| Belize | -223.4 | 89.92 (-40.25%) | 612.8 (-274.31%) | -926.11 (414.56%) |
| Benin | -30020.1 | -5818.23 (19.38%) | 125912.28 (-419.43%) | -150114.12 (500.05%) |
| Bermuda | -9.84 | -0.07 (0.67%) | 0.65 (-6.6%) | -10.42 (105.94%) |
| Bhutan | -7952.77 | 2978.76 (-37.46%) | 1586.93 (-19.95%) | -12518.46 (157.41%) |
| Bolivia (Plurinational State of) | -151465 | 14286.59 (-9.43%) | 102745.13 (-67.83%) | -268496.56 (177.27%) |
| Bosnia and Herzegovina | -10307.5 | 3996.98 (-38.78%) | -2575.81 (24.99%) | -11728.67 (113.79%) |
| Botswana | -10473.5 | 9467.3 (-90.39%) | 28223.39 (-269.47%) | -48164.19 (459.87%) |
| Brazil | -198473 | 80465.31 (-40.54%) | 142453.83 (-71.77%) | -421391.93 (212.32%) |
| Brunei Darussalam | -328.65 | 337.44 (-102.68%) | 452.42 (-137.66%) | -1118.52 (340.33%) |
| Bulgaria | -7469.88 | 1175.51 (-15.74%) | -1557.85 (20.86%) | -7087.55 (94.88%) |
| Burkina Faso | 16371.95 | -10964.56 (-66.97%) | 248825.4 (1519.83%) | -221488.89 (-1352.86%) |
| Burundi | -121831 | -17553.07 (14.41%) | 381376.84 (-313.04%) | -485654.9 (398.63%) |
| Cabo Verde | -1883.64 | 466.53 (-24.77%) | 1481.51 (-78.65%) | -3831.68 (203.42%) |
| Cambodia | -248938 | 75454.14 (-30.31%) | 220380.32 (-88.53%) | -544772.44 (218.84%) |
| Cameroon | 21702.89 | -11690.72 (-53.87%) | 286871.25 (1321.81%) | -253477.64 (-1167.94%) |
| Canada | -3556.17 | 1584.55 (-44.56%) | 1638.19 (-46.07%) | -6778.91 (190.62%) |
| Central African Republic | 76837.54 | -12948.46 (-16.85%) | 249294.5 (324.44%) | -159508.5 (-207.59%) |
| Chad | 103486.5 | -29129.76 (-28.15%) | 321806.76 (310.96%) | -189190.5 (-182.82%) |
| Chile | -26350 | 9231.77 (-35.04%) | 10728.99 (-40.72%) | -46310.73 (175.75%) |
| China | -5950220 | 1624403.24 (-27.3%) | 965715.25 (-16.23%) | -8540338.44 (143.53%) |
| Colombia | -35123.6 | 17847.49 (-50.81%) | 24565.99 (-69.94%) | -77537.03 (220.76%) |
| Comoros | -6537.51 | 2810.08 (-42.98%) | 7942.94 (-121.5%) | -17290.53 (264.48%) |
| Congo | -16759.1 | 10515.14 (-62.74%) | 87392.94 (-521.47%) | -114667.13 (684.21%) |
| Cook Islands | -14.98 | 9.52 (-63.54%) | -1.27 (8.51%) | -23.22 (155.03%) |
| Costa Rica | -2087.39 | 1407.22 (-67.42%) | 1541.63 (-73.85%) | -5036.24 (241.27%) |
| Coted'Ivoire | -29750.1 | 14187.61 (-47.69%) | 226629.39 (-761.78%) | -270567.05 (909.47%) |
| Croatia | -10322.4 | 1983.1 (-19.21%) | -971.53 (9.41%) | -11333.96 (109.8%) |
| Cuba | -3051.42 | 604.8 (-19.82%) | 113.02 (-3.7%) | -3769.24 (123.52%) |
| Cyprus | -208.47 | 141.21 (-67.74%) | 181.84 (-87.22%) | -531.52 (254.96%) |
| Czechia | -5032.24 | 893.29 (-17.75%) | 122.93 (-2.44%) | -6048.46 (120.19%) |
| Democratic People's Republic of Korea | -59950.4 | 91309.19 (-152.31%) | 99846.42 (-166.55%) | -251106.05 (418.86%) |
| Democratic Republic of the Congo | -145502 | -134916.41 (92.72%) | 2244591.27 (-1542.66%) | -2255176.56 (1549.93%) |
| Denmark | -1153.65 | 195.54 (-16.95%) | 144.11 (-12.49%) | -1493.3 (129.44%) |
| Djibouti | 4314.55 | 3173.99 (73.56%) | 18829.64 (436.42%) | -17689.08 (-409.99%) |
| Dominica | -111.71 | 35.76 (-32.02%) | -10.78 (9.65%) | -136.69 (122.37%) |
| Dominican Republic | -32486 | 4370.08 (-13.45%) | 21590.19 (-66.46%) | -58446.29 (179.91%) |
| Ecuador | -78377.8 | 11849.45 (-15.12%) | 46189.94 (-58.93%) | -136417.15 (174.05%) |
| Egypt | -63315.4 | 6383.75 (-10.08%) | 57031.74 (-90.08%) | -126730.93 (200.16%) |
| El Salvador | -22814.8 | 4095.99 (-17.95%) | 4013.98 (-17.59%) | -30924.73 (135.55%) |
| Equatorial Guinea | -23400.2 | -5436.5 (23.23%) | 43991.41 (-188%) | -61955.11 (264.76%) |
| Eritrea | -21558.4 | 20482.52 (-95.01%) | 162013.09 (-751.51%) | -204053.98 (946.52%) |
| Estonia | -2504.96 | 136.85 (-5.46%) | -332.92 (13.29%) | -2308.88 (92.17%) |
| Eswatini | 5135.99 | 3534.72 (68.82%) | 10407.86 (202.65%) | -8806.59 (-171.47%) |
| Ethiopia | -3287075 | -102926.79 (3.13%) | 2928145.56 (-89.08%) | -6112293.46 (185.95%) |
| Fiji | -685.39 | 596.05 (-86.96%) | 467.94 (-68.27%) | -1749.38 (255.24%) |
| Finland | -2966.76 | 924.66 (-31.17%) | 269.9 (-9.1%) | -4161.32 (140.26%) |
| France | -37939.4 | 11827.87 (-31.18%) | 4916.82 (-12.96%) | -54684.09 (144.14%) |
| Gabon | -7374.08 | 1051.66 (-14.26%) | 14955.37 (-202.81%) | -23381.11 (317.07%) |
| Gambia | 2214.44 | 767.63 (34.66%) | 23111.12 (1043.66%) | -21664.31 (-978.32%) |
| Georgia | -15583.6 | 802.18 (-5.15%) | -4703.44 (30.18%) | -11682.32 (74.97%) |
| Germany | -37192.7 | 7134.43 (-19.18%) | 2026.65 (-5.45%) | -46353.73 (124.63%) |
| Ghana | -32204.5 | 30484.15 (-94.66%) | 318924.22 (-990.31%) | -381612.83 (1184.97%) |
| Greece | -3418.6 | 1645.14 (-48.12%) | -84.21 (2.46%) | -4979.53 (145.66%) |
| Greenland | -75.75 | 70.43 (-92.98%) | 1.14 (-1.51%) | -147.33 (194.49%) |
| Grenada | -49.93 | 9.64 (-19.3%) | 7.87 (-15.77%) | -67.44 (135.06%) |
| Guam | 21.5 | 138.06 (642.12%) | 50.74 (235.99%) | -167.3 (-778.1%) |
| Guatemala | -64007.1 | 9696.45 (-15.15%) | 41690.2 (-65.13%) | -115393.72 (180.28%) |
| Guinea | -52665.7 | -22512.29 (42.75%) | 151175.26 (-287.05%) | -181328.69 (344.3%) |
| Guinea-Bissau | -16230.9 | -820.33 (5.05%) | 36677.56 (-225.97%) | -52088.1 (320.92%) |
| Guyana | -3433.28 | 820.2 (-23.89%) | -75.71 (2.21%) | -4177.76 (121.68%) |
| Haiti | -54359 | -2599.24 (4.78%) | 86394.39 (-158.93%) | -138154.17 (254.15%) |
| Honduras | -12605.5 | 6842.48 (-54.28%) | 27375.37 (-217.17%) | -46823.38 (371.45%) |
| Hungary | -17188.4 | 2321.94 (-13.51%) | -865.52 (5.04%) | -18644.79 (108.47%) |
| Iceland | -44.68 | 18.9 (-42.3%) | 20.57 (-46.03%) | -84.15 (188.33%) |
| India | -1.1E+07 | 4590807.63 (-40.33%) | 12057013.59 (-105.93%) | -28029988.19 (246.26%) |
| Indonesia | -2390836 | 919791.93 (-38.47%) | 1989534.6 (-83.22%) | -5300162.67 (221.69%) |
| Iran (Islamic Republic of) | -35109.2 | 9184.98 (-26.16%) | 20454.85 (-58.26%) | -64749.04 (184.42%) |
| Iraq | -34966.7 | 9366.97 (-26.79%) | 53999.53 (-154.43%) | -98333.15 (281.22%) |
| Ireland | -1304.3 | 399.51 (-30.63%) | 436.73 (-33.48%) | -2140.54 (164.11%) |
| Israel | -720.04 | 282.38 (-39.22%) | 842.93 (-117.07%) | -1845.36 (256.28%) |
| Italy | -16642.1 | 4433.12 (-26.64%) | 793.34 (-4.77%) | -21868.51 (131.41%) |
| Jamaica | -662.21 | 192.67 (-29.1%) | 151.16 (-22.83%) | -1006.05 (151.92%) |
| Japan | -70854.1 | 73432.13 (-103.64%) | 1496.36 (-2.11%) | -145782.61 (205.75%) |
| Jordan | -94.5 | 703.9 (-744.84%) | 3104.48 (-3285.07%) | -3902.88 (4129.91%) |
| Kazakhstan | -63335.1 | 5545.46 (-8.76%) | 9128.58 (-14.41%) | -78009.11 (123.17%) |
| Kenya | 250473.5 | 83238.85 (33.23%) | 627379.02 (250.48%) | -460144.36 (-183.71%) |
| Kiribati | -142.21 | 266.73 (-187.56%) | 1824.68 (-1283.09%) | -2233.62 (1570.65%) |
| Kuwait | 116.84 | 554.16 (474.28%) | 1917.95 (1641.49%) | -2355.27 (-2015.77%) |
| Kyrgyzstan | -6545.44 | 1464.97 (-22.38%) | 8953.28 (-136.79%) | -16963.69 (259.17%) |
| Lao People's Democratic Republic | -125722 | 11012.41 (-8.76%) | 92347.71 (-73.45%) | -229081.93 (182.21%) |
| Latvia | -4437.22 | 334.79 (-7.54%) | -1246.8 (28.1%) | -3525.2 (79.45%) |
| Lebanon | -2849.14 | 918.21 (-32.23%) | 2392.34 (-83.97%) | -6159.69 (216.19%) |
| Lesotho | 49494.01 | 5670.38 (11.46%) | 18087.04 (36.54%) | 25736.59 (52%) |
| Liberia | -40185.8 | -8538.85 (21.25%) | 54807.41 (-136.38%) | -86454.4 (215.14%) |
| Libya | -556.51 | 1794.18 (-322.4%) | 3383.67 (-608.02%) | -5734.36 (1030.42%) |
| Lithuania | -7507.61 | 947.8 (-12.62%) | -2095.09 (27.91%) | -6360.31 (84.72%) |
| Luxembourg | -66.74 | 8.61 (-12.91%) | 42.84 (-64.19%) | -118.2 (177.09%) |
| Madagascar | -88132.4 | -14147.89 (16.05%) | 551490.02 (-625.75%) | -625474.5 (709.7%) |
| Malawi | -278840 | -50535.84 (18.12%) | 348316.68 (-124.92%) | -576621.11 (206.79%) |
| Malaysia | -28270.6 | 33498.47 (-118.49%) | 58688.25 (-207.59%) | -120457.31 (426.09%) |
| Maldives | -3156.21 | 951.07 (-30.13%) | 3087.22 (-97.81%) | -7194.5 (227.95%) |
| Mali | -76055 | -27817.83 (36.58%) | 306625.06 (-403.16%) | -354862.22 (466.59%) |
| Malta | -37.27 | 23.67 (-63.51%) | 8.72 (-23.4%) | -69.66 (186.91%) |
| Marshall Islands | -29.36 | 477.65 (-1626.75%) | 301.2 (-1025.81%) | -808.21 (2752.56%) |
| Mauritania | -22455 | -263.57 (1.17%) | 27025.22 (-120.35%) | -49216.63 (219.18%) |
| Mauritius | -640.77 | 453.98 (-70.85%) | 159.37 (-24.87%) | -1254.12 (195.72%) |
| Mexico | -189742 | 77702.35 (-40.95%) | 99454.85 (-52.42%) | -366898.69 (193.37%) |
| Micronesia (Federated States of) | -469.18 | 207.67 (-44.26%) | -6.77 (1.44%) | -670.08 (142.82%) |
| Monaco | -16.78 | 1.48 (-8.84%) | 4.56 (-27.17%) | -22.83 (136.01%) |
| Mongolia | -20521.9 | 5399.4 (-26.31%) | 15453.5 (-75.3%) | -41374.78 (201.61%) |
| Montenegro | -191.64 | 71.55 (-37.34%) | -3.98 (2.08%) | -259.21 (135.26%) |
| Morocco | -163242 | 36558.02 (-22.39%) | 79052.12 (-48.43%) | -278852.07 (170.82%) |
| Mozambique | -212920 | -89101.98 (41.85%) | 921459.17 (-432.77%) | -1045277.05 (490.93%) |
| Myanmar | -1534911 | 192711.57 (-12.56%) | 514172.7 (-33.5%) | -2241795.44 (146.05%) |
| Namibia | -5279.87 | 5430.89 (-102.86%) | 27419.33 (-519.32%) | -38130.09 (722.18%) |
| Nauru | -59.19 | 9.35 (-15.8%) | 10 (-16.9%) | -78.54 (132.69%) |
| Nepal | -490623 | 99583.07 (-20.3%) | 271538.84 (-55.35%) | -861744.43 (175.64%) |
| Netherlands | -3128.38 | 1128.67 (-36.08%) | 498.56 (-15.94%) | -4755.61 (152.01%) |
| New Zealand | -799.77 | 176.84 (-22.11%) | 318.62 (-39.84%) | -1295.23 (161.95%) |
| Nicaragua | -16983.8 | 3303.24 (-19.45%) | 11386.95 (-67.05%) | -31674.03 (186.5%) |
| Niger | -98863.3 | -15832.72 (16.01%) | 572434.21 (-579.02%) | -655464.76 (663%) |
| Nigeria | -887267 | -284523.47 (32.07%) | 3199660.08 (-360.62%) | -3802403.56 (428.55%) |
| Niue | -5.69 | 1.42 (-25.05%) | -2.04 (35.88%) | -5.07 (89.17%) |
| North Macedonia | -3694.45 | 812.28 (-21.99%) | 280.13 (-7.58%) | -4786.86 (129.57%) |
| Northern Mariana Islands | -106.82 | 119.28 (-111.66%) | 16.39 (-15.34%) | -242.49 (227%) |
| Norway | -822.1 | 133.88 (-16.29%) | 293.51 (-35.7%) | -1249.5 (151.99%) |
| Oman | -1348.43 | 270.01 (-20.02%) | 1761.38 (-130.62%) | -3379.83 (250.65%) |
| Pakistan | -384303 | 59266.87 (-15.42%) | 2636040.43 (-685.93%) | -3079610.15 (801.35%) |
| Palau | -6.31 | 21.92 (-347.58%) | 11.42 (-181.09%) | -39.64 (628.67%) |
| Palestine | -1018.96 | 65.13 (-6.39%) | 1998.99 (-196.18%) | -3083.08 (302.57%) |
| Panama | -4932.31 | 1837.08 (-37.25%) | 5707.18 (-115.71%) | -12476.57 (252.96%) |
| Papua New Guinea | 35282.6 | 7215.37 (20.45%) | 101742.88 (288.37%) | -73675.65 (-208.82%) |
| Paraguay | -2650.23 | 1796.19 (-67.77%) | 7050.69 (-266.04%) | -11497.11 (433.81%) |
| Peru | -313276 | 30646.53 (-9.78%) | 159964 (-51.06%) | -503886.08 (160.84%) |
| Philippines | 68001.84 | 189365.44 (278.47%) | 654827.63 (962.96%) | -776191.23 (-1141.43%) |
| Poland | -47954.8 | 13704.11 (-28.58%) | 71.74 (-0.15%) | -61730.62 (128.73%) |
| Portugal | -9489.18 | 3624.5 (-38.2%) | 451.04 (-4.75%) | -13564.72 (142.95%) |
| Puerto Rico | -2867.6 | 672.1 (-23.44%) | -187.64 (6.54%) | -3352.06 (116.89%) |
| Qatar | -5.74 | 351.94 (-6128.45%) | 3831.77 (-66723.92%) | -4189.45 (72952.37%) |
| Republic of Korea | -219893 | 158088.31 (-71.89%) | 32652.12 (-14.85%) | -410632.99 (186.74%) |
| Republic of Moldova | -5022.51 | 1483.43 (-29.54%) | -1705.84 (33.96%) | -4800.1 (95.57%) |
| Romania | -51776 | 7333.4 (-14.16%) | -11210.09 (21.65%) | -47899.29 (92.51%) |
| Russian Federation | -226666 | 36418.16 (-16.07%) | -14343.4 (6.33%) | -248740.92 (109.74%) |
| Rwanda | -292355 | 22516.6 (-7.7%) | 222967.56 (-76.27%) | -537839.58 (183.97%) |
| Saint Kitts and Nevis | -66.16 | 20 (-30.23%) | 25.51 (-38.55%) | -111.66 (168.78%) |
| Saint Lucia | -216.76 | 116.73 (-53.85%) | 74.71 (-34.47%) | -408.2 (188.32%) |
| Saint Vincent and the Grenadines | -181.39 | 52.09 (-28.72%) | 6.97 (-3.84%) | -240.45 (132.56%) |
| Samoa | -205.47 | 111.15 (-54.09%) | 165.14 (-80.37%) | -481.75 (234.47%) |
| San Marino | -1.51 | 1.23 (-81.12%) | 1.15 (-76%) | -3.89 (257.11%) |
| Sao Tome and Principe | -589.65 | 18.41 (-3.12%) | 658.83 (-111.73%) | -1266.89 (214.85%) |
| Saudi Arabia | -32657.1 | 18807.38 (-57.59%) | 72766.72 (-222.82%) | -124231.16 (380.41%) |
| Senegal | -59843.1 | 6018.36 (-10.06%) | 122429.54 (-204.58%) | -188291 (314.64%) |
| Serbia | -10586.4 | 1873.14 (-17.69%) | -676.06 (6.39%) | -11783.5 (111.31%) |
| Seychelles | -64.33 | 59.37 (-92.28%) | 74.97 (-116.54%) | -198.67 (308.83%) |
| Sierra Leone | -37402.5 | -16240.94 (43.42%) | 116260.89 (-310.84%) | -137422.47 (367.42%) |
| Singapore | -2947.04 | 2423.53 (-82.24%) | 2665.04 (-90.43%) | -8035.61 (272.67%) |
| Slovakia | -3313.65 | 819.96 (-24.75%) | 80.19 (-2.42%) | -4213.8 (127.16%) |
| Slovenia | -1257.18 | 249.17 (-19.82%) | 44.22 (-3.52%) | -1550.57 (123.34%) |
| Solomon Islands | 803.76 | 492.56 (61.28%) | 2332.94 (290.25%) | -2021.75 (-251.54%) |
| Somalia | 401878.3 | -23651.94 (-5.89%) | 756188.29 (188.16%) | -330658.04 (-82.28%) |
| South Africa | -127084 | 130799.29 (-102.92%) | 492486.44 (-387.53%) | -750370.17 (590.45%) |
| South Sudan | 7776.22 | -5391 (-69.33%) | 143590.82 (1846.54%) | -130423.59 (-1677.21%) |
| Spain | -22523.8 | 5766.58 (-25.6%) | 3146.03 (-13.97%) | -31436.41 (139.57%) |
| Sri Lanka | -51817 | 23928.81 (-46.18%) | 18423.88 (-35.56%) | -94169.72 (181.74%) |
| Sudan | -137577 | -13391.76 (9.73%) | 126648.92 (-92.06%) | -250834.45 (182.32%) |
| Suriname | -315.87 | 100.12 (-31.7%) | 252.54 (-79.95%) | -668.52 (211.65%) |
| Sweden | -2326.87 | 390.05 (-16.76%) | 433.81 (-18.64%) | -3150.73 (135.41%) |
| Switzerland | -1982.47 | 375.5 (-18.94%) | 489.39 (-24.69%) | -2847.36 (143.63%) |
| Syrian Arab Republic | -10253.9 | 2072.13 (-20.21%) | 940.87 (-9.18%) | -13266.85 (129.38%) |
| Taiwan (Province of China) | -59864.4 | 41216.31 (-68.85%) | 9423.19 (-15.74%) | -110503.86 (184.59%) |
| Tajikistan | -20035.2 | 405.78 (-2.03%) | 35579.58 (-177.59%) | -56020.55 (279.61%) |
| Thailand | -245646 | 299906.68 (-122.09%) | 73455.4 (-29.9%) | -619008 (251.99%) |
| Timor-Leste | -14998.1 | 1883.12 (-12.56%) | 20160.51 (-134.42%) | -37041.76 (246.98%) |
| Togo | 6813.38 | 9266.12 (136%) | 77018.12 (1130.4%) | -79470.86 (-1166.39%) |
| Tokelau | -6.54 | 1.14 (-17.44%) | -0.94 (14.4%) | -6.74 (103.04%) |
| Tonga | -99.84 | 41.53 (-41.6%) | 17.84 (-17.87%) | -159.22 (159.47%) |
| Trinidad and Tobago | -860.92 | 348.45 (-40.47%) | 167.44 (-19.45%) | -1376.81 (159.92%) |
| Tunisia | -10536.4 | 3531.15 (-33.51%) | 4741.68 (-45%) | -18809.22 (178.52%) |
| Turkey | -176600 | 28329.92 (-16.04%) | 54225.68 (-30.71%) | -259155.93 (146.75%) |
| Turkmenistan | -5071.72 | 1846.03 (-36.4%) | 7390.14 (-145.71%) | -14307.89 (282.11%) |
| Tuvalu | -259.78 | 18.65 (-7.18%) | 80.7 (-31.06%) | -359.13 (138.24%) |
| Uganda | -37618.4 | -43752.36 (116.31%) | 599953.64 (-1594.84%) | -593819.68 (1578.54%) |
| Ukraine | -25260.1 | 12924.83 (-51.17%) | -22015.96 (87.16%) | -16168.95 (64.01%) |
| United Arab Emirates | 323.19 | 870.92 (269.48%) | 3991.5 (1235.05%) | -4539.24 (-1404.53%) |
| United Kingdom | -11566.8 | 1608.73 (-13.91%) | 2306.39 (-19.94%) | -15481.93 (133.85%) |
| United Republic of Tanzania | -338106 | -15847.1 (4.69%) | 804078.37 (-237.82%) | -1126337.25 (333.13%) |
| United States of America | -42182.8 | 8853.98 (-20.99%) | 13234.04 (-31.37%) | -64270.79 (152.36%) |
| United States Virgin Islands | -43.66 | 17.02 (-39%) | -9.67 (22.14%) | -51.02 (116.85%) |
| Uruguay | -2442.25 | 357.62 (-14.64%) | 248.95 (-10.19%) | -3048.83 (124.84%) |
| Uzbekistan | -28878.5 | 13240.71 (-45.85%) | 43081.17 (-149.18%) | -85200.35 (295.03%) |
| Vanuatu | 426.42 | 209.56 (49.14%) | 941.12 (220.7%) | -724.26 (-169.85%) |
| Venezuela (Bolivarian Republic of) | -10304.1 | 15502.11 (-150.45%) | 14511.2 (-140.83%) | -40317.35 (391.28%) |
| Viet Nam | -600752 | 311393.86 (-51.83%) | 404015.2 (-67.25%) | -1316160.67 (219.09%) |
| Yemen | -28868.9 | -3007.8 (10.42%) | 59664.64 (-206.67%) | -85525.72 (296.26%) |
| Zambia | -262230 | -15030.27 (5.73%) | 341506.49 (-130.23%) | -588706.41 (224.5%) |
| Zimbabwe | 270548.2 | 25193.42 (9.31%) | 145188.2 (53.66%) | 100166.62 (37.02%) |

4. Supplementary Table S4: Wald Chi-Square tests for estimable functions in the age-period-cohort model

| ASR of DALYs | | | | | | |
| --- | --- | --- | --- | --- | --- | --- |
|  | Both | P Value | Male | P Value | Female | P Value |
| NetDrift = 0 | 7707.3 | <0.001 | 6380.7 | <0.001 | 6404.2 | <0.001 |
| All Age Deviations = 0 | 33367.7 | <0.001 | 25007 | <0.001 | 31146.9 | <0.001 |
| All Period Deviations = 0 | 201.6 | <0.001 | 265.9 | <0.001 | 104.3 | <0.001 |
| All Cohort Deviations = 0 | 455.3 | <0.001 | 459.5 | <0.001 | 361.1 | <0.001 |
| All Period RR = 1 | 7768.1 | <0.001 | 6448.7 | <0.001 | 6505.4 | <0.001 |
| All Cohort RR = 1 | 17222.6 | <0.001 | 14241.4 | <0.001 | 14920.4 | <0.001 |
| All Local Drifts = Net Drift | 437.4 | <0.001 | 440.6 | <0.001 | 350.7 | <0.001 |

5. Supplementary Table S5: The slope index of inequality, concentration index, and their 95% confidence intervals for the age-standardized rate of disability-adjusted life-years of tuberculosis from 1990 to 2021.

| Year | Slope index of inequality (SII) | Concentration index (CI) |
| --- | --- | --- |
| 1990 | -5183.27 (-5636.08 to -4730.46) | -0.516 (-0.571 to -0.461) |
| 1991 | -5111.83 (-5543.92 to -4679.74) | -0.521 (-0.576 to -0.466) |
| 1992 | -4969.38 (-5395.94 to -4542.82) | -0.522 (-0.577 to -0.467) |
| 1993 | -4796.26 (-5211.74 to -4380.78) | -0.497 (-0.557 to -0.437) |
| 1994 | -4753.89 (-5176.49 to -4331.29) | -0.496 (-0.557 to -0.436) |
| 1995 | -4642.80 (-5051.20 to -4234.40) | -0.498 (-0.559 to -0.437) |
| 1996 | -4549.01 (-4970.87 to -4127.15) | -0.52 (-0.577 to -0.463) |
| 1997 | -4488.47 (-4912.09 to -4064.86) | -0.52 (-0.577 to -0.464) |
| 1998 | -4398.23 (-4821.03 to -3975.43) | -0.519 (-0.576 to -0.463) |
| 1999 | -4189.54 (-4608.74 to -3770.35) | -0.516 (-0.574 to -0.459) |
| 2000 | -4077.31 (-4488.19 to -3666.43) | -0.52 (-0.576 to -0.463) |
| 2001 | -3946.86 (-4345.05 to -3548.67) | -0.518 (-0.575 to -0.461) |
| 2002 | -3792.90 (-4183.44 to -3402.35) | -0.515 (-0.573 to -0.458) |
| 2003 | -3669.3 (-4054.59 to -3283.93) | -0.513 (-0.572 to -0.453) |
| 2004 | -3549.42 (-3914.15 to -3184.70) | -0.513 (-0.573 to -0.452) |
| 2005 | -3405.84 (-3768.18 to -3043.49) | -0.509 (-0.571 to -0.448) |
| 2006 | -3244.28 (-3586.15 to -2902.40) | -0.513 (-0.575 to -0.451) |
| 2007 | -3101.77 (-3435.71 to -2767.83) | -0.517 (-0.58 to -0.455) |
| 2008 | -2949.58 (-3277.66 to -2621.50) | -0.517 (-0.581 to -0.454) |
| 2009 | -2826.84 (-3148.53 to -2505.16) | -0.521 (-0.586 to -0.457) |
| 2010 | -2719.60 (-3033.62 to -2405.59) | -0.533 (-0.597 to -0.468) |
| 2011 | -2614.86 (-2928.61 to -2301.11) | -0.534 (-0.599 to -0.468) |
| 2012 | -2524.39 (-2820.05 to -2228.73) | -0.534 (-0.601 to -0.468) |
| 2013 | -2453.62 (-2732.99 to -2174.25) | -0.537 (-0.604 to -0.47) |
| 2014 | -2362.75 (-2628.63 to -2096.88) | -0.537 (-0.605 to -0.469) |
| 2015 | -2250.18 (-2510.38 to -1989.99) | -0.538 (-0.606 to -0.469) |
| 2016 | -2159.23 (-2405.26 to -1913.20) | -0.539 (-0.607 to -0.47) |
| 2017 | -2064.06 (-2294.79 to -1833.32) | -0.539 (-0.608 to -0.471) |
| 2018 | -1954.71 (-2176.29 to -1733.13) | -0.534 (-0.603 to -0.465) |
| 2019 | -1870.48 (-2084.09 to -1656.88) | -0.534 (-0.604 to -0.464) |
| 2020 | -1801.56 (-2004.30 to -1598.82) | -0.534 (-0.604 to -0.464) |
| 2021 | -1729.88 (-1927.35 to -1532.41) | -0.532 (-0.603 to -0.461) |

6. Supplementary Table S6: Projections of future burden of tuberculosis until 2035.

| **Location** | **Incidence rate (compared with 2015)** | | | **Number of Death** **(compared with 2015)** | | |
| --- | --- | --- | --- | --- | --- | --- |
|  | **2025** | **2030** | **2035** | **2025** | **2030** | **2035** |
| Global | 96.04   (-13.47%) | 89.54   (-19.33%) | 83.22   (-25.03%) | 1149145   (-10.80%) | 1097461   (-14.81%) | 1051320   (-18.40%) |
| Andean Latin America | 57.53   (-6.87%) | 53.47   (-13.45%) | 49.32   (-20.17%) | 4110   (-6.64%) | 3959   (-10.07%) | 4163   (-5.45%) |
| Australasia | 5.01   (-2.29%) | 4.88   (-4.86%) | 4.71   (-8.26%) | 78   (-1.88%) | 79   (-0.01%) | 83   (4.74%) |
| Caribbean | 32.31   (-10.39%) | 30.54   (-15.27%) | 28.79   (-20.14%) | 2434   (-10.37%) | 2328   (-14.27%) | 2275   (-16.2%) |
| Central Asia | 47.59   (-27.95%) | 39.86   (-39.66%) | 33.01   (-50.02%) | 3935   (-36.66%) | 3520   (-43.34%) | 3623   (-41.69%) |
| Central Europe | 12.01   (-29.1%) | 10.11   (-40.35%) | 8.5   (-49.83%) | 1386   (-46.35%) | 1089   (-57.85%) | 1081   (-58.15%) |
| Central Latin America | 18.21   (-10.68%) | 17.05   (-16.39%) | 15.89   (-22.08%) | 6709   (14.67%) | 7193   (22.93%) | 7846   (34.09%) |
| Central Sub-Saharan Africa | 356.32   (-21.26%) | 318.35   (-29.65%) | 283.87   (-37.27%) | 73321   (-8.01%) | 73354   (-7.97%) | 74785   (-6.17%) |
| East Asia | 34.37   (-25.32%) | 28.98   (-37.04%) | 24.47   (-46.83%) | 48116   (-16.2%) | 45609   (-20.56%) | 45285   (-21.13%) |
| Eastern Europe | 52.79   (-25.62%) | 47.46   (-33.13%) | 42.74   (-39.78%) | 7633   (-49.48%) | 6197   (-58.99%) | 5569   (-63.14%) |
| Eastern Sub-Saharan Africa | 253.59   (-24.61%) | 223.22   (-33.64%) | 196.68   (-41.53%) | 168025   (-2.95%) | 169983   (-1.82%) | 173385   (0.14%) |
| High-income Asia Pacific | 14.02   (-26.32%) | 11.78   (-38.13%) | 9.78   (-48.62%) | 6544   (5.46%) | 6784   (9.33%) | 7376   (18.86%) |
| High-income North America | 2.62   (18.19%) | 2.96   (33.39%) | 3.34   (50.87%) | 1018   (26%) | 1175   (45.48%) | 1404   (73.79%) |
| North Africa and Middle East | 25.66   (-25.96%) | 21.98   (-36.58%) | 18.79   (-45.78%) | 22492   (-3.68%) | 21577   (-7.6%) | 20986   (-10.13%) |
| Oceania | 116.3   (-10.93%) | 108.38   (-17%) | 100.96   (-22.68%) | 2999   (4.44%) | 3168   (10.32%) | 3380   (17.7%) |
| South Asia | 195.27   (-11.72%) | 184.52   (-16.58%) | 173.65   (-21.49%) | 531449   (-2.43%) | 525521   (-3.52%) | 527899   (-3.08%) |
| Southeast Asia | 170.86   (-11.86%) | 160.56   (-17.17%) | 151.07   (-22.07%) | 165131   (-12.03%) | 157065   (-16.33%) | 149240   (-20.5%) |
| Southern Latin America | 12.93   (-10.43%) | 12.06   (-16.46%) | 11.17   (-22.64%) | 1188   (-9.18%) | 1156   (-11.66%) | 1190   (-9.07%) |
| Southern Sub-Saharan Africa | 350.55   (-19.47%) | 316.76   (-27.23%) | 284.84   (-34.57%) | 44943   (3.29%) | 50569   (16.22%) | 62057   (42.62%) |
| Tropical Latin America | 27.61   (-14.29%) | 25.59   (-20.54%) | 23.6   (-26.74%) | 5685   (-6.52%) | 5476   (-9.96%) | 5281   (-13.16%) |
| Western Europe | 5.3   (-21.25%) | 4.61   (-31.55%) | 3.97   (-41%) | 2681   (-15.95%) | 2607   (-18.25%) | 2739   (-14.12%) |
| Western Sub-Saharan Africa | 151.1   (-35.06%) | 121.44   (-47.81%) | 97.49   (-58.1%) | 101080   (-16.72%) | 96520   (-20.48%) | 94218   (-22.38%) |

7. R codes used to conduct these analyses.

The original data are openly available in the Global Burden of Disease (GBD) database at <https://vizhub.healthdata.org/gbd-results/>. We initially processed data in Excel before further analyses in R. Some less relevant and repetitive codes are removed compared with the original R project.

**# 1. Estimated annual percentage change (EAPC)**

**## 1) EAPC calculation**

library(tidyverse)

EAPC <- read.csv('./rawdata/EAPC/TB_BAPC_regions_df.csv',header = T)

EAPC <- EAPC[,-1]

EAPC <- subset(EAPC, EAPC$age=='Age-standardized' &

EAPC$metric== 'Rate' &

EAPC$measure=='DALYs (Disability-Adjusted Life Years)' &

EAPC$sex=='Both') %>%

arrange(location,year)

EAPC_cal <- data.frame(Location=location,EAPC=rep(0,times=22),UCI=rep(0,times=22),LCI=rep(0,times=22))

for (i in 1:22){

country_cal <- as.character(EAPC_cal[i,1])

a <- subset(EAPC, EAPC$location==country_cal)

a$y <- log(a$val)

mod_simp_reg<-lm(y~year,data=a)

estimate <- (exp(summary(mod_simp_reg)[["coefficients"]][2,1])-1)*100

low <- (exp(summary(mod_simp_reg)[["coefficients"]][2,1]-1.96*summary(mod_simp_reg)[["coefficients"]][2,2])-1)*100

high <-(exp(summary(mod_simp_reg)[["coefficients"]][2,1]+1.96*summary(mod_simp_reg)[["coefficients"]][2,2])-1)*100

EAPC_cal[i,2] <- estimate

EAPC_cal[i,4] <- low

EAPC_cal[i,3] <- high

}

**## 2) EAPC worldmap**

library(sf)

library(patchwork)

library(ggplot2)

library(tidyverse)

GBD <- read.csv('./result/EAPC/TB_DALYs_EAPC_number.csv',header = T)

colnames(GBD)

GBD <- GBD[,-1]

names(GBD)[1] <- "location"

location <- read.csv("./rawdata/EAPC/location.csv")

colnames(location)

GBD <- left_join(GBD,location,by="location")

colnames(GBD)

map <- st_read("./rawdata/EAPC/map/worldmap.shp")

class(map)

colnames(map)

map <- st_set_crs(map,4326)

main_map_data <- left_join(map,GBD,by=c("NAME"="location3"))

head(main_map_data)

main_map_data <- na.omit(main_map_data)

main_map_data

p <- main_map_data %>%

ggplot()+

geom_sf(aes(group=NAME,fill=EAPC),color='black',size = 0.5) +

theme_void()+

scale_fill_distiller(palette="Spectral",

name="EAPC") +

labs(x="",y="",title="")+

theme(legend.position = c(0.1,0.2),

legend.title = element_text(color="black",

size = 10,

#family = "A",

#face = "bold"

),

plot.title = element_text(color="black",

size = 14,

#family = "A",

#face = "bold"

),

legend.text = element_text(color="black",

size = 10,

#family = "A",

#face = "bold"

),

panel.grid=element_blank(),

#legend.position = 'none',

axis.title.x = element_blank(),

axis.text.x = element_blank(),

axis.ticks.x = element_blank(),

axis.title.y = element_blank(),

axis.text.y = element_blank(),

axis.ticks.y = element_blank(),

)

worldData <- map_data('world')

small_map_data <- GBD

small_map_data$location[small_map_data$location == 'United States of America'] = 'USA'

small_map_data$location[small_map_data$location == 'Russian Federation'] = 'Russia'

small_map_data$location[small_map_data$location == 'United Kingdom'] = 'UK'

small_map_data$location[small_map_data$location == 'Congo'] = 'Republic of Congo'

small_map_data$location[small_map_data$location == "Iran (Islamic Republic of)"] = 'Iran'

small_map_data$location[small_map_data$location == "Democratic People's Republic of Korea"] = 'North Korea'

small_map_data$location[small_map_data$location == "Taiwan (Province of China)"] = 'Taiwan'

small_map_data$location[small_map_data$location == "Republic of Korea"] = 'South Korea'

small_map_data$location[small_map_data$location == "United Republic of Tanzania"] = 'Tanzania'

small_map_data$location[small_map_data$location == "Bolivia (Plurinational State of)"] = 'Bolivia'

small_map_data$location[small_map_data$location == "Venezuela (Bolivarian Republic of)"] = 'Venezuela'

small_map_data$location[small_map_data$location == "Czechia"] = 'Czech Republic'

small_map_data$location[small_map_data$location == "Republic of Moldova"] = 'Moldova'

small_map_data$location[small_map_data$location == "Viet Nam"] = 'Vietnam'

small_map_data$location[small_map_data$location == "Lao People's Democratic Republic"] = 'Laos'

small_map_data$location[small_map_data$location == "Syrian Arab Republic"] = 'Syria'

small_map_data$location[small_map_data$location == "North Macedonia"] = 'Macedonia'

small_map_data$location[small_map_data$location == "Micronesia (Federated States of)"] = 'Micronesia'

small_map_data$location[small_map_data$location == "Macedonia"] = 'North Macedonia'

small_map_data$location[small_map_data$location == "Trinidad and Tobago"] = 'Trinidad'

a <- small_map_data[small_map_data$location == "Trinidad",]

a$location <- 'Tobago'

small_map_data <- rbind(small_map_data,a)

small_map_data$location[small_map_data$location == "Cabo Verde"] = 'Cape Verde'

small_map_data$location[small_map_data$location == "United States Virgin Islands"] = 'Virgin Islands'

small_map_data$location[small_map_data$location == "Antigua and Barbuda"] = 'Antigu'

a <- small_map_data[small_map_data$location == "Antigu",]

a$location <- 'Barbuda'

small_map_data <- rbind(small_map_data,a)

small_map_data$location[small_map_data$location == "Saint Kitts and Nevis"] = 'Saint Kitts'

a <- small_map_data[small_map_data$location == "Saint Kitts",]

a$location <- 'Nevis'

small_map_data <- rbind(small_map_data,a)

small_map_data$location[small_map_data$location == "Cote"] = 'Ivory Coast'

small_map_data$location[small_map_data$location == "Saint Vincent and the Grenadines"] = 'Saint Vincenta'

small_map_data[small_map_data$location == "Saint Vincent",]

a$location <- 'Grenadines'

small_map_data <- rbind(small_map_data,a)

small_map_data$location[small_map_data$location == "Eswatini"] = 'Swaziland'

small_map_data$location[small_map_data$location == "Brunei Darussalam"] = 'Brunei'

small_map_data <- full_join(worldData,small_map_data,by = c('region'='location')) %>%

filter(EAPC != "NA")

dim(small_map_data)

head(small_map_data)

fig <- small_map_data %>%

ggplot()+

geom_polygon(aes(x = long, y = lat,group = group,fill=EAPC),

colour="black",size=0.5) +

theme_bw()+

scale_fill_distiller(palette="Spectral",

name="EAPC") +

theme(legend.position = 'none',

legend.title = element_blank(),

plot.title = element_text(color="black",

size = 10,

#family = "A",

#face = "bold"

),

legend.text = element_text(color="black",

size = 12,

#family = "A",

#face = "bold"

),

panel.grid=element_blank(),

panel.border = element_rect(color='black',

fill=NA,

size = 0.5),

#legend.position = 'none',

axis.title.x = element_blank(),

axis.text.x = element_blank(),

axis.ticks.x = element_blank(),

axis.title.y = element_blank(),

axis.text.y = element_blank(),

axis.ticks.y = element_blank(),

)

p2 <- fig+ labs(x=" ",y="",title="Caribbean and Central America")+

coord_cartesian(xlim = c(-92,-60),ylim = c(5,27))

p2

p3 <- fig+ labs(x=" ",y="",title="Persian Gulf")+

coord_cartesian(xlim = c(45,55),ylim = c(19,31))

p3

p4 <- fig+ labs(x=" ",y="",title="Balkan Peninsula")+

coord_cartesian(xlim = c(12,32),ylim = c(35,53))

p4

p5 <- fig+ labs(x=" ",y="",title="Southeast Asia")+

coord_cartesian(xlim = c(98,123),ylim = c(-10,8))

p5

p6 <- fig+ labs(x=" ",y="",title="West Africa") +

coord_cartesian(xlim = c(-17,-7),ylim = c(7,20))

p6

p7 <- fig+ labs(x=" ",y="",title="Eastern \nMediterranean")+

coord_cartesian(xlim = c(32,37),ylim = c(29,35))

p7

p8 <- fig+ labs(x=" ",y="",title="Northern Europe") +

coord_cartesian(xlim = c(5,25),ylim = c(48,60))

p8

A= (p6|p7)/p8

png(file = "./result/EAPC/TB_DALYs_EAPC_number_map.png",width = 6500,height = 4500,res = 600)

plot<- p +

(p2+p3+p4+p5+A+plot_layout(ncol = 5,widths=c(1.5,1,1.1,1.2,1)))+

plot_layout(ncol = 1,heights = c(9, 3))

plot

dev.off()

**# 2. Decomposition analysis**

library(dplyr)

library(data.table)

library(purrr)

library(tidyr)

library(ggplot2)

library(ggsci)

library(tidyverse)

case <- read.csv('./rawdata/decomposition/TB_decomposition_countries_df.csv')

case <- case[,-1]

case <- case %>%

mutate(age=sub('<5',replacement = '0 to 4', age))

unique(case$age)

ages <- c("0 to 4", "5 to 9","10 to 14", "15 to 19","20 to 24", "25 to 29",

"30 to 34", "35 to 39", "40 to 44", "45 to 49", "50 to 54", "55 to 59",

"60 to 64", "65 to 69", "70 to 74", "75 to 79", "80 to 84", "85 to 89",

"90 to 94", "95 plus")

case <- case %>%

filter(sex=='Both') %>%

filter(measure=='DALYs (Disability-Adjusted Life Years)') %>%

filter(age %in% ages) %>%

mutate(age=fct_relevel(age,ages)) %>%

arrange(age,year)

GBDpopulation_2021 <- read.csv("./rawdata/decomposition/GBDpopulation_2021.csv",header = T)

GBDpopulation_2021 <- GBDpopulation_2021[,-1]

unique(case$location)

pop <- GBDpopulation_2021 %>%

filter(year %in% c("1990", "1991", "1992", "1993", "1994", "1995", "1996", "1997", "1998", "1999", "2000", "2001", "2002", "2003", "2004", "2005", "2006", "2007", "2008", "2009", "2010", "2011", "2012", "2013", "2014", "2015", "2016", "2017", "2018", "2019", "2020", "2021")) %>%

filter(location %in% case$location) %>%

filter(age %in% ages) %>%

filter(sex=="Both")

pop <- pop %>%

mutate(age=fct_relevel(age,ages)) %>%

arrange(age,year)

unique(pop$age)

unique(pop$location)

decomposition_name <- c('location','overll_difference','a_effect','p_effect','r_effect','a_percent','p_percent','r_percent')

decomposition_data <- as.data.frame(matrix(nrow=0,ncol=length(decomposition_name)))

names(decomposition_data) <- decomposition_name

loc_name <- unique(pop$location)

a= loc_name[1]

for (a in loc_name) {

Global_population_1990 <- pop %>%

filter(location == a &

year == 1990 &

sex == 'Both') %>%

arrange(age)

Global_1990 <- sum(Global_population_1990$val)

Global_population_1990$percent <- Global_population_1990$val/Global_1990 Global_population_2021 <- pop %>%

filter(location == a &

year == 2021 &

sex == 'Both') %>%

arrange(age)

Global_2021 <- sum(Global_population_2021$val)

Global_population_2021$percent <- Global_population_2021$val/Global_2021

a_1990 <- Global_population_1990$percent

a_2021 <- Global_population_2021$percent

p_1990 <- Global_1990

p_2021 <- Global_2021

case_1990 <- case %>% filter(year == 1990 &

sex == 'Both' &

location == a &

metric == 'Rate' &

measure == 'DALYs (Disability-Adjusted Life Years)') %>%

arrange(age)

case_2021 <- case %>% filter(year == 2021 &

sex == 'Both' &

location == a &

metric == 'Rate' &

measure == 'DALYs (Disability-Adjusted Life Years)') %>%

arrange(age)

r_1990 <- as.numeric(case_1990$val)/10^5

r_2021 <- as.numeric(case_2021$val)/10^5

a_effect <- round((sum(a_2021*p_1990*r_1990) + sum(a_2021*p_2021*r_2021))/3 +

(sum(a_2021*p_1990*r_2021) + sum(a_2021*p_2021*r_1990))/6 -

(sum(a_1990*p_1990*r_1990) + sum(a_1990*p_2021*r_2021))/3 -

(sum(a_1990*p_1990*r_2021) + sum(a_1990*p_2021*r_1990))/6,3)

p_effect <- round((sum(a_1990*p_2021*r_1990) + sum(a_2021*p_2021*r_2021))/3 +

(sum(a_1990*p_2021*r_2021) + sum(a_2021*p_2021*r_1990))/6 -

(sum(a_1990*p_1990*r_1990) + sum(a_2021*p_1990*r_2021))/3 -

(sum(a_1990*p_1990*r_2021) + sum(a_2021*p_1990*r_1990))/6,3)

r_effect <- round((sum(a_1990*p_1990*r_2021) + sum(a_2021*p_2021*r_2021))/3 +

(sum(a_1990*p_2021*r_2021) + sum(a_2021*p_1990*r_2021))/6 -

(sum(a_1990*p_1990*r_1990) + sum(a_2021*p_2021*r_1990))/3 -

(sum(a_1990*p_2021*r_1990) + sum(a_2021*p_1990*r_1990))/6,3)

overll_differ <- round(a_effect + p_effect + r_effect,2)

a_percent <- round(a_effect/overll_differ*100,2)

p_percent <- round(p_effect/overll_differ*100,2)

r_percent <- round(r_effect/overll_differ*100,2)

temp <- c(a,overll_differ,a_effect,p_effect,r_effect,a_percent,

p_percent,r_percent) %>% t() %>% as.data.frame()

names(temp) <- decomposition_name

decomposition_data <- rbind(decomposition_data,temp)

}

data <- read.csv('./rawdata/decomposition/TB_decomposition_countries_df.csv')

num_1990 <- data %>% filter(age == 'All ages' &

sex == 'Both' &

metric == 'Number' &

year == 1990 &

measure == 'DALYs (Disability-Adjusted Life Years)') %>%

select(location, val) %>%

rename(val_1990 = val)

num_2021 <- data %>% filter(age == 'All ages' &

sex == 'Both' &

metric == 'Number' &

year == 2021 &

measure == 'DALYs (Disability-Adjusted Life Years)') %>%

select(location, val) %>%

rename(val_2021 = val)

decomposition_data <- left_join(decomposition_data,num_1990, by = 'location') %>%

left_join(num_2021, by = 'location')

decomposition_data$diff <- decomposition_data$val_2021 - decomposition_data$val_1990

decomposition_data[,2:11] <- decomposition_data[,2:11] %>% apply(c(1,2),as.numeric)

decomposition_data$check <- decomposition_data$diff-decomposition_data$overll_difference

round(decomposition_data$diff) == round(decomposition_data$overll_difference)

write.csv(decomposition_data,"./result/decomposition/TB_countries_decomposition.csv")

**#3. Age-period-cohort (APC) analysis**

**## 1) trend**

library(tidyverse)

library(data.table)

GBDpopulation_2021 <- read.csv("./rawdata/BAPC/GBDpopulation_2021.csv",header = T)

GBDpopulation_2021 <- GBDpopulation_2021[,-1]

age_stand <- read.csv('./rawdata/BAPC/std_pop2021.csv',header = T)

ages <- c("0 to 4", "5 to 9","10 to 14", "15 to 19","20 to 24", "25 to 29",

"30 to 34", "35 to 39", "40 to 44", "45 to 49", "50 to 54", "55 to 59",

"60 to 64", "65 to 69", "70 to 74", "75 to 79", "80 to 84", "85 to 89",

"90 to 94", "95 plus")

wstand <- age_stand$std_population/100 %>% as.numeric()

sum(wstand)

pop <- GBDpopulation_2021 %>%

filter(year %in% c("1990","1991","1992","1993","1994","1995","1996","1997","1998","1999", "2000","2001","2002","2003","2004","2005","2006","2007","2008","2009", "2010","2011","2012","2013","2014","2015","2016","2017","2018","2019",

"2020","2021")) %>%

filter(location == 'Global') %>%

filter(sex == 'Both') %>%

filter(age %in% ages)

pop <- pop %>%

mutate(age=fct_relevel(age,ages)) %>%

arrange(age)

disease <- read.csv("./rawdata/APC/TB_BAPC_global_df.csv") |>

filter(metric=="Rate")|>

filter(age !="All ages") |>

filter(age !="Age-standardized") |>

filter(location =="Global") |>

filter(sex == "Both")

disease$age[disease$age=="<5"] <- "0 to 4"

df <- left_join(disease,pop,by=c("age","year"))

df <- df[c(2,3,4,5,6,7,8,9,14)]

colnames(df)[8] <- "case"

colnames(df)[9] <- "population"

df <- df %>%

mutate(population = round(population,digits = 0)) %>%

mutate(case = round(case,digits = 0)) %>%

arrange(age,year)

df <- df[c(4,7,8,9)]

df$age[df$age=="95 plus"] <- "95 to 99"

df <- df |>

mutate(age2=age) |>

separate(age2,c("age2","other"),"to") |>

mutate(age2=as.numeric(age2)) |>

select(-other) |>

rename(period=year) |>

mutate(cohort=period-age2) |>

rename(age_group=age) |>

rename(age=age2) |>

mutate(period_group=case_when(

period < 1997~"1992~96",

period < 2002~"1997~01",

period < 2007~"2002~06",

period < 2012~"2007~11",

period < 2017~"2012~16",

period < 2021~"2017~21",

period == 2021~"2017~21"

))

df <- df |>

group_by(age_group,period_group) |>

mutate(case=round(mean(case),digits = 0)) |>

filter(period %in% seq(1992,2021,by=5)) |>

mutate(cohort_group=case_when(

cohort==1897~"1897~1901",

cohort==1902~"1902~1906",

cohort==1907~"1907~1911",

cohort==1912~"1912~1916",

cohort==1917~"1917~1921",

cohort==1922~"1922~1926",

cohort==1927~"1927~1931",

cohort==1932~"1932~1936",

cohort==1937~"1937~1941",

cohort==1942~"1942~1946",

cohort==1947~"1947~1951",

cohort==1952~"1952~1956",

cohort==1957~"1957~1961",

cohort==1962~"1962~1966",

cohort==1967~"1967~1971",

cohort==1972~"1972~1976",

cohort==1977~"1977~1981",

cohort==1982~"1982~1986",

cohort==1987~"1987~1991",

cohort==1992~"1992~1996",

cohort==1997~"1997~2001",

cohort==2002~"2002~2006",

cohort==2007~"2007~2011",

cohort==2012~"2012~2016",

cohort==2017~"2017~2021"

))

write.csv(df,"./result/APC/apc_trend_rate.csv",row.names = F)

library(tidyverse)

library(ggplot2)

library(ggsci)

library(patchwork)

mydata <- read.csv("./result/APC/apc_trend_rate.csv",header = T)

str(mydata)

mydata <- mydata %>%

arrange(age,period,cohort)

### period on age

pdf(file = "./result/APC/TB_period-on-age_trend.pdf",width = 7.2,height = 4.2)

p1 <- mydata %>%

ggplot(aes(x=age,y=case,group=period_group,color=period_group))+

geom_line(size=0.5)+

geom_point(shape=17,size=3)+

theme(plot.title = element_text(size = 20,hjust=0.5))+

guides(color=guide_legend(title = "period"))+

scale_fill_lancet()+

scale_color_lancet()+

scale_shape_discrete(guide ="none")+

xlab("Age groups")+

ylab("Age-standardized DALYs rate (per 100,000)")+

theme(axis.title = element_text(size=15))+

theme(axis.text = element_text(size = 12))+

theme_classic()+

scale_x_continuous(breaks = seq(0,95,by=5),

labels = c('0-4','5-9','10-14','15-19','20-24',

'25-29','30-34','35-39','40-44','45-49',

'50-54','55-59','60-64','65-69','70-74','75-79','80-84',

'85-89','90-94','95-99'))+

theme(axis.text.x = element_text(angle = 45,vjust = 0.5),

legend.position = c(1,1),

legend.justification = c(1,1),

legend.background = element_blank(),

legend.key.size = unit(0.1,"inches"))

p1

dev.off()

### cohort on age

pdf(file = "./result/APC/TB_cohort-on-age_trend.pdf",width = 7.2,height = 4.2)

p2 <- mydata %>%

ggplot(aes(x=period,y=case,group=age_group,color=age_group))+

geom_line(size=0.5)+

geom_point(size=3,shape=17)+

guides(color=guide_legend(title = "age",ncol = 2))+

theme(plot.title = element_text(size = 20,hjust=0.5))+

scale_fill_distiller(palette="Spectral")+

xlab("Period")+

ylab("Age-standardized DALYs rate (per 100,000)")+

theme(axis.title = element_text(size=15))+

theme(axis.text = element_text(size = 12))+

theme_classic()+

scale_x_continuous(breaks = seq(1992,2017,by=5),

labels = c('1992-1996','1997-2001','2002-2006','2007-2011','2012-2016',

'2017-2021'))+

theme(axis.text.x = element_text(vjust = 0.5),

legend.position = c(1,1),

legend.justification = c(1,1),

legend.background = element_blank(),

legend.key.size = unit(0.1,"inches"))

p2

dev.off()

### period on cohort

pdf(file = "./result/APC/TB_period-on-cohort_trend.pdf",width = 7.5,height = 4.2)

p3 <- mydata %>%

ggplot(aes(x=cohort,y=case,group=age_group,color=age_group))+

geom_line(size=0.5)+

geom_point(size=3,shape=17)+

theme(plot.title = element_text(size = 20,hjust=0.5))+

guides(color=guide_legend(title = "age",ncol = 1))+

scale_fill_distiller(palette="Spectral")+

xlab("Cohort")+

ylab("Age-standardized DALYs rate (per 100,000)")+

theme(axis.title = element_text(size=15))+

theme(axis.text = element_text(size = 12))+

theme_classic()+

scale_x_continuous(breaks = seq(1897,2022,by=5),

labels = c('1897','1902','1907','1912','1917',

'1922','1927','1932','1937','1942','1947','1952',

'1957','1962','1967','1972','1977','1982','1987','1992','1997',

'2002','2007','2012','2017','2022'))+

theme(axis.text.x = element_text(angle = 45,vjust = 0.5),

legend.position = c(1,1),

legend.justification = c(1,1),

legend.background = element_blank(),

legend.key.size = unit(0.1,"inches"))+

theme(legend.position = "right")

p3

dev.off()

pdf(file = "./result/APC/TB_APC_trend.pdf",width = 7.5,height = 12.6)

p <- p1/p2/p3

p

dev.off()

**## 2) effect**

library(tidyverse)

library(magrittr)

library(dplyr)

library(data.table)

source("./code/function_year5.R")

GBDpopulation_2021 <- read.csv("./rawdata/BAPC/GBDpopulation_2021.csv",header = T)

GBDpopulation_2021 <- GBDpopulation_2021[,-1]

age_stand <- read.csv('./rawdata/BAPC/std_pop2021.csv',header = T)

ages <- c("0 to 4", "5 to 9","10 to 14", "15 to 19","20 to 24", "25 to 29",

"30 to 34", "35 to 39", "40 to 44", "45 to 49", "50 to 54", "55 to 59",

"60 to 64", "65 to 69", "70 to 74", "75 to 79", "80 to 84", "85 to 89",

"90 to 94", "95 plus")

wstand <- age_stand$std_population/100 %>% as.numeric()

sum(wstand)

pop <- GBDpopulation_2021 %>%

filter(year %in% c("1990","1991","1992","1993","1994","1995","1996","1997","1998","1999", "2000","2001","2002","2003","2004","2005","2006","2007","2008","2009", "2010","2011","2012","2013","2014","2015","2016","2017","2018","2019",

"2020","2021")) %>%

filter(location == 'Global') %>%

filter(sex == 'Both') %>% ### “Both”, “Male”, “Female”

filter(age %in% ages)

pop <- pop %>%

mutate(age=fct_relevel(age,ages)) %>%

arrange(age)

unique(pop$age)

population_n <- dcast(data = pop[,-c(1:2)], age ~ year)

rownames(population_n) <- population_n$age

population_g <- function_year5(population_n, 1990, 2021, 2021)

rownames(population_g) <- population_n$age

disease <- read.csv("./rawdata/APC/TB_BAPC_global_df.csv") |>

filter(metric=="Number")|>

filter(age !="All ages") |>

filter(location =="Global") |>

filter(sex == "Both") |> ### “Both”, “Male”, “Female”

arrange (year)

disease <- disease[c(5,8,9)]

disease$age[disease$age=="<5"] <- "0 to 4"

disease_n <- dcast(data = disease, age ~ year)

disease_n <- disease_n[match(ages,disease_n$age),]

rownames(disease_n) <- disease_n$age

disease_g <- function_year5(disease_n, 1990, 2021, 2021)

rownames(disease_g) <- disease_n$age

name <- intersect(population_n$age,disease_n$age)

population_g <- population_g[rownames(population_g) %in% name,]

disease_g <- disease_incidence_g[rownames(disease_incidence_g) %in% name,]

for (i in 1:(2*ncol(population_g))){

if(i == 1){

DALY_population <-disease_g[,i] %>% as.data.frame()}

else{

if(i%%2==0){

DALY_population <- cbind(DALY_population,population_g[,ceiling(i/2)])}

else{

DALY_population <- cbind(DALY_population,disease_g[,ceiling(i/2)])}

}

}

names(DALY_population) <- rep(names(population_g),each=2)

write.csv(DALY_population,"./result/APC/APC_both.csv") ### “Both”, “Male”, “Female”

## The APC analysis was conducted in the APC webtool (https://analysistools.cancer.gov/apc/)

### longitudinal age curve

library(tidyverse)

library(ggsci)

df1 <- read.csv("./rawdata/APC/APC_longitudinal_age_curve.csv",header=T)

pdf(file = "./result/APC/APC_longitudinal_age_curve.pdf",width = 7.2,height = 4.2)

fig1 <- ggplot(data = df1, aes(x=Age,y=Rate,color=Sex))+

theme_classic()+

geom_line(size=0.5)+

geom_point(shape=17,size=3)+

theme(plot.title = element_text(size = 20,hjust=0.5))+

xlab(label = "Age groups")+

ylab(label = "Age-standardized DALYs rate (per 100,000)")+

theme(axis.title = element_text(size=11))+

scale_x_continuous(breaks = seq(2.5,97.5,by=5),

labels = c('0-4','5-9','10-14','15-19','20-24',

'25-29','30-34','35-39','40-44','45-49',

'50-54','55-59','60-64','65-69','70-74','75-79','80-84',

'85-89','90-94','95-99'))+

theme(axis.text = element_text(size = 15))+

scale_fill_lancet()+

scale_color_lancet()+

theme(axis.text.x = element_text(angle = 45,vjust = 0.5),

legend.position = c(1,1),

legend.justification = c(1,1),

legend.background = element_blank(),

legend.key.size = unit(0.1,"inches"))+

geom_segment(x=-2,xend=100,

y=1,yend=1,

color="grey",linetype=2,size=0.5,alpha=0.8)

fig1

dev.off()

### APC period RR

rm(list=ls())

library(tidyverse)

library(ggsci)

df2 <- read.csv("./rawdata/APC/APC_period_RR.csv",header=T)

pdf(file = "./result/APC/APC_period_RR.pdf",width = 7.2,height = 4.2)

fig2 <- ggplot(data = df2, aes(x=Period,y=Rate.Ratio,color=Sex))+

theme_classic()+

geom_line(size=0.5)+

geom_point(shape=17,size=3)+

theme(plot.title = element_text(size = 20,hjust=0.5))+

xlab(label = "Period")+

ylab(label = "Rate Ratio")+

theme(axis.title = element_text(size=15))+

scale_x_continuous(limits = c(1992,2022),

breaks = seq(1994.5,2021.5,by=5),

labels = c('1992-1996','19972001','2002-2006','2007-2011','2012-2016',

'2017-2021'))+

theme(axis.text = element_text(size = 12))+

scale_fill_lancet()+

scale_color_lancet()+

theme(axis.text.x = element_text(vjust = 0.5),

legend.position = c(1,1),

legend.justification = c(1,1),

legend.background = element_blank(),

legend.key.size = unit(0.1,"inches"))+

geom_segment(x=0,xend=2025,

y=1,yend=1,

color="grey",linetype=2,size=0.5,alpha=0.8)

fig2

dev.off()

### APC cohort RR

rm(list=ls())

library(tidyverse)

library(ggsci)

df3 <- read.csv("./rawdata/APC/APC_cohort_RR.csv",header=T)

pdf(file = "./result/APC/APC_cohort_RR.pdf",width = 7.2,height = 4.2)

fig3 <- ggplot(data = df3, aes(x=Cohort,y=Rate.Ratio,color=Sex))+

theme_classic()+

geom_line(size=0.5)+

geom_point(shape=17,size=3)+

theme(plot.title = element_text(size = 20,hjust=0.5))+

xlab(label = "Cohort")+

ylab(label = "Rate Ratio")+

theme(axis.title = element_text(size=15))+

scale_x_continuous(limits = c(1897,2017),

breaks = seq(1897,2017,by=5),

labels = c('1897','1902','1907','1912','1917',

'1922','1927','1932','1937','1942','1947','1952',

'1957','1962','1967','1972','1977','1982','1987','1992',

'1997','2002','2007','2012','2017'))+

theme(axis.text = element_text(size = 12))+

scale_fill_lancet()+

scale_color_lancet()+

theme(axis.text.x = element_text(angle=45,vjust = 0.5),

legend.position = c(1,1),

legend.justification = c(1,1),

legend.background = element_blank(),

legend.key.size = unit(0.1,"inches"))+

geom_segment(x=0,xend=2020,

y=1,yend=1,

color="grey",linetype=2,size=0.5,alpha=0.8)

fig3

dev.off()

**# 4. Health inequality analysis**

**## 1) Slope index of inequality**

library(tidyverse)

library(data.table)

library(ggbrace)

library(mgcv)

library(splines)

library(broom)

library(ggplot2)

burden <- read.csv("./rawdata/inequality/TB_inequality_2021_df.csv")

burden <- burden |>

filter(age == "Age-standardized") |>

filter(metric == "Rate") |>

arrange(year) %>%

arrange(location)

SDI <- read.csv("rawdata/inequality/SDI_204countries.csv",header = T)

a1 <- unique(burden$location)

a2 <- unique(SDI$location_name)

setdiff(a1,a2)

data <- left_join(burden,SDI,by=c("location","year"))

sum(is.na(data))

b1 <- unique(burden$location)

b2 <- unique(SDI$location)

setdiff(b1,b2)

pop <- read.csv("rawdata/inequality/GBDpopulation_204countries.csv",header = T)

mydata <- left_join(data,pop,

by=c("location","year"))

sum(is.na(mydata))

mydata1 <- read.csv("./rawdata/inequality/TB_inequality_mydata1.csv",header = T)

sum(is.na(mydata1))

mydata2 <- mydata1 |>

arrange(year,sdi) |>

mutate(se=(upper-lower)/1.96/2) |>

group_by(year) |>

mutate(order=1:204)

write.csv(mydata2,"./result/inequality/TB_inequality_order.csv")

colnames(mydata2)

unique(mydata2$age)

unique(mydata2$metric)

a <- mydata2 |>

group_by(year) |>

summarise(sum=sum(pop))

pop1990 <- a$sum[1]

pop1991 <- a$sum[2]

pop1992 <- a$sum[3]

pop1993 <- a$sum[4]

pop1994 <- a$sum[5]

pop1995 <- a$sum[6]

pop1996 <- a$sum[7]

pop1997 <- a$sum[8]

pop1998 <- a$sum[9]

pop1999 <- a$sum[10]

pop2000 <- a$sum[11]

pop2001 <- a$sum[12]

pop2002 <- a$sum[13]

pop2003 <- a$sum[14]

pop2004 <- a$sum[15]

pop2005 <- a$sum[16]

pop2006 <- a$sum[17]

pop2007 <- a$sum[18]

pop2008 <- a$sum[19]

pop2009 <- a$sum[20]

pop2010 <- a$sum[21]

pop2011 <- a$sum[22]

pop2012 <- a$sum[23]

pop2013 <- a$sum[24]

pop2014 <- a$sum[25]

pop2015 <- a$sum[26]

pop2016 <- a$sum[27]

pop2017 <- a$sum[28]

pop2018 <- a$sum[29]

pop2019 <- a$sum[30]

pop2020 <- a$sum[31]

pop2021 <- a$sum[32]

rank <- mydata2 |>

mutate(pop_global=ifelse(year==1990,pop1990,ifelse(year==1991,pop1991,ifelse(year==1992,pop1992,ifelse(year==1993,pop1993,ifelse(year==1994,pop1994,ifelse(year==1994,pop1994,ifelse(year==1995,pop1995,ifelse(year==1996,pop1996,ifelse(year==1997,pop1997,ifelse(year==1998,pop1998,ifelse(year==1999,pop1999,ifelse(year==2000,pop2000,ifelse(year==2001,pop2001,ifelse(year==2002,pop2002,ifelse(year==2003,pop2003,ifelse(year==2004,pop2004,ifelse(year==2005,pop2005,ifelse(year==2006,pop2006,ifelse(year==2007,pop2007,ifelse(year==2008,pop2008,ifelse(year==2009,pop2009,ifelse(year==2010,pop2010,ifelse(year==2011,pop2011,ifelse(year==2012,pop2012,ifelse(year==2013,pop2013,ifelse(year==2014,pop2014,ifelse(year==2015,pop2015,ifelse(year==2016,pop2016,ifelse(year==2017,pop2017,ifelse(year==2018,pop2018,ifelse(year==2019,pop2019,ifelse(year==2020,pop2020,pop2021))))))))))))))))))))))))))))))))) |>

group_by(year,metric) |>

arrange(year,metric,sdi) |>

mutate(cummu=cumsum(pop)) |>

mutate(half=pop/2) |>

mutate(midpoint=cummu-half) |>

mutate(weighted_order=midpoint/pop_global)

write.csv(rank,"./result/inequality/TB_inequality_rank.csv")

temp1 <- rank |> filter(metric=="Rate") |> filter(year==1990)

temp32 <- rank |> filter(metric=="Rate") |> filter(year==2021)

library(car)

library(MASS)

fit1 <- lm(data = temp1,val~weighted_order)

ncvTest(fit1)

r.huber1 <- rlm(data = temp1,val~weighted_order)

coef(r.huber1)

confint.default(r.huber1)

fit32 <- lm(data = temp32,val~weighted_order)

ncvTest(fit32)

r.huber32 <- rlm(data = temp32,val~weighted_order)

coef(r.huber32)

confint.default(r.huber32)

mydata <- rank %>%

filter(year %in% c("1990","2010","2021"))

mydata$year <- factor(mydata$year)

color <- c("#C72228","#F98F34","#0C4E9B")

pdf(file = "./result/inequality/TB_DALY_SII.pdf",width = 7,height = 5.2)

p1 <- mydata %>%

ggplot(aes(x=weighted_order,y=val,fill=year,group=year,color=year))+

theme_classic()+

geom_point(aes(color=year,size=pop/1e6),alpha=0.8,shape=21)+

scale_size_area("Population\n(million)",breaks=c(200,400,600,800,1000,1200))+

geom_smooth(method = "rlm",size=0.6,alpha=0.2)+

scale_fill_manual(values = color)+

scale_color_manual(values = color)+

theme(axis.title = element_text(size=17))+

theme(axis.text = element_text(size = 15))+

theme(legend.text = element_text(size = 12))+

scale_x_continuous(limits = c(0,1),

breaks = seq(0,1,by=0.25),

labels = c("0","0.25","0.50","0.75","1.00"))+

theme(legend.position = c(1,1),

legend.justification=c(1,1))+

xlab("Relative rank by SDI")+

ylab("Age-standardized DALYs rate (per 100,000)")

p1

dev.off()

pdf(file = "./result/inequality/TB_DALYs_SII_1990-2021.pdf",width = 6,height = 2)

fig1 <- ggplot(data = df, aes(x=year,y=rlm))+

theme_gray(base_size = 12)+

geom_smooth(method = "lm")+

geom_point(size=2.5)+

xlab(label = "Year")+

ylab(label = "SII")

fig1

dev.off()

**## 2) Concentration index**

**###** The concentration index is calculated in Stata instead of R

library(tidyverse)

library(data.table)

burden <- read.csv("./rawdata/inequality/TB_inequality_2021_df.csv")

burden <- burden |>

filter(age == "Age-standardized") |>

filter(metric == "Rate") |>

filter(year %in% c("1990","2010","2021") ) |>

arrange(year) %>%

arrange(location)

mydata <- read.csv("./rawdata/inequality/TB_inequality_mydata1.csv",header = T)

mydata <- mydata |>

filter(year %in% c("1990","2010","2021") ) |>

arrange(year) %>%

arrange(location)

sum(is.na(mydata))

a <- mydata %>%

group_by(year) %>%

summarise(sum=sum(pop))

pop1990 <- a$sum[1]

pop2010 <- a$sum[2]

pop2021 <- a$sum[3]

rank <- mydata %>% mutate(pop_global=ifelse(year==1990,pop1990,ifelse(year==2010,pop2010,pop2021))) %>%

group_by(year,metric) %>%

arrange(sdi) %>%

mutate(cummu=cumsum(pop)) %>%

mutate(half=pop/2) %>%

mutate(midpoint=cummu-half) %>%

mutate(weighted_order=midpoint/pop_global)

rank$year <- factor(rank$year)

b <- mydata %>%

filter(metric=="Rate") %>%

group_by(year) %>%

summarise(sum=sum(val))

burden1990 <- b$sum[1]

burden2010 <- b$sum[2]

burden2021 <- b$sum[3]

ci <- rank %>%

filter(metric=="Rate") %>%

mutate(total_daly=ifelse(year==1990,burden1990,ifelse(year==2010,burden2010,burden2021))) %>%

group_by(year) %>%

arrange(sdi) %>%

mutate(cummu_daly=cumsum(val)) %>%

mutate(frac_daly=cummu_daly/total_daly) %>%

mutate(frac_population=cummu/pop_global)

color <- c("#C72228","#F98F34","#0C4E9B")

pdf(file = "./result/inequality/TB_DALYs_CI.pdf",width = 8,height = 6)

p2 <- ci %>%

ggplot(aes(x=frac_population,y=frac_daly,fill=year,color=year,group=year))+

theme_classic()+

geom_segment(x=0,xend=0,

y=0,yend=1,

linetype=4,size=0.8,color="gray")+

geom_segment(x=0,xend=1,

y=1,yend=1,

linetype=4,size=0.8,color="gray")+

geom_segment(x=0,xend=1,

y=0,yend=1,

color="grey",linetype=4,size=1,alpha=1)+

geom_point(aes(fill=year,size=pop/1e6),alpha=0.8,shape=21)+

scale_fill_manual(values = color)+

scale_size_area("Population\n(million)",breaks=c(200,400,600,800,1000,1200))+

geom_smooth(method = "gam",

linetype=1,size=0.6,alpha=0.2,se=T)+

scale_color_manual(values = color)+

xlab("Cumulative fraction of population ranked by SDI")+

ylab("Cumulative fraction of DALYs")+

theme(axis.title = element_text(size=17))+

theme(axis.text = element_text(size = 15))+

theme(legend.text = element_text(size = 12))+

theme(legend.position = c(0.9,0.4))

p2

dev.off()

df <- read.csv("./result/inequality/TB_DALY_2021_CI.csv",header = T)

pdf(file = "./result/inequality/TB_DALYs_CI_1990-2021.pdf",width = 6,height = 2)

fig1 <- ggplot(data = df, aes(x=year,y=Concentration_index))+

theme_gray(base_size = 12)+

geom_smooth(method = "lm")+

geom_point(size=2.5)+

xlab(label = "Year")+

ylab(label = "CI")

fig1

dev.off()

**# 5. frontier analysis**

library(dplyr)

library(data.table)

library(purrr)

library(tidyr)

library(ggplot2)

library(ggrepel)

mydata <- read.csv("./rawdata/frontier/TB_inequality_mydata1.csv",header = T)

sum(is.na(mydata))

frontier_SDI <- mydata %>%

select(3,8,9,12) %>%

rename(ASR = val) %>%

rename(SDI = sdi)

sum(is.na(frontier_SDI))

boostrap_DEA <- as.data.frame(matrix(nrow=0,ncol=6))

names(boostrap_DEA) <- c(names(frontier_SDI),'frontier','super')

boostrap_num <- 100

for(interation_number in 1:boostrap_num){

boot_sample <- frontier_SDI[sample(1:nrow(frontier_SDI),nrow(frontier_SDI),replace = TRUE),]

boot_sample <- boot_sample %>% arrange(SDI,desc(ASR))

boot_sample$super <- NA

boot_sample$super[1] <- 0

for (i in 2:nrow(frontier_SDI)) {

data <- boot_sample[-i,]

data$frontier <- NA

min <- data$ASR[1]

for (j in 1:(i-1)) {

min <- ifelse(data$ASR[j]<min,data$ASR[j],min)

data$frontier[j] <- min}

boot_sample$super[i] <- ifelse(boot_sample[i,1]==boot_sample[i+1,1] & boot_sample[i,2]==boot_sample[i+1,2],0,

ifelse(boot_sample$ASR[i]<data$frontier[i-1],1,0))

}

boot_sample_exclude <- boot_sample[boot_sample$super==0,]

min <- boot_sample_exclude$ASR[1]

for (z in 1:nrow(boot_sample_exclude)){

min <- ifelse(boot_sample_exclude$ASR[z]<min,boot_sample_exclude$ASR[z],min)

boot_sample_exclude$frontier[z] <- min

}

boostrap_DEA <- rbind(boostrap_DEA,boot_sample_exclude)

boostrap_DEA <-boostrap_DEA %>%

group_by(location,year,ASR,SDI) %>%

summarize(frontier=mean(frontier))

print(interation_number)

}

boostrap_DEA <- boostrap_DEA %>%

mutate(eff_diff = ASR - frontier)

boostrap_DEA_2021 <- boostrap_DEA %>%

filter(year == 2021)

pdf(file = "./result/frontier/TB_boostrap_frontier_100times.pdf",width = 10,height = 7)

plotA <- ggplot(boostrap_DEA, aes(SDI,ASR)) + geom_point(aes(color = year),size=1.8)+

scale_x_continuous(breaks = c(0,0.2,0.4,0.6,0.8,1.0),limits = c(0,1)) +

scale_y_reverse() +

scale_color_gradient(low='#479F94',high='#EDAC5D') +

stat_smooth(data=boostrap_DEA, aes(SDI,frontier),colour='black',formula=y ~ poly(x, 1),

stat = "smooth",method='loess',se=F,span=0.2,fullrange=T) +

theme_bw()+

theme(axis.title = element_text(size=20))+

theme(axis.text = element_text(size = 17))+

theme(legend.text = element_text(size = 15))+

theme(legend.title = element_text(size = 17))+

ylab(label = "Age-standardized DALYs rate (per 100,000)")+

xlab(label = "Socio-demographic index")

plotA

dev.off()

a <- boostrap_DEA_2021[order(boostrap_DEA_2021$eff_diff,decreasing = T),][1:5,]

b <- subset(boostrap_DEA_2021,SDI<0.455)[order(subset(boostrap_DEA_2021,SDI<0.455)$eff_diff),][1:3,]

c <- subset(boostrap_DEA_2021,SDI>0.805)[order(subset(boostrap_DEA_2021,SDI>0.805)$eff_diff,decreasing = T),][1:5,]

boostrap_DEA_2021$color <- ifelse(boostrap_DEA_2021$eff_diff > 10, "1","2")

pdf(file = "./result/frontier/TB_boostrap_frontier_100times_1.3.pdf",width = 10,height = 7)

plotB <- ggplot(boostrap_DEA_2021, aes(SDI,ASR)) + geom_point(aes(color = color),size=3.5)+

scale_x_continuous(breaks = c(0,0.2,0.4,0.6,0.8,1.0),limits = c(0,1)) +

scale_y_reverse() +

stat_smooth(data=boostrap_DEA, aes(SDI,frontier),colour='black',formula=y ~ poly(x, 1),

stat = "smooth",method='loess',se=F,span=0.2,fullrange=T) +

geom_text_repel(data=a,colour='#BF1D2D',aes(SDI,ASR, label = location),size=3.5,fontface= 'bold',max.overlaps = 160) +

geom_text_repel(data=b,colour='#262626',aes(SDI,ASR, label = location),size=3.5,fontface= 'bold',max.overlaps = 160) +

geom_text_repel(data=c,colour='#293890',aes(SDI,ASR, label = location),size=3.5,fontface= 'bold',max.overlaps = 160) +

theme_bw()+

theme(axis.title = element_text(size=20))+

theme(axis.text = element_text(size = 17))+

theme(legend.text = element_text(size = 17))+

ylab(label = "Age-standardized DALYs rate (per 100,000)")+

xlab(label = "Socio-demographic index")

plotB

dev.off()

**# 6. Bayesian age-period-cohort (BAPC) analysis**

library(BAPC)

library(INLA)

library(data.table)

library(tidyverse)

library(dplyr)

library(ggplot2)

library(epitools)

library(reshape2)

df <- read.csv("./rawdata/BAPC/TB_BAPC_incidence_death_df.csv",header = T)

df <- df[,-1]

df <- df %>%

mutate(age=sub('<5',replacement = '0 to 4', age))

unique(df$age)

age_stand <- read.csv('./rawdata/BAPC/std_pop2021.csv',header = T)

ages <- c("0 to 4", "5 to 9","10 to 14", "15 to 19","20 to 24", "25 to 29",

"30 to 34", "35 to 39", "40 to 44", "45 to 49", "50 to 54", "55 to 59",

"60 to 64", "65 to 69", "70 to 74", "75 to 79", "80 to 84", "85 to 89",

"90 to 94", "95 plus")

wstand <- age_stand$std_population/100 %>% as.numeric()

df_Both_n <- reshape2::dcast(data = df_Both, year~age, value.var = "val")

df_Both_n <- df_Both_n %>%

arrange(year)

rownames(df_Both_n) <- df_Both_n$year

df_Both_n <- df_Both_n[,-1]

df_Both_n <- df_Both_n %>%

apply(c(1,2), as.numeric) %>%

apply(c(1,2), round) %>%

as.data.frame() %>%

select(ages)

GBDpopulation_2021 <- read.csv("./rawdata/BAPC/GBDpopulation_2021.csv",header = T)

GBDpopulation_2021 <- GBDpopulation_2021[,-1]

unique(df$location)

pop <- GBDpopulation_2021 %>%

filter(year %in% c("1990", "1991", "1992", "1993", "1994", "1995", "1996", "1997", "1998", "1999", "2000", "2001", "2002", "2003", "2004", "2005", "2006", "2007", "2008", "2009", "2010", "2011", "2012", "2013", "2014", "2015", "2016", "2017", "2018", "2019", "2020", "2021")) %>%

filter(location %in% df$location) %>%

filter(age %in% ages)

pop <- pop %>%

mutate(age=fct_relevel(age,ages)) %>%

arrange(age)

unique(pop$age)

var <- c('location_id',"location","sex","year","age","val")

prediction_var <- c("location_name","sex","year_id","age_group_name","val")

GBD_population_prediction <- fread('./rawdata/BAPC/IHME_POP_2017_2100_POP_REFERENCE_Y2020M05D01.csv') %>%

as.data.frame() %>%

select(prediction_var) %>%

filter(location_name %in% df$location) %>%

filter(year_id %in% 2022:2035)

names(GBD_population_prediction) <- var[-1]

unique(GBD_population_prediction$age)

unique(GBD_population_prediction$location)

unique(GBD_population_prediction$year)

unique(GBD_population_prediction$sex)

GBD_1year <- GBD_population_prediction %>%

filter(age %in% c("Early Neonatal",

"Late Neonatal", "Post Neonatal")) %>%

group_by(location,sex,year) %>%

summarise(val=sum(val)) %>%

mutate(age="<1 year") %>%

select(var[-1])

GBD_population_prediction <- GBD_population_prediction %>% filter(!(age %in% c("Early Neonatal","Late Neonatal", "Post Neonatal"))) %>%

rbind(GBD_1year)

unique(GBD_population_prediction$age)

GBD_age4 <- GBD_population_prediction %>% subset(age %in% c("<1 year","1 to 4")) %>%

group_by(location,sex,year) %>%

summarize(val=sum(val)) %>% mutate(age='0 to 4') %>%

select(var[-1])

GBD_population_prediction <- rbind(GBD_population_prediction,GBD_age4)

GBD_population_prediction <- subset(GBD_population_prediction, age %in% ages) %>%

mutate(age=fct_relevel(age,ages)) %>%

arrange(age)

unique(GBD_population_prediction$age)

GBD <- rbind(pop,GBD_population_prediction)

unique(GBD$age)

GBD_Global_Male <- GBD %>% filter(location=='Global' & sex == 'Male')

GBD_Global_Female <- GBD %>% filter(location=='Global' & sex == 'Female')

GBD_Global_Male_n <- reshape2::dcast(data = GBD_Global_Male,

year~ age,

value.var = c("val"))

GBD_Global_Male_n <- GBD_Global_Male_n %>%

arrange(year)

rownames(GBD_Global_Male_n) <- GBD_Global_Male_n$year

GBD_Global_Male_n <- GBD_Global_Male_n[,-1]

GBD_Global_Female_n <- reshape2::dcast(data = GBD_Global_Female,

year ~ age,value.var = c("val"))

GBD_Global_Female_n <- GBD_Global_Female_n %>%

arrange(year)

rownames(GBD_Global_Female_n) <- GBD_Global_Female_n$year

GBD_Global_Female_n <- GBD_Global_Female_n[,-1]

GBD_Global_Both_n <- GBD_Global_Female_n + GBD_Global_Male_n

df_pro <- matrix(data = NA, nrow = 2035-2021, ncol = ncol(GBD_Global_Male_n)) %>% as.data.frame()

rownames(df_pro) <- seq(2022,2035,1)

colnames(df_pro) <- names(df_Both_n)

df_Both_n <- rbind(df_Both_n , df_pro)

Both_esoph <- APCList(df_Both_n, GBD_Global_Both_n, gf = 5)

Both_bapc_result <- BAPC(Both_esoph, predict = list(npredict = 14, retro = T),

secondDiff = FALSE, stdweight = wstand, verbose = F)

Both_ASR <- agestd.rate(x = Both_bapc_result) %>% as.data.frame()

Both_ASR$mean <- Both_ASR$mean*100000

Both_ASR$year <- rownames(Both_ASR)

year_index <- 1990:2035

write.csv(Both_ASR,"./result/BAPC/BAPC_incidence/TB_BAPC_incidence_22Global.csv")

Both_proj <- agespec.proj(x = Both_bapc_result) %>% as.data.frame()

Both_proj_mean <- Both_proj[,colnames(Both_proj) %like% 'mean']

names(Both_proj_mean) <- ages

Both_sum_year <- apply(Both_proj_mean, 1, sum) %>% as.data.frame()

colnames(Both_sum_year) <- 'number'

Both_sum_year$year <- rownames(Both_sum_year)

write.csv(Both_sum_year,"./result/BAPC/BAPC_death/TB_BAPC_death_22Global.csv")
