# Supplementary material for: Applying Multiple Analyses to Quantify Changes in the Global, Regional, and National Burden of Tuberculosis From 1990 to 2021 and the Forecast Until 2035
Source: Can J Infect Dis Med Microbiol. 2025 Nov 20;2025:8899003. doi: 10.1155/cjid/8899003 (PMC12659984; doi:10.1155/cjid/8899003)
Supplement: Supporting Information — Additional supporting information can be found online in the Supporting Information section. [file 8899003.f1.zip › GBD_TB.zip!/GBD_TB_2021_upload/result/APC/APC_cohort_RR.pdf]

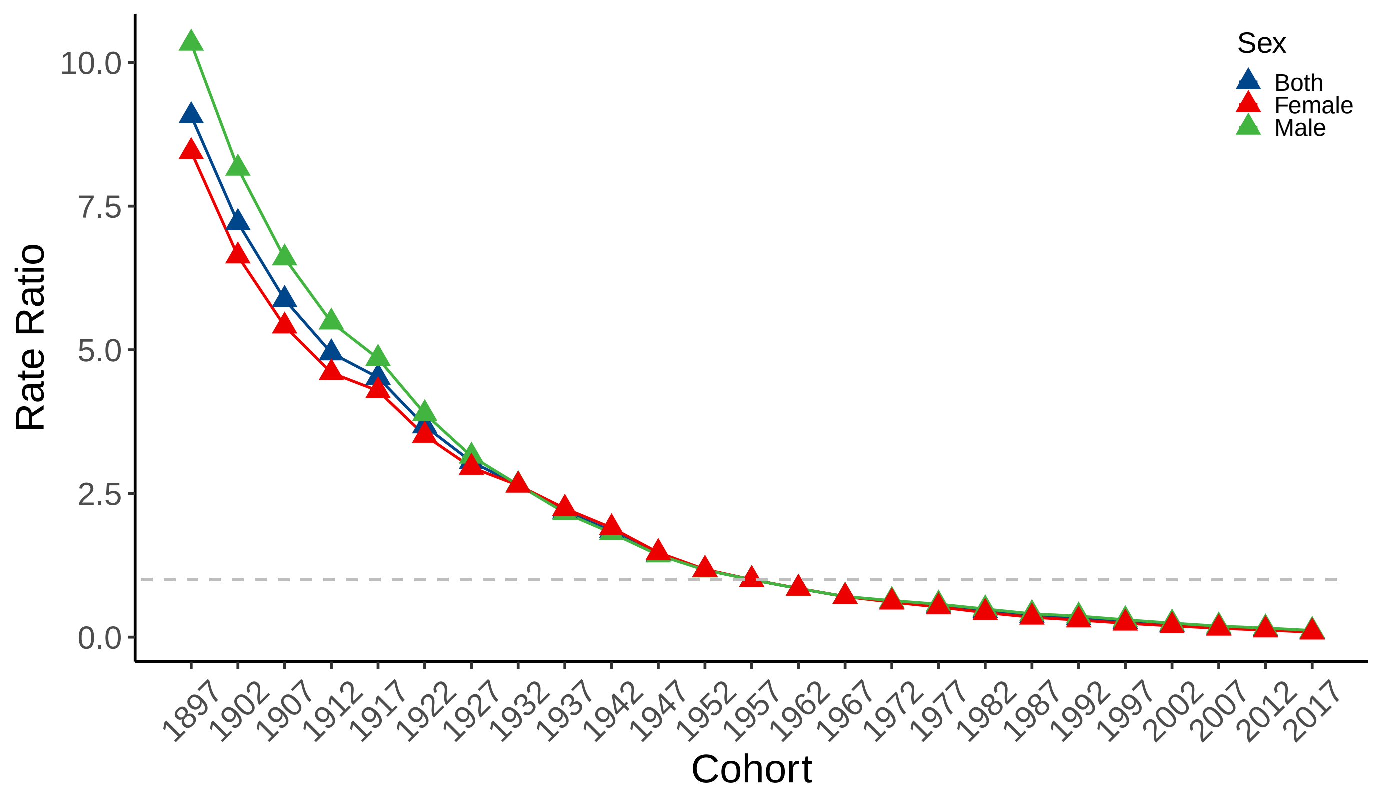
Sex
Both
Female
Male
Rate Ratio
10.0
7.5
5.0
2.5
0.0
1897
1902
1907
1912
1917
1922
1927
1932
1937
1942
1947
1952
1957
1962
1967
1972
1977
1982
1987
1992
1997
2002
2007
2012
2017
Cohort
