# Supplementary material for: Applying Multiple Analyses to Quantify Changes in the Global, Regional, and National Burden of Tuberculosis From 1990 to 2021 and the Forecast Until 2035
Source: Can J Infect Dis Med Microbiol. 2025 Nov 20;2025:8899003. doi: 10.1155/cjid/8899003 (PMC12659984; doi:10.1155/cjid/8899003)
Supplement: Supporting Information — Additional supporting information can be found online in the Supporting Information section. [file 8899003.f1.zip › GBD_TB.zip!/GBD_TB_2021_upload/result/APC/TB_APC_trend.pdf]

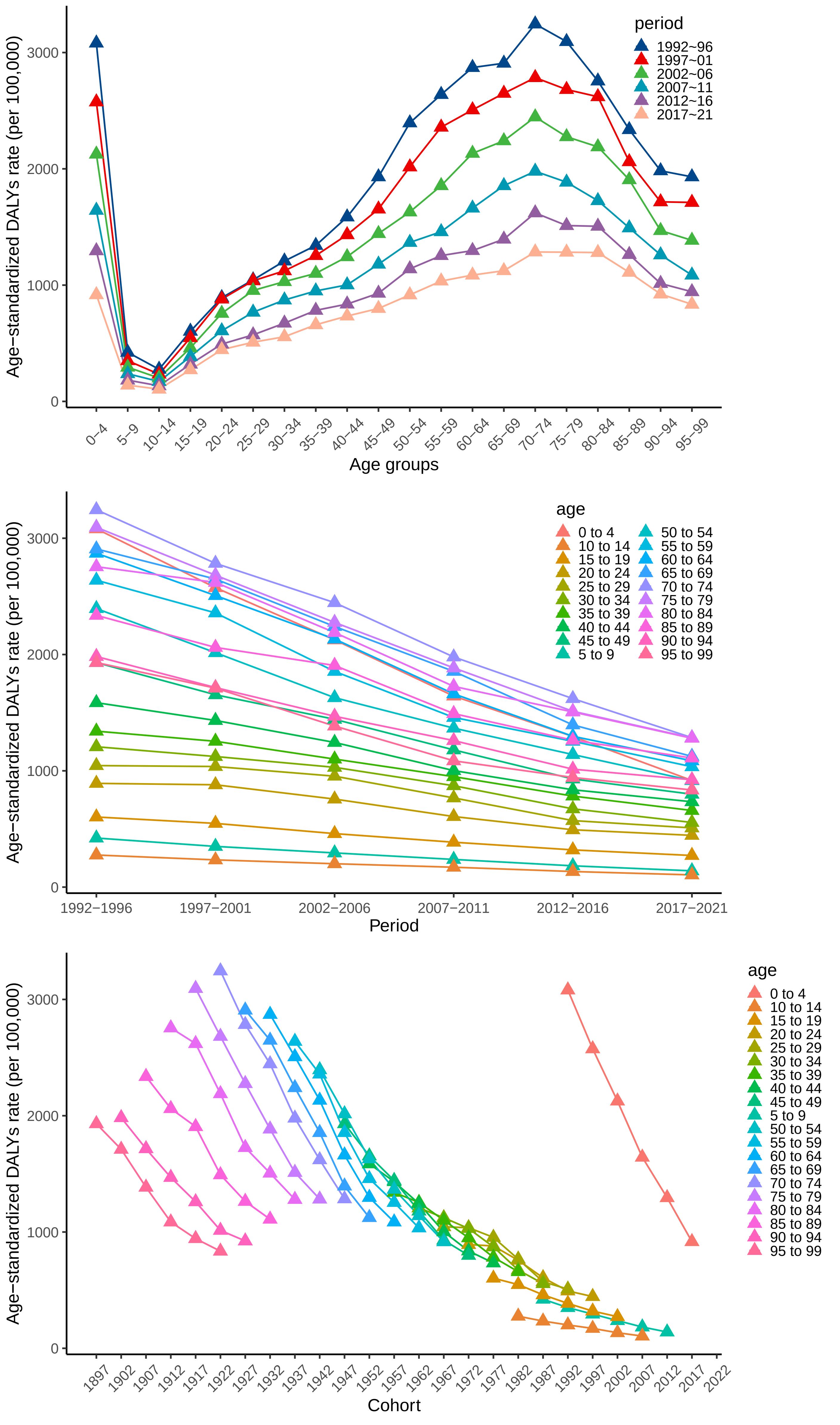

period
1992~96
1997~01
2002~06
2007~11
2012~16
2017~21
Age−standardized DALYs rate (per 100,000)
0
1000
2000
3000
0−4
5−9
10−14
15−19
20−24
25−29
30−34
35−39
40−44
45−49
50−54
55−59
60−64
65−69
70−74
75−79
80−84
85−89
90−94
95−99
Age groups
age
0 to 4
10 to 14
15 to 19
20 to 24
25 to 29
30 to 34
35 to 39
40 to 44
45 to 49
5 to 9
50 to 54
55 to 59
60 to 64
65 to 69
70 to 74
75 to 79
80 to 84
85 to 89
90 to 94
95 to 99
Age−standardized DALYs rate (per 100,000)
0
1000
2000
3000
1992−1996
1997−2001
2002−2006
2007−2011
2012−2016
2017−2021
Period
age
0 to 4
10 to 14
15 to 19
20 to 24
25 to 29
30 to 34
35 to 39
40 to 44
45 to 49
5 to 9
50 to 54
55 to 59
60 to 64
65 to 69
70 to 74
75 to 79
80 to 84
85 to 89
90 to 94
95 to 99
Age−standardized DALYs rate (per 100,000)
0
1000
2000
3000
1897
1902
1907
1912
1917
1922
1927
1932
1937
1942
1947
1952
1957
1962
1967
1972
1977
1982
1987
1992
1997
2002
2007
2012
2017
2022
Cohort
