# Supplementary material for: Applying Multiple Analyses to Quantify Changes in the Global, Regional, and National Burden of Tuberculosis From 1990 to 2021 and the Forecast Until 2035
Source: Can J Infect Dis Med Microbiol. 2025 Nov 20;2025:8899003. doi: 10.1155/cjid/8899003 (PMC12659984; doi:10.1155/cjid/8899003)
Supplement: Supporting Information — Additional supporting information can be found online in the Supporting Information section. [file 8899003.f1.zip › GBD_TB.zip!/GBD_TB_2021_upload/result/APC/TB_period-on-age_trend.pdf]

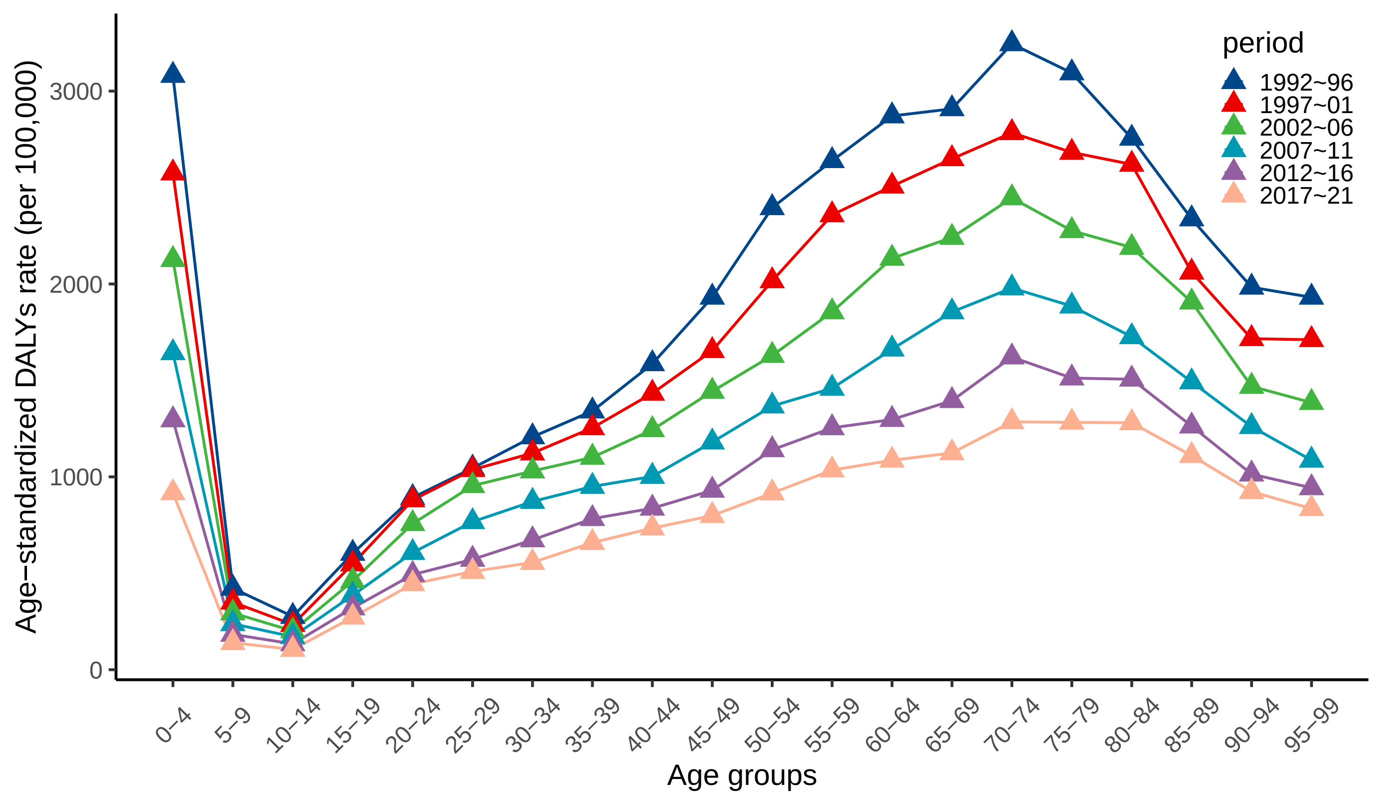

period
1992~96
1997~01
2002~06
2007~11
2012~16
2017~21
Age−standardized DALYs rate (per 100,000)
0
1000
2000
3000
0−4
5−9
10−14
15−19
20−24
25−29
30−34
35−39
40−44
45−49
50−54
55−59
60−64
65−69
70−74
75−79
80−84
85−89
90−94
95−99
Age groups
