# Supplementary material for: Applying Multiple Analyses to Quantify Changes in the Global, Regional, and National Burden of Tuberculosis From 1990 to 2021 and the Forecast Until 2035
Source: Can J Infect Dis Med Microbiol. 2025 Nov 20;2025:8899003. doi: 10.1155/cjid/8899003 (PMC12659984; doi:10.1155/cjid/8899003)
Supplement: Supporting Information — Additional supporting information can be found online in the Supporting Information section. [file 8899003.f1.zip › GBD_TB.zip!/GBD_TB_2021_upload/result/APC/TB_period-on-cohort_trend.pdf]

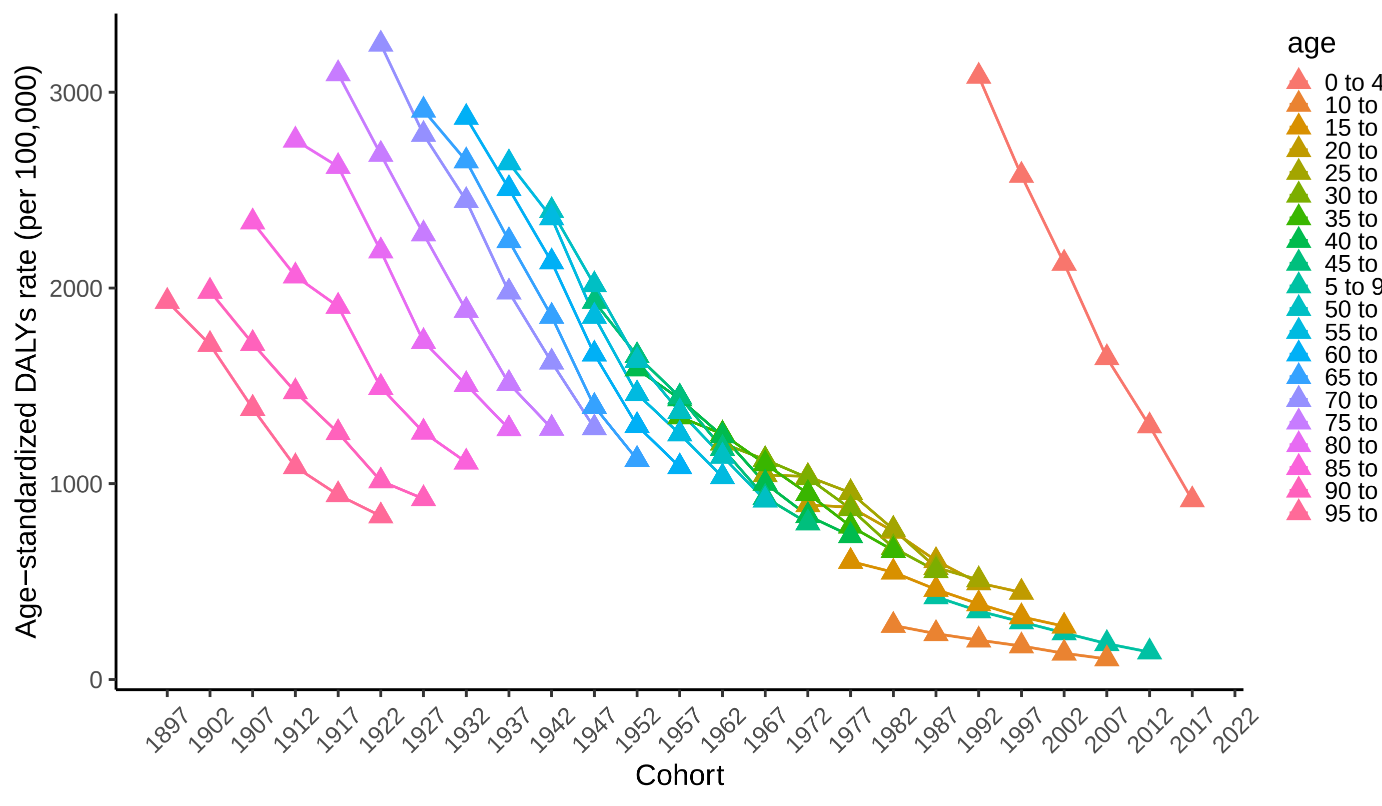

Age−standardized DALYs rate (per 100,000)
0
1000
2000
3000
1897
1902
1907
1912
1917
1922
1927
1932
1937
1942
1947
1952
1957
1962
1967
1972
1977
1982
1987
1992
1997
2002
2007
2012
2017
2022
Cohort
age
0 to 4
10 to
15 to
20 to
25 to
30 to
35 to
40 to
45 to
5 to 9
50 to
55 to
60 to
65 to
70 to
75 to
80 to
85 to
90 to
95 to

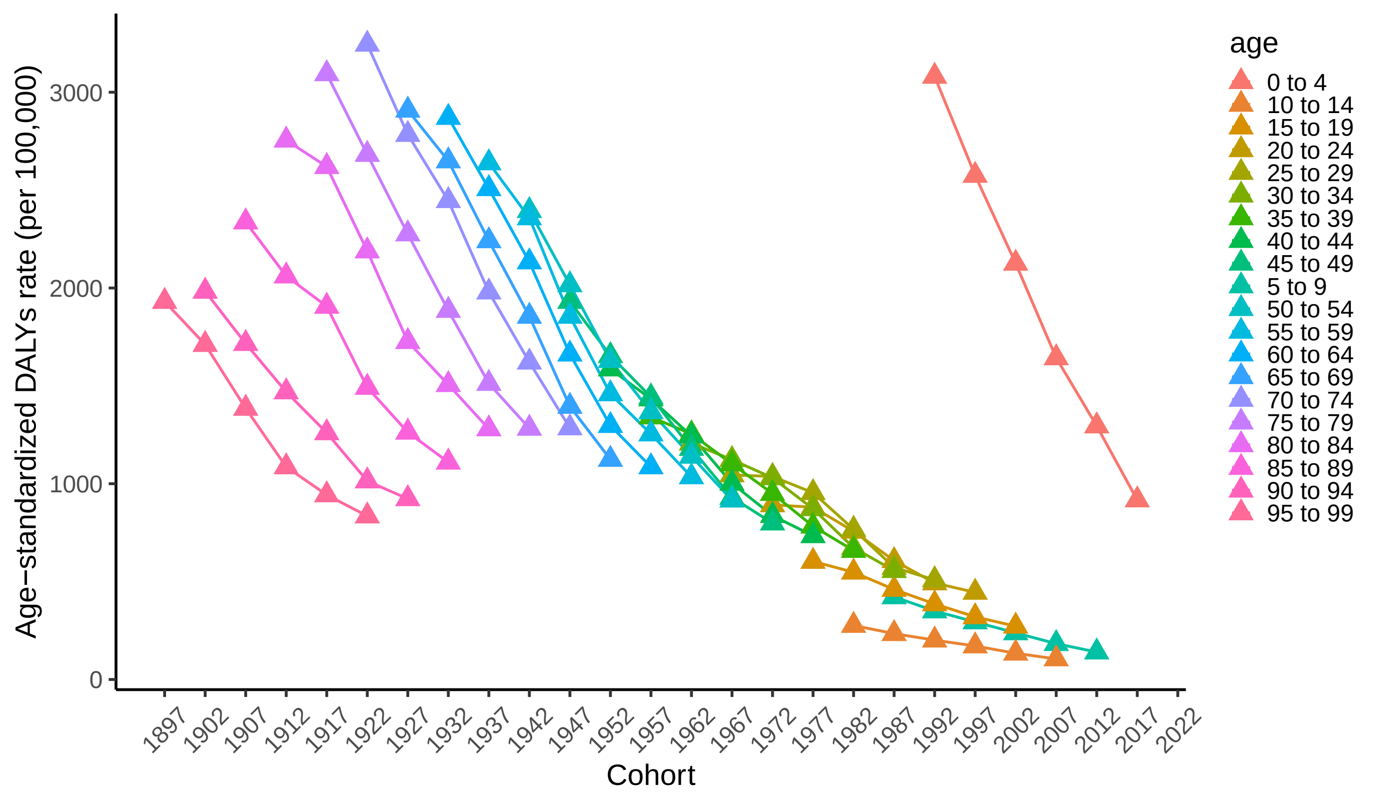

Age−standardized DALYs rate (per 100,000)
3000
2000
1000
0
1897
1902
1907
1912
1917
1922
1927
1932
1937
1942
1947
1952
1957
1962
1967
1972
1977
1982
1987
1992
1997
2002
2007
2012
2017
2022
Cohort
age
0 to 4
10 to 14
15 to 19
20 to 24
25 to 29
30 to 34
35 to 39
40 to 44
45 to 49
5 to 9
50 to 54
55 to 59
60 to 64
65 to 69
70 to 74
75 to 79
80 to 84
85 to 89
90 to 94
95 to 99

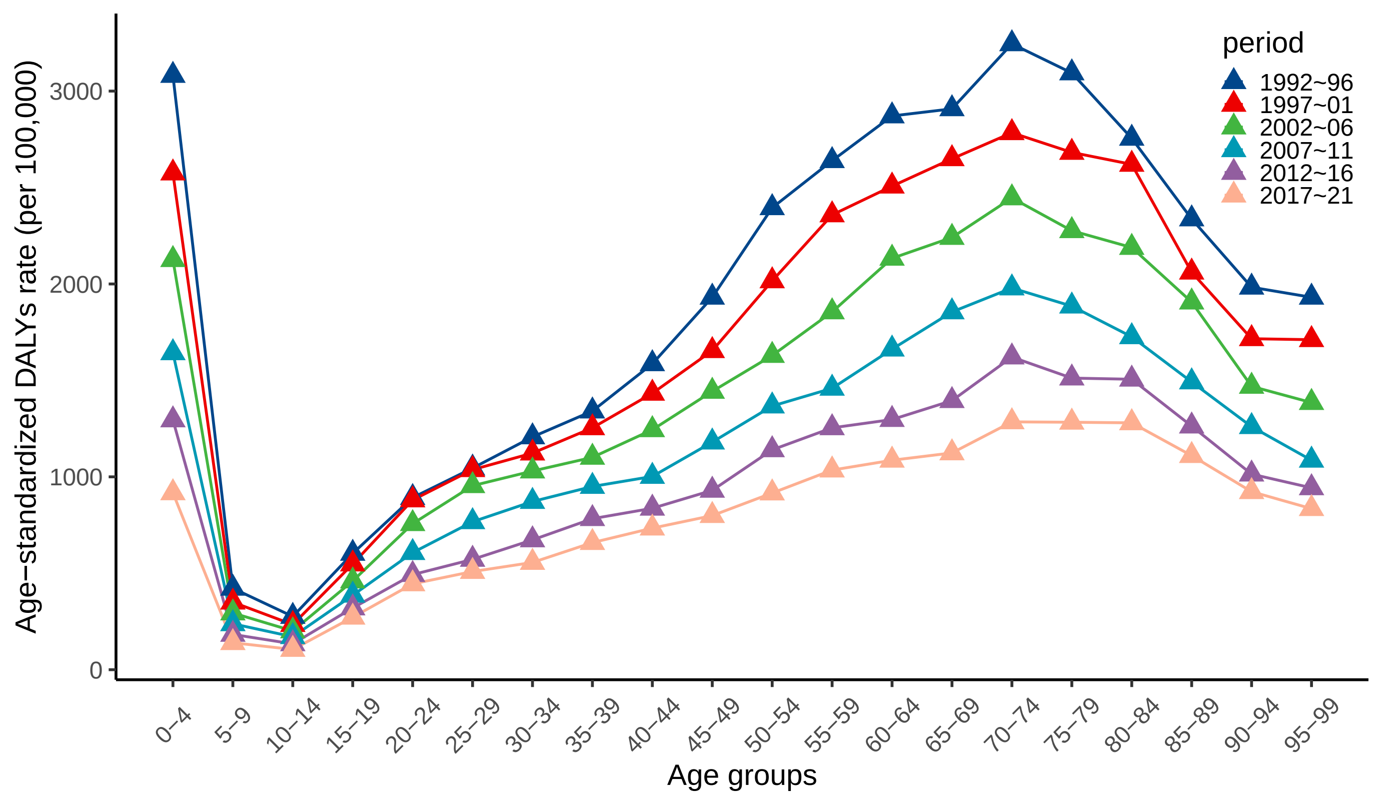

period
1992~96
1997~01
2002~06
2007~11
2012~16
2017~21
Age−standardized DALYs rate (per 100,000)
3000
2000
1000
0
0−4
5−9
10−14
15−19
20−24
25−29
30−34
35−39
40−44
45−49
50−54
55−59
60−64
65−69
70−74
75−79
80−84
85−89
90−94
95−99
Age groups

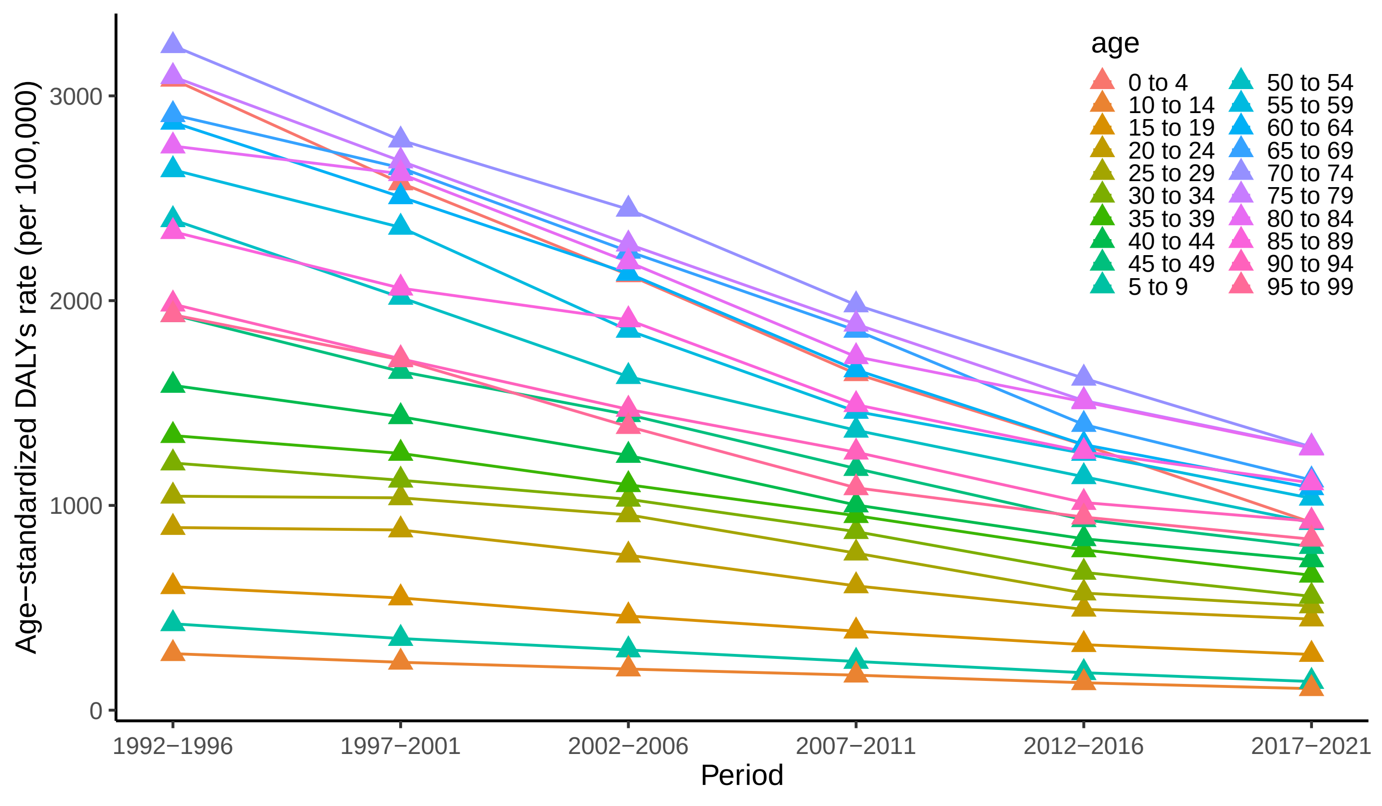

Age−standardized DALYs rate (per 100,000)
0
1000
2000
3000
1992−1996
1997−2001
2002−2006
2007−2011
2012−2016
2017−2021
Period
age
0 to 4
10 to 14
15 to 19
20 to 24
25 to 29
30 to 34
35 to 39
40 to 44
45 to 49
5 to 9
50 to 54
55 to 59
60 to 64
65 to 69
70 to 74
75 to 79
80 to 84
85 to 89
90 to 94
95 to 99
