# Supplementary material for: Applying Multiple Analyses to Quantify Changes in the Global, Regional, and National Burden of Tuberculosis From 1990 to 2021 and the Forecast Until 2035
Source: Can J Infect Dis Med Microbiol. 2025 Nov 20;2025:8899003. doi: 10.1155/cjid/8899003 (PMC12659984; doi:10.1155/cjid/8899003)
Supplement: Supporting Information — Additional supporting information can be found online in the Supporting Information section. [file 8899003.f1.zip › GBD_TB.zip!/GBD_TB_2021_upload/result/BAPC/TB_BAPC_17Southern Latin America.pdf]

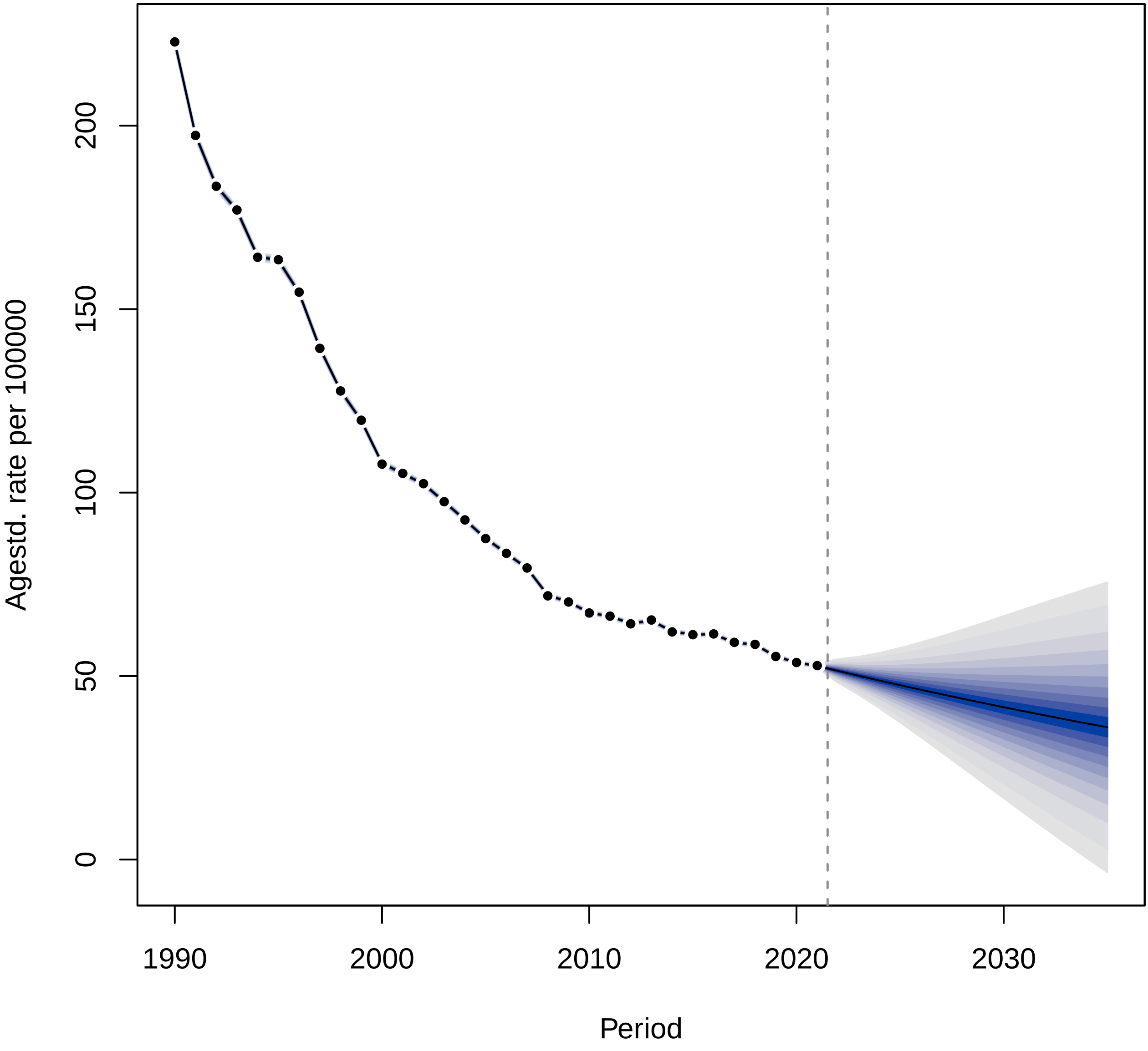

Agestd. rate per 100000
0
50
100
150
200
1990
2000
2010
2020
2030
Period
