# Supplementary material for: Applying Multiple Analyses to Quantify Changes in the Global, Regional, and National Burden of Tuberculosis From 1990 to 2021 and the Forecast Until 2035
Source: Can J Infect Dis Med Microbiol. 2025 Nov 20;2025:8899003. doi: 10.1155/cjid/8899003 (PMC12659984; doi:10.1155/cjid/8899003)
Supplement: Supporting Information — Additional supporting information can be found online in the Supporting Information section. [file 8899003.f1.zip › GBD_TB.zip!/GBD_TB_2021_upload/result/decomposition/TB_regions_decomposition.pdf]

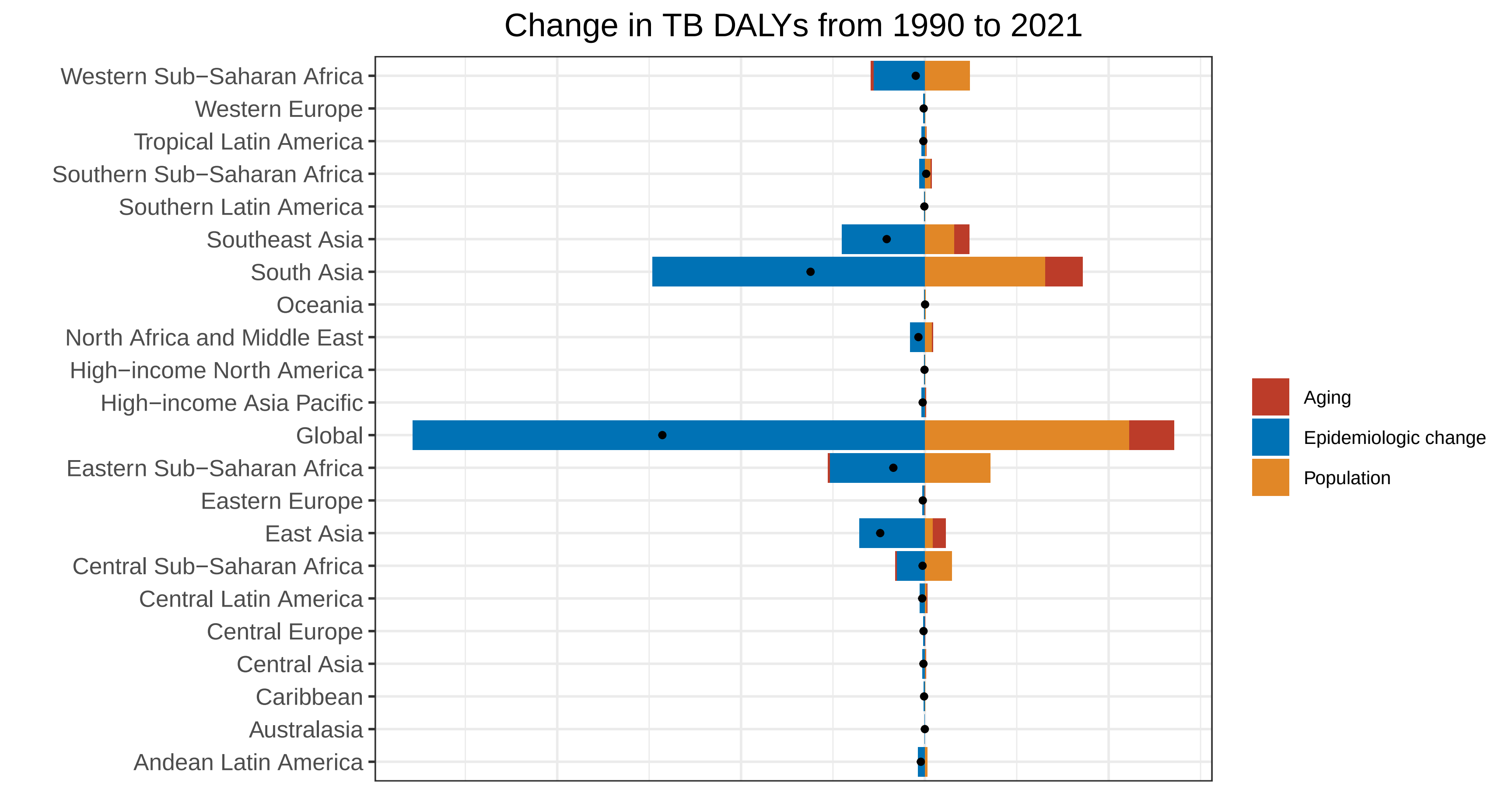
Change in TB DALYs from 1990 to 2021
Western Sub−Saharan Africa
Western Europe
Tropical Latin America
Southern Sub−Saharan Africa
Southern Latin America
Southeast Asia
South Asia
Oceania
North Africa and Middle East
High−income North America
High−income Asia Pacific
Global
Eastern Sub−Saharan Africa
Eastern Europe
East Asia
Central Sub−Saharan Africa
Central Latin America
Central Europe
Central Asia
Caribbean
Australasia
Andean Latin America
Aging
Epidemiologic change
Population
