# Supplementary material for: Applying Multiple Analyses to Quantify Changes in the Global, Regional, and National Burden of Tuberculosis From 1990 to 2021 and the Forecast Until 2035
Source: Can J Infect Dis Med Microbiol. 2025 Nov 20;2025:8899003. doi: 10.1155/cjid/8899003 (PMC12659984; doi:10.1155/cjid/8899003)
Supplement: Supporting Information — Additional supporting information can be found online in the Supporting Information section. [file 8899003.f1.zip › GBD_TB.zip!/GBD_TB_2021_upload/result/frontier/TB_boostrap_frontier_100times.pdf]

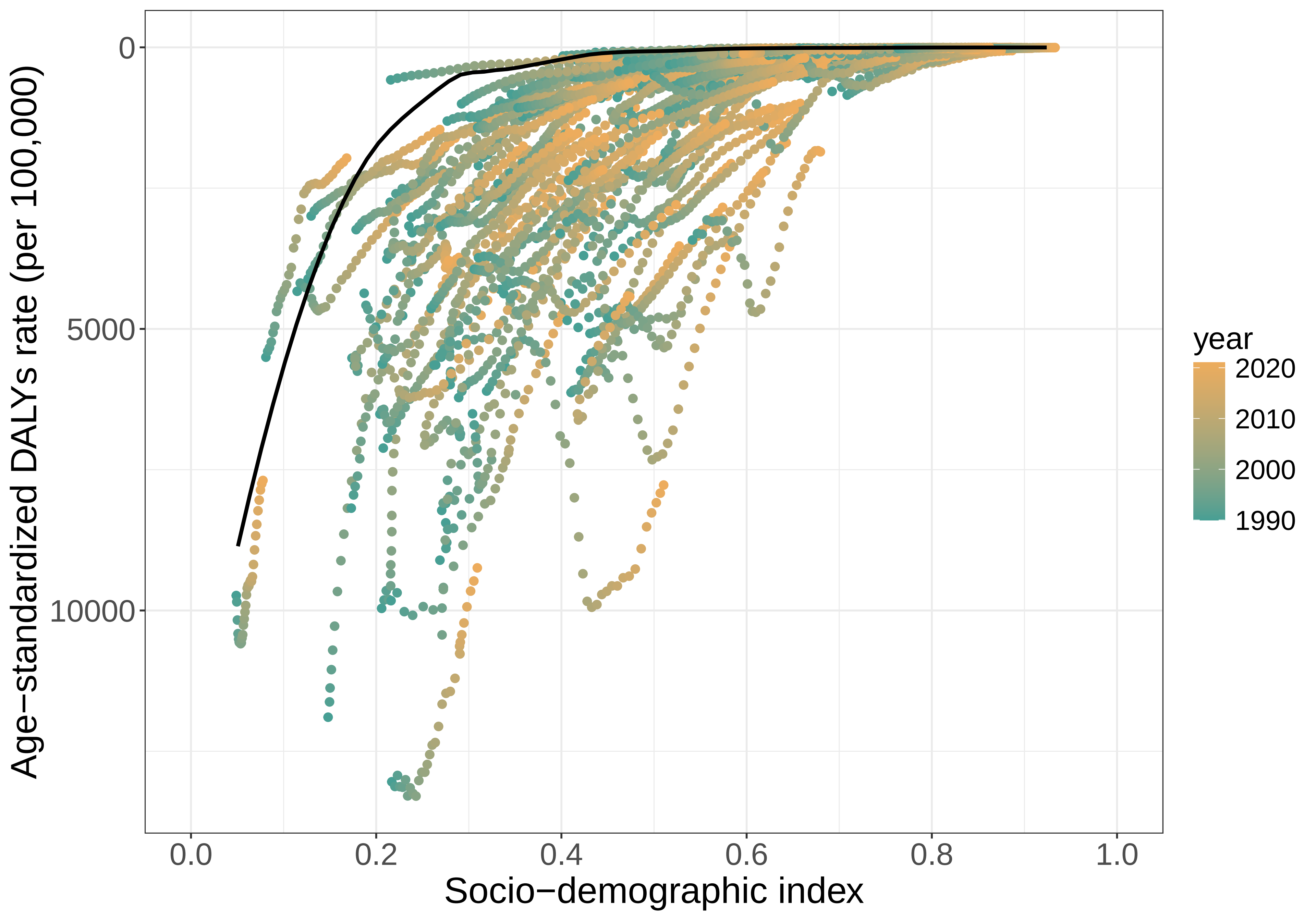

0
5000
10000
Age−standardized DALYs rate (per 100,000)
0.0
0.2
0.4
0.6
0.8
1.0
Socio−demographic index
year
2020
2010
2000
1990
