# Supplementary material for: Applying Multiple Analyses to Quantify Changes in the Global, Regional, and National Burden of Tuberculosis From 1990 to 2021 and the Forecast Until 2035
Source: Can J Infect Dis Med Microbiol. 2025 Nov 20;2025:8899003. doi: 10.1155/cjid/8899003 (PMC12659984; doi:10.1155/cjid/8899003)
Supplement: Supporting Information — Additional supporting information can be found online in the Supporting Information section. [file 8899003.f1.zip › GBD_TB.zip!/GBD_TB_2021_upload/result/inequality/TB_DALY_SII.pdf]

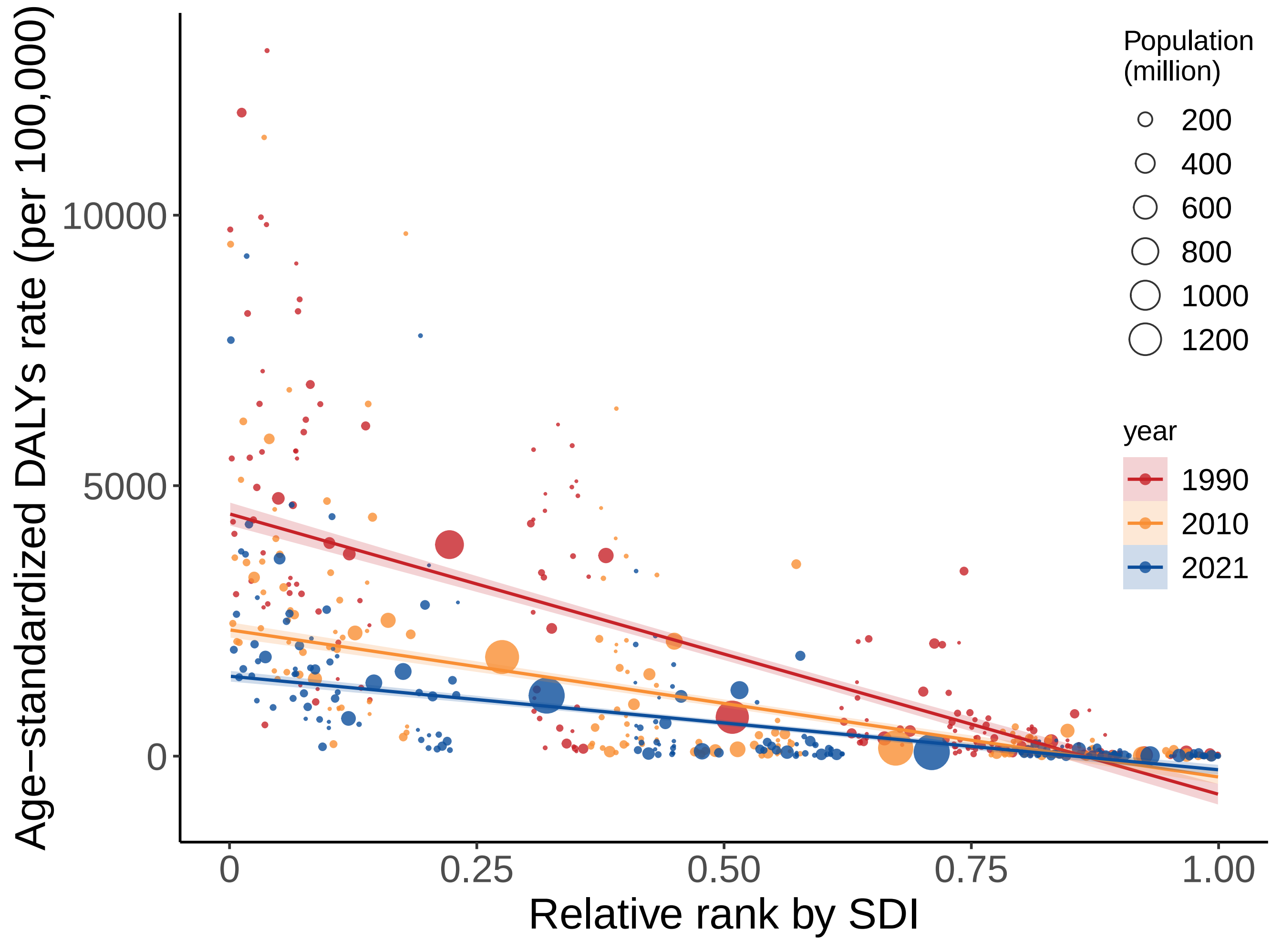
Age−standardized DALYs rate (per 100,000)
10000
5000
0
0
0.25
0.50
0.75
1.00
Relative rank by SDI
Population (million)
200
400
600
800
1000
1200
year
1990
2010
2021
